# Experimental astrochemistry and machine learning unravel the formation of oxygen-bearing organic molecules in extraterrestrial ices

**DOI:** 10.1038/s42004-026-02107-2

**Published:** 2026-07-14

**Authors:** Xilin Bai, Sixuan Mi, Qi’ang Gong, Jinghui Lu, Jiuzhong Yang, Yang Pan, Xiaohu Li, Zhenrong Sun, Tong Zhu, Ralf I. Kaiser, Tao Yang

**Affiliations:** 1 State Key Laboratory of Precision Spectroscopy, East China Normal University, Shanghai, 200062 China; 2 Shanghai Engineering Research Center of Molecular Therapeutics and New Drug Development, School of Chemistry and Molecular Engineering, East China Normal University, Shanghai, 200062 China; 3 National Synchrotron Radiation Laboratory, University of Science and Technology of China, Hefei, Anhui 230029 China; 4 State Key Laboratory of Radio Astronomy and Technology, Xinjiang Astronomical Observatory, Chinese Academy of Sciences, Urumqi, Xinjiang 830011 China; 5 Department of Chemistry, University of Hawaii at Manoa, Honolulu, Hawaii 96822 USA; 6 Collaborative Innovation Center of Extreme Optics, Shanxi University, Taiyuan, Shanxi 030006 China

**Keywords:** Reaction kinetics and dynamics, Origin of life, Chemical physics

## Abstract

Oxygen-bearing organic molecules, including aldehydes, alcohols, and peroxides, serve as key precursors to complex organics in extraterrestrial environments. Their non-equilibrium formation mechanisms critically constrain the molecular complexity available for prebiotic chemistry. Here we introduce a methodology combining astrochemical simulation experiments with machine learning techniques, to unravel the composition and formation mechanisms of complex organics synthesized in methane (CH_4_) – molecular oxygen (O_2_) ices exposed to proxies of galactic cosmic rays. Exploiting synchrotron vacuum ultraviolet photoionization mass spectrometry (SVUV-PI-ReToF-MS), we identified a rich inventory of oxygen-bearing organic molecules in the temperature-programmed desorption (TPD) phase through photoionization efficiency (PIE) curve fitting and isotopic labeling experiments. Non-equilibrium formation mechanisms were discovered by employing neural network potentials in combination with advanced reaction path search algorithms. This integration of machine learning with astrochemical experiments is anticipated to revolutionize our capabilities in predicting the molecular inventory in dust grain ice mantles, thus advancing our fundamental knowledge of the molecular evolution in the new astrochemical ice age.

## Abstract (web summary)

Predicting the molecular inventory of dust grain ice mantles through in situ structural identification remains highly challenging, highlighting the need for systematic and automated exploration strategies. Here, the authors combine synchrotron vacuum ultraviolet photoionization reflectron time-of-flight mass spectrometry (SVUV-PI-ReToF-MS), neural network potentials, and advanced reaction path search algorithms to unveil the molecular inventory and non-equilibrium formation mechanisms of oxygen-bearing organic molecules in methane–molecular oxygen ices exposed to galactic cosmic ray proxies.

## Introduction

The untangling of the formation mechanism of complex organic molecules (COMs) in extraterrestrial environments has played a critical role in unraveling the astrochemical evolution of bio-related species relevant to the origin-of-life theme^1–5^. Gas-phase simulation experiments combined with astrochemical modeling studying elementary reactions that probe fundamental mechanisms of interstellar molecules such as structural isomers of acetaldehyde (CH_3_CHO) and vinyl alcohol (C_2_H_3_OH)^6^ and the isomeric triplet propylene oxide (C_3_H_6_O), acetone (CH_3_COCH_3_), and propanal (C_2_H_5_CHO)^7^—molecules that are also considered key prebiotic precursors potentially facilitating the abiotic formation of more complex biomolecules (e.g., sugars and polyols) in extraterrestrial environments—cannot replicate astronomically observed fractional abundances, often yielding abundances a few orders of magnitude below their observed values. Even within gas–grain coupled models, where radical–radical recombination pathways successfully reproduce species such as methyl formate, significant discrepancies—often spanning several orders of magnitude—persist between predicted abundances and astronomical observations^8–10^. These inconsistencies suggest that conventional thermal diffusion–driven chemistry at 10–20 K may underestimate the contribution of non-thermal processes, particularly those induced by galactic cosmic rays (GCRs). This discrepancy becomes especially evident when considering observations in chemically rich regions such as Sgr B2(N) and the shocked molecular cloud G + 0.693, where molecular complexity is closely linked to the release of ice mantle constituents into the gas phase. Whether through thermal desorption in hot cores or sputtering processes in shocked environments, these mechanisms establish a direct connection between solid-state ice chemistry and gas-phase molecular inventories. Consequently, icy dust grains—nanoparticles coated with molecular ices composed of species such as water (H_2_O), ammonia (NH_3_), methane (CH_4_), carbon monoxide (CO), carbon dioxide (CO_2_), and methanol (CH_3_OH)—play a critical role in the early stages of star formation as well as the prebiotic origin of extraterrestrial life^11–13^. These nanoparticles are often dubbed molecular factories and hence facilitate an abiotic formation of complex organics upon exposure to, e.g., GCRs, ultraviolet (UV) photons, solar wind, and X-rays prior to their sublimation into the gas phase once star formation begins^14–16^. The bulk chemistry on these icy dust grains mirrors a sophisticated chemical reaction network and rich molecular inventory^5,17^ thus enabling a unique opportunity to incorporate these results into astrochemical reaction network with non-equilibrium reaction mechanisms and molecular species to further constrain the uncertainties between the modeling and observation.

Astrophysically simulated ices at cryogenic temperatures of typically 10 K in cold, dark clouds and icy objects in the outer Solar system are monitored by Fourier-transform infrared spectroscopy (FTIR)^13^, ultraviolet−visible (UV−Vis) spectroscopy^18^ and/or Raman spectroscopy^19^ to identify the functional groups of COMs during the irradiation processes in the condensed phase. Following the temperature-programmed desorption (TPD) of the processed ices, the subliming COMs are characterized by millimeter and submillimeter spectroscopy (MM/subMMS)^20^, electron-impact ionization quadrupole mass spectrometry (EI-QMS)^21^, resonance-enhanced multiphoton ionization (REMPI), and more recently isomer selective single-photon ionization coupled with reflectron time-of-flight mass spectrometry (ReToF-MS)^22^. In particular, the exploitation of the four-wave-mixing single-photon ionization coupled with ReToF-MS opens up a unique methodology to discriminate structural isomers, which are further supported by high-level ab initio calculations to determine the adiabatic ionization energies and potential energy surfaces to infer the underlying reaction mechanisms. However, the simultaneous structural identification of subliming products *in* *situ* still remains a major challenge in unraveling the molecular complexities of the irradiated ice mantles. The molecular reaction network triggered by non-equilibrium processes is inherently complex, involving numerous transient radicals and competing pathways on a high-dimensional potential energy surface. This complicates both product distributions and mechanistic assignments, thereby motivating the need for systematic, automated exploration strategies.

In this study, we unravel the molecular complexities in the methane (CH_4_) – oxygen (O_2_) astrophysical ice analogs under energetic-electron irradiation, demonstrating the explicit formation of aldehydes (formaldehyde (H_2_CO), acetaldehyde (CH_3_CHO)), alcohols (methanol (CH_3_OH), ethanol (C_2_H_5_OH), and polyols like methanediol (CH_2_(OH)_2_) and methanetriol (CH(OH)_3_)), peroxides (hydrogen peroxide (HOOH), methyl hydroperoxide (CH_3_OOH), ethyl hydroperoxide (C_2_H_5_OOH), and keto-hydroperoxides including hydroperoxyl acetaldehyde (HOOCH_2_CHO) and hydroperoxymethyl formate (HOOCH_2_OCHO)), ethers (dimethyl ether (CH_3_OCH_3_), and methoxymethanol (CH_3_OCH_2_OH)), formic acid (HCOOH) and even radicals (methoxy radical (CH_3_O) and methylperoxy radical (CH_3_OO)). Facilitated by synchrotron vacuum ultraviolet photoionization reflectron time-of-flight mass spectrometry (SVUV-PI-ReToF-MS)^23–26^, we identified the above-mentioned molecular species by distinguishing TPD profiles at selected photon energies, comparing isotopically labeled TPD profiles between CH_4_–O_2_, CD_4_–O_2_, and CH_4_–^18^O_2_ ice analogs, as well as photoionization efficiency (PIE) curve fitting exploiting tunable photon energies. The underlying molecular formation mechanisms were systematically explored by employing neural network potentials^27^ combined with the automated reaction path search program KinBot^28,29^, which enabled the efficient identification of plausible intermediates, products, and transition states within the ice analog systems. These candidate pathways, including those relevant to surface-mediated reactions on icy grains, were subsequently validated and refined using the high-level quantum chemical method CCSD(T)/CBS//ωB97X-D/def2-TZVP^30–32^. By merging the experimental product identification via SVUV-PI-ReToF-MS with machine-learning–accelerated reaction network discovery and rigorous ab initio benchmarking, this study provides a comprehensive simulation of the complex formation routes of COMs in extraterrestrial CH_4_–O_2_ ices, thus offering a protocol in resolving long-standing chemical uncertainties in extraterrestrial ice chemistry, once described as “the last refuge of the scoundrel”^33^.

## Results

### Temperature-programmed desorption (TPD) profiles at selected photon energies

**Fig. 1:**
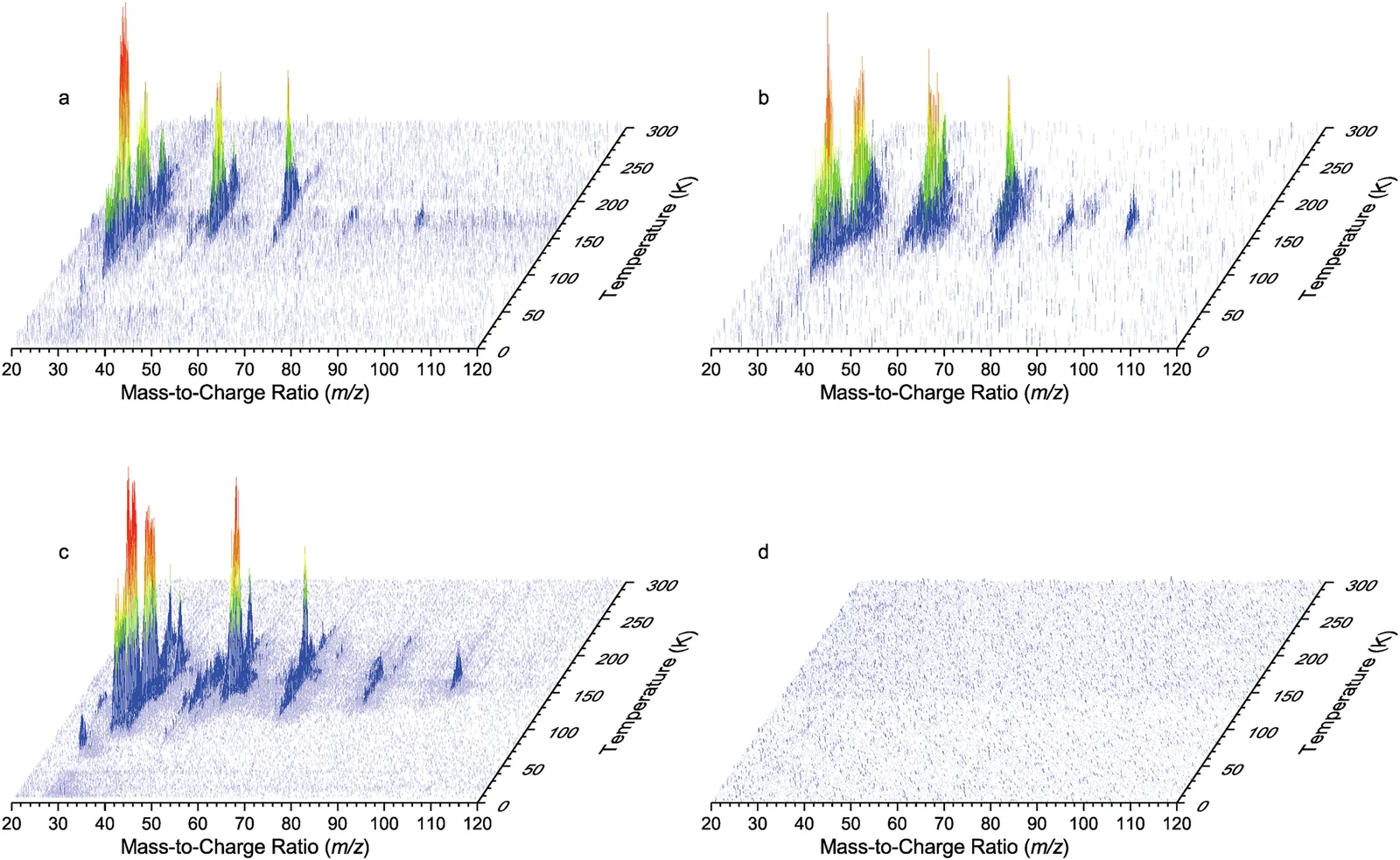
Synchrotron vacuum ultraviolet photoionization reflectron time-of-flight mass spectrometry (SVUV-PI-ReToF-MS) at the photon energy of 11.50 eV. SVUV-PI-ReToF-MS data recorded during the temperature-programmed desorption (TPD) phase of the irradiated **a** CH_4_–O_2_, **b** CD_4_–O_2_, **c** CH_4_–^18^O_2_, and the unirradiated **d** CH_4_–O_2_ ices at 11.50 eV.

**Fig. 2:**
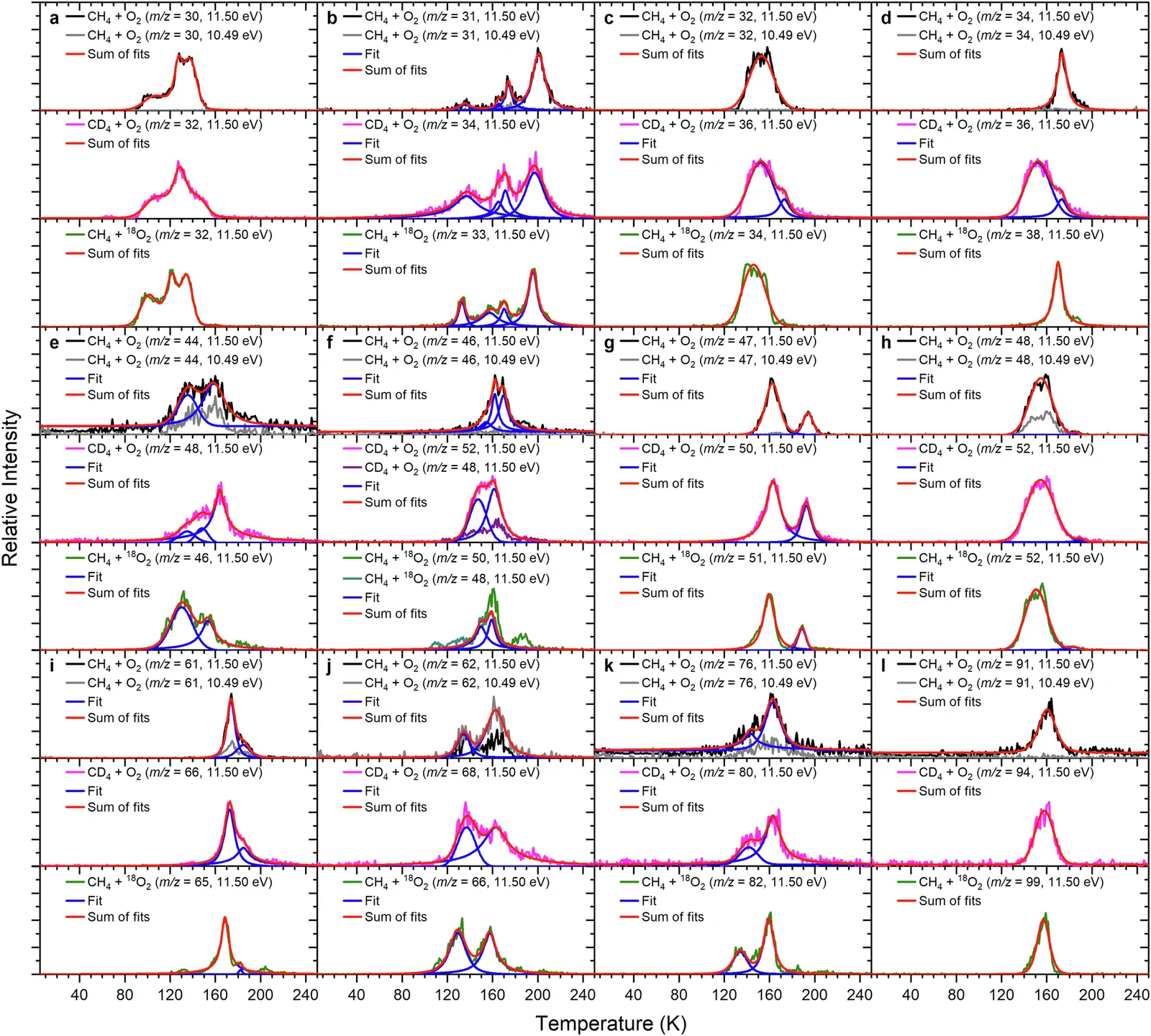
Temperature-programmed desorption (TPD) profiles. TPD profiles for **a** *m/z* = 30, **b** *m/z* = 31, **c** *m/z* = 32, **d** *m/z* = 34, **e** *m/z* = 44, **f** *m/z* = 46, **g** *m/z* = 47, **h** *m/z* = 48, **i** *m/z* = 61, **j** *m/z* = 62, **k** *m/z* = 76, and **l** *m/z* = 91 of the irradiated CH_4_–O_2_, CD_4_–O_2_, and CH_4_–^18^O_2_ ices at selected photon energies.

We employed the SVUV-PI-ReToF-MS technique^23–26^ to collect TPD profiles of molecular species generated in the CH_4_–O_2_, CD_4_–O_2_ and CH_4_–^18^O_2_ ices at selected photon energies (Fig. 1, Supplementary Fig. 1). Two representative photon energies (10.49 and 11.50 eV) were selected to achieve soft ionization while still providing sufficient coverage for species of higher ionization energies. At the SVUV photon energy (PE) of 11.50 eV, we detected subliming products after photoionization at mass-to-charge ratios (*m/z*) up to 91 in the irradiated CH_4_–O_2_ ice, 94 in the irradiated CD_4_–O_2_ ice, and 99 in the irradiated CH_4_–^18^O_2_ ice, respectively, and contrasted these data with product distribution in the pristine, non-processed ice (Fig. 1). Considering the elemental composition (C, H, O) of our reaction system, the extracted TPD profiles of the newly-formed products in the CH_4_–O_2_ ice exhibit ion signals at *m/z* = 30 (CH_2_O^+^/C_2_H_6_^+^), 31 (CH_3_O^+^/C_2_H_7_^+^), 32 (CH_4_O^+^/O_2_^+^), 34 (H_2_O_2_^+^), 44 (CO_2_^+^/C_2_H_4_O^+^/C_3_H_8_^+^), 46 (CH_2_O_2_^+^/C_2_H_6_O^+^), 47 (CH_3_O_2_^+^/C_2_H_7_O^+^), 48 (CH_4_O_2_^+^/C_4_^+^/O_3_^+^), 61 (CHO_3_^+^/C_2_H_5_O_2_^+^/C_3_H_9_O^+^/C_5_H^+^), 62 (CH_2_O_3_^+^/ C_2_H_6_O_2_^+^/C_5_H_2_^+^), 76 (CO_4_^+^/C_2_H_4_O_3_^+^/C_3_H_8_O_2_^+^/C_5_O^+^/C_6_H_4_^+^), and 91 (C_2_H_3_O_4_^+^/C_3_H_7_O_3_^+^/C_4_H_11_O_2_^+^/ C_6_H_3_O^+^/C_7_H_7_^+^) (Fig. 2).

First, ethane (C_2_H_6_), oxygen (O_2_), carbon dioxide (CO_2_), and ozone (O_3_) cannot be ionized at a photon energy of 11.50 eV thus offering no contribution to ion signals at *m/z* = 30, 32, 44 and 48, respectively^34–36^. Secondly, molecular ions of hydrocarbons including C_2_H_7_^+^, C_3_H_8_^+^, C_5_H^+^, C_5_H_2_^+^, C_6_H_4_^+^, and C_7_H_7_^+^ are not formed or detected in our irradiated CH_4_–O_2_ ice, since their isotopic counterparts were not detected in the CD_4_–O_2_ or CH_4_–^18^O_2_ ice. The finding correlates well with the scarcity of hydrocarbons in both CH_4_:O_2_ (1:1) and CH_4_:O_2_ (1:100) ice mixtures^37^. Thirdly, the absence of ion signals at *m/z* of 54 (C_2_D_7_O^+^), 62 (CDO_3_^+^), 70 (C_3_D_9_O^+^), 76 (CO_4_^+^ and C_5_O^+^), 98 (C_3_D_7_O_3_^+^), and 102 (C_4_D_11_O_2_^+^) in the CD_4_–O_2_ ice precludes the possible detection of C_2_H_7_O^+^, CHO_3_^+^, C_3_H_9_O^+^, CO_4_^+^, C_5_O^+^, C_3_H_7_O_3_^+^, and C_4_H_11_O_2_^+^ in the CH_4_–O_2_ ice. Similarly, we did not observe ion signals at *m/z* of 68 (CH_2_^18^O_3_^+^) and 80 (C_3_H_8_^18^O_2_^+^) in the CH_4_–^18^O_2_ ice thus precluding the formation and detection of CH_2_O_3_^+^ along with C_3_H_8_O_2_^+^ in the CH_4_–O_2_ ice. Finally, overlapping TPD profiles at photon energies of 11.50 eV and 10.49 eV after scaling to the signal at *m/z* = 48 in the CH_4_–O_2_ ice imply that the C_4_^+^ cation is not formed or detected under current experimental sensitivity, since the three C_4_ isomers bear ionization energies of 10.87, 10.92, and 10.77 eV, respectively^38^. In sum, by comparing TPD profiles of selected photon energies and isotopic counterparts, we can assign the molecular ions in the CH_4_–O_2_ system as 30 (CH_2_O^+^), 31 (CH_3_O^+^), 32 (CH_4_O^+^), 34 (H_2_O_2_^+^), 44 (C_2_H_4_O^+^), 46 (CH_2_O_2_^+^/C_2_H_6_O^+^), 47 (CH_3_O_2_^+^), 48 (CH_4_O_2_^+^), 61 (C_2_H_5_O_2_^+^), 62 (C_2_H_6_O_2_^+^), 76 (C_2_H_4_O_3_^+^) and 91 (C_2_H_3_O_4_^+^) (Supplementary Table 1).

### Photoionization efficiency (PIE) curves

**Fig. 3:**
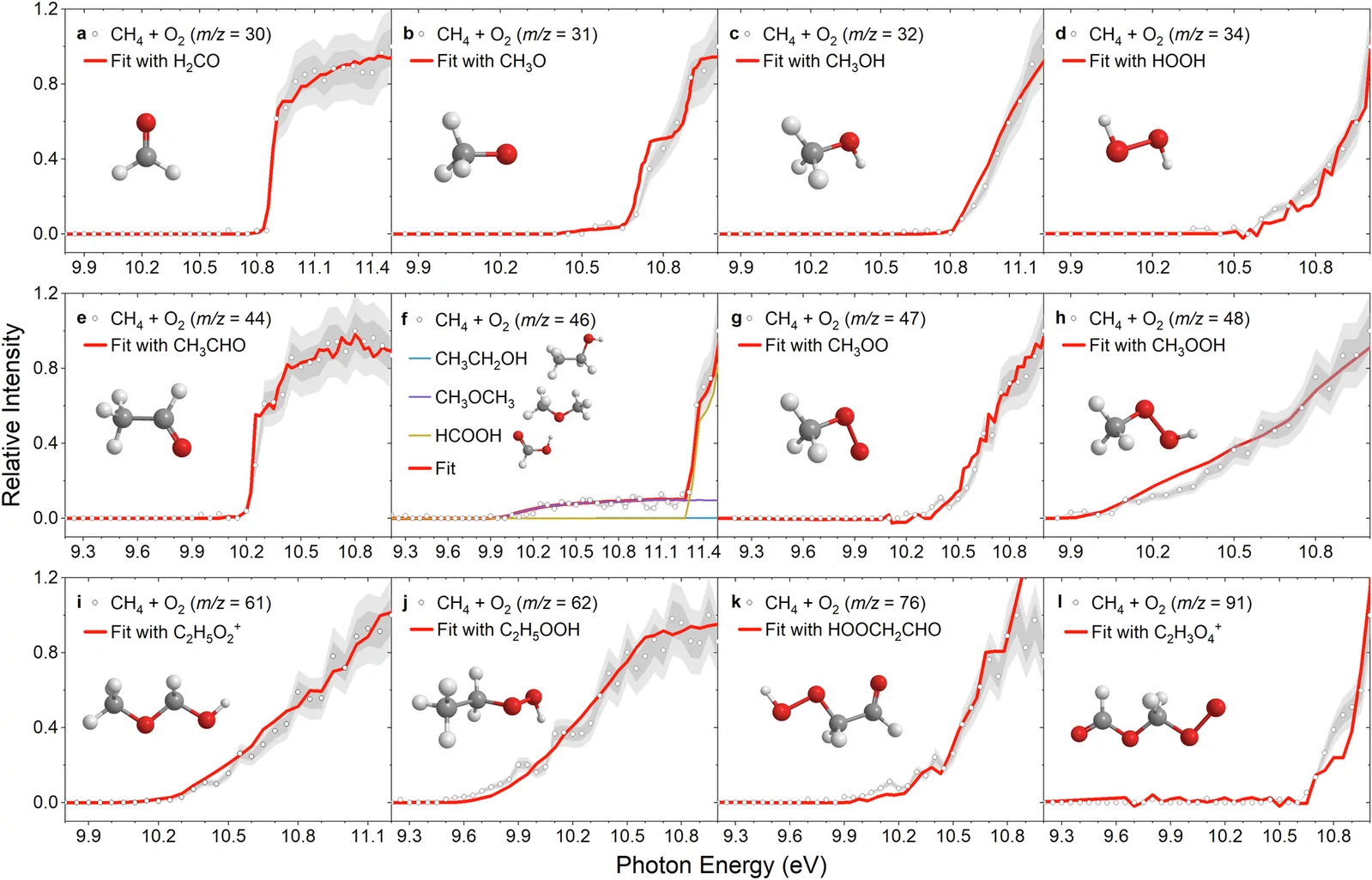
Photoionization efficiency (PIE) curves. PIE curves for the species linked to **a** *m/z* = 30, **b** *m/z* = 31, **c** *m/z* = 32, **d** *m/z* = 34, **e** *m/z* = 44, **f** *m/z* = 46, **g** *m/z* = 47, **h** *m/z* = 48, **i** *m/z* = 61, **j** *m/z* = 62, **k** *m/z* = 76, and **l** *m/z* = 91. The black dots represent experimental data, while the red lines indicate the fitting of the reference PIE curves. The dark-gray and light-gray shaded regions indicate the 1σ and 2σ standard deviations of the PIE curve (averaged across individual scans), respectively.

We further scanned the SVUV photon energy during the TPD phase in order to extract experimental PIE curves at distinct *m/z* values of subliming products; this allows an accurate identification of molecular species by fitting them to reference PIE curves^23^. PIE curves—representing ion signal intensity as a function of photon energy—were employed to achieve isomer-resolved identification by revealing characteristic ionization thresholds. To optimize the signal-to-noise ratio within the temporal constraints of the TPD experiments, four scanning ranges were implemented: 9.20–11.00 eV, 9.20–11.50 eV, 9.80–11.00 eV, and 9.80–11.50 eV. The selected photon energy ranges ensure coverage of ionization thresholds for all relevant species (Fig. 3).

Initially, PIE curves collected at *m/z* of 30, 31, 32 and 34 can likely be attributed to reference PIEs of formaldehyde (H_2_CO), methoxy radical (CH_3_O), methanol (CH_3_OH), and hydrogen peroxide (HOOH), whose ionization onsets of 10.85 ± 0.05 eV, 10.65 ± 0.05 eV, 10.80 ± 0.05 eV, and 10.60 ± 0.05 eV match well with the literature data of 10.88 ± 0.01 eV, 10.701 ± 0.005 eV, 10.84 ± 0.01 eV, and 10.631 ± 0.007 eV, respectively^39–41^. The TPD data at *m/z* = 44 (C_2_H_4_O^+^) can be deconvoluted into two Gaussian peaks; however, our PIE scanning between 120 and 170 K indicates the sole contribution from acetaldehyde (CH_3_CHO), not from vinyl alcohol (C_2_H_3_OH). At *m/z* = 46 (CH_2_O_2_^+^/C_2_H_6_O^+^), the overall experimental PIE curve can be fitted with a linear combination of reference PIE curves of ethanol (C_2_H_5_OH), dimethyl ether (CH_3_OCH_3_), and formic acid (HCOOH), which depict respective IEs of 10.48 ± 0.07 eV, 10.025 ± 0.025 eV and 11.33 ± 0.01 eV^39^.

We collected the PIE curve at *m/z* of 47 (CH_3_O_2_^+^) from 9.60 to 11.00 eV in steps of 0.05 eV from 140 to 180 K; this plot presents a distinct onset at 10.35 ± 0.05 eV that coincides with the adiabatic ionization energy of 10.33 ± 0.05 eV for the methylperoxy radical (CH_3_OO) formed in the reaction of CH_3_ with O_2_ conducted in a slow-flow reactor^42^. Although TPD profiles at *m/z* = 47 and 48 overlap between 140 and 180 K, the PIE curve at *m/z* of 48 depicts an ionization onset of 9.85 ± 0.05 eV and fits well with the reference PIE of methyl hydroperoxide (CH_3_OOH) up to 11.00 eV^43,44^, whose cation cannot fragment to CH_3_OO^+^ since such a dissociative channel remains closed at 11.64 eV^45^. The PIE curve at *m/z* = 61 (C_2_H_5_O_2_^+^) integrated from 160 to 180 K can be exceptionally well fitted with the PIE curve from the dissociative ionization of methoxymethanol (CH_3_OCH_2_OH) via hydrogen atom loss, displaying the appearance energy of 10.25 ± 0.05 eV^46^.

For PIE curves at *m/z* of 62 (C_2_H_6_O_2_^+^), 76 (C_2_H_4_O_3_^+^), and 91 (C_2_H_3_O_4_^+^) scanned in the range of 9.20 and 11.00 eV, structural isomers were consistent with ethyl hydroperoxide (C_2_H_5_OOH), hydroperoxyl acetaldehyde (HOOCH_2_CHO), and hydroperoxymethyl formate (HOOCH_2_OCHO) respectively^44,47,48^. At *m/z* = 62 (C_2_H_6_O_2_^+^), possible structural isomers like ethylene glycol (or 1,2-ethanediol, HOCH_2_CH_2_OH, IE = 9.85 ± 0.05 eV)^49^, and dimethyl peroxide (CH_3_OOCH_3_, IE = 9.31 eV)^50^ depict distinct onset IEs with respect to ethyl hydroperoxide (C_2_H_5_OOH; IE = 9.70 eV)^47^, while methoxymethanol (CH_3_OCH_2_OH) was found to follow prompt dissociative ionization via hydrogen atom loss to produce C_2_H_5_O_2_^+^ (*m/z* = 61, appearance energy 10.24 ± 0.05 eV)^46^. The adiabatic ionization energy for the most stable C_2_H_6_O_2_ isomer, *i*.*e*. 1,1-ethanediol (CH_3_CH(OH)_2_), was calculated to be 10.65 eV (Supplementary Table 2). The PIE curve at *m/z* of 76 (C_2_H_4_O_3_^+^) depicting an onset of 9.90 ± 0.05 eV precludes the formation of ethenetriol (HOCHC(OH)_2_, IE = 7.72‒8.03 eV) and dihydroxyacetaldehyde ((OH)_2_CHCHO, IE = 8.92‒9.83 eV), and hydroxymethyl formate (HC( = O)OCH_2_OH, IE = 10.50‒10.60 eV)^48,51^, glycolic acid (HOCH_2_COOH, IE = 10.00‒10.54 eV), and carbonic acid monomethyl ester (CH_3_OCOOH, IE = 10.62‒10.96 eV), but not hydroperoxyl acetaldehyde (HOOCH_2_CHO; IE = 9.75‒9.85 eV)^48^, which had been theoretically predicted as the most stable isomer on the potential energy surface^52^. The identification of *m/z* = 91 ion signals can be ascribed to hydroperoxymethyl formate (HOOCH_2_OCHO, 92 amu), which has been regarded as an intermediate and undergoes dissociative photoionization yielding C_2_H_3_O_4_^+^ at 10.65 ± 0.05 eV, aligning with its experimental observation of 10.60 ± 0.05 eV in the low-temperature oxidation of dimethyl ether studies^44,53^. The assigned species are those most consistent with both the experimental PIE data and the proposed reaction network. However, the competitive formation of minor isomers with similar ionization thresholds cannot be entirely excluded; thus, the assignments should be regarded as the most plausible under the present experimental constraints.

## Discussion

To address this complexity, we employed a machine learning–assisted workflow combining KinBot with neural network potentials to systematically explore intermediates and transition states across the potential energy surface. This strategy enables the identification of kinetically relevant pathways without relying on predefined reaction schemes, thereby capturing reaction channels that are difficult to access through conventional manual exploration and providing a more complete description of the electron-induced chemistry. The resulting reaction network not only reproduces well-established radical–radical recombination pathways (e.g., CH_3_O + H, CH_2_OH + H, and CH_3_ + OH) commonly invoked in astrochemical models^8–10^, but also reveals additional low-barrier channels that contribute to the formation of oxygen-bearing organic molecules. In particular, the barrierless insertion of O(^1^D) into CH_4_ is independently confirmed, supporting its role as a primary formation pathway in CH_4_-bearing ices. By comparing these pathways with previously proposed mechanisms, such as the formation of HCOOH via HCO + OH, the present approach provides a more complete mapping of the reaction network and helps to rationalize the experimentally observed product distribution.

**Fig. 4:**
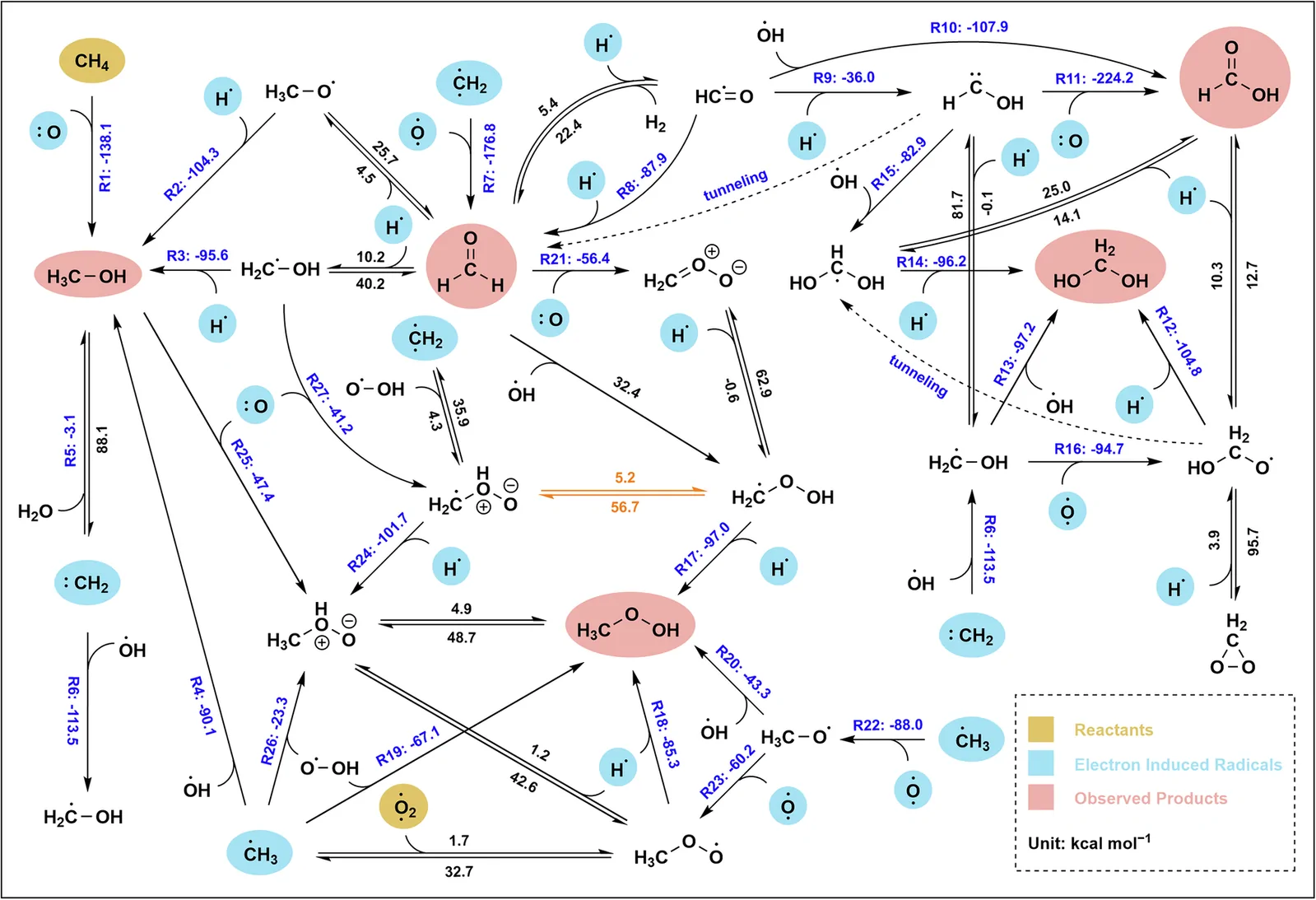
Calculated reaction network for the CH_4_–O_2_ system to produce first-generation C1 products. Reactants, electron-induced radicals, and observed products are shown in yellow, blue, and red backgrounds, respectively. E/Z configurations are not distinguished in the scheme. The number adjacent to each arrow refers to the reaction energy with zero-point vibrational correction in kcal mol^–1^. The reactions indicated in black and blue were calculated at the level of CCSD(T)/CBS//ωB97X-D/def2-TZVP, with those barrierless reactions labeled as R1-R27. The reactions indicated in orange could not be located at such levels and were calculated at CCSD(T)/CBS//MP2/6-31G(d).

Building on this reaction framework, we further rationalized the formation of COMs such as aldehydes, alcohols, and peroxides formed in the CH_4_–O_2_ ice using the molecular reaction network constructed by integrating neural network potentials with the automated reaction path search program KinBot (Fig. 4 and Supplementary Figs. 2–3). While Fig. 4 details the reaction network initiated by electron-induced radicals, Supplementary Fig. 2c serves as a control analysis to verify the stability of the identified intermediates. Upon interaction with energetic electrons, CH_4_ and O_2_ can follow unimolecular decomposition to yield methyl (CH_3_) and suprathermal hydrogen (H), and two ground-state oxygen atoms [O(^3^P)] or O(^3^P) plus electronically excited O(^1^D), respectively^54^. Unimolecular decomposition of CH_3_ or recombination of CH_3_ with O(^3^P) can lead to CH_2_ or the vibrationally excited methoxy radical CH_3_O, respectively, while atomic hydrogen can combine with O or O_2_ to form hydroxyl (OH) or hydroperoxyl (HOO) radical. The barrierless formation of hydrogen peroxide (HOOH) can be achieved via radical-radical recombination of two hydroxyl (OH) or hydroperoxyl (HOO) radicals with atomic hydrogen (Supplementary Fig. 2).

The barrierless insertion of O(^1^D) into CH_4_ to generate methanol (CH_3_OH) has been well investigated and regarded as the predominant reaction pathway in simulated astrophysical CH_4_–O_2_ ices (R1: −138.1 kcal mol^−1^)^55,56^. Other energetic pathways leading to CH_3_OH can be accessed via the exoergic reaction of ground state CH_3_O with H (R2: −104.3 kcal mol^−1^), H_2_COH with H (R3: −95.6 kcal mol^−1^), CH_3_ with OH (R4: −90.1 kcal mol^−1^), or CH_2_ with H_2_O (R5: −3.1 kcal mol^−1^). It is noted that the hydroxymethyl radical CH_2_OH can be formed via the recombination of CH_2_ and OH in an exoergic pathway of −113.5 kcal mol^−1^ (R6).

The formation of formaldehyde can be easily accessed via the barrierless reactions of CH_2_ with O(^3^P) and HCO with atomic hydrogen in exoergicities of 176.8 kcal mol^−1^ (R7) and 87.9 kcal mol^−1^ (R8), respectively. The alternative recombination pathway of HCO with atomic hydrogen can lead to hydroxymethylene (HCOH) in an overall exoergicity of 36.0 kcal mol^−1^ (R9); this product can also undergo a [1,2]H-shift via tunneling to formaldehyde with a first-order tunneling half-life of around 2 h^57,58^. Both reaction channels of HCO with OH and O(^1^D) insertion into HCOH can lead to the formation of HCOOH in the overall exoergicity of 107.9 kcal mol^−1^ (R10) and 224.2 kcal mol^−1^ (R11), respectively.

Interestingly, we identified the formation of the simplest geminal diol‒methanediol CH_2_(OH)_2_, whose TPD profile depicts a weak shoulder between 170 and 190 K at *m/z* = 48 in the CH_4_–O_2_ ice, 52 in the CD_4_–O_2_ ice and 52 in the CH_4_–^18^O_2_ ice, respectively (Fig. 2h)^54^. According to the KinBot network, CH_2_(OH)_2_ is suggested to form via the barrierless formation pathways including the hydrogen atom reaction with the hydroxymethoxy (HOCH_2_O) radical (R12: −104.8 kcal mol^−1^), the recombination of CH_2_OH with OH (R13: −97.2 kcal mol^−1^), or the hydrogen atom reaction with the dihydroxymethyl (CH(OH)_2_) radical (R14: −96.2 kcal mol^−1^). The CH(OH)_2_ radical can be accessed via the hydrogen tunneling in HOCH_2_O or barrierless OH radical reaction to the carbon of hydroxymethylene (HCOH) (R15: −82.9 kcal mol^−1^), while the HOCH_2_O radical can be generated via the association of CH_2_OH with O(^3^P) (R16: −94.7 kcal mol^−1^).

The formation evidence of the CH(OH)_2_ transient can be further supported by the identification of methanetriol (CH(OH)_3_) (Fig. 2g)^24^. Our TPD profiles peaking near 195 K between 180 and 210 K at *m/z* = 47 (CH(OH)_2_^+^), 50 (CD(OD)_2_^+^) and 51 (CH(^18^OH)_2_^+^) in the respective CH_4_‒O_2_, CD_4_‒O_2_ and CH_4_–^18^O_2_ ices agree nicely with those obtained through the dissociative fragmentation channels of the parent methanetriol cation (CH(OH)_3_^+^) in the higher-dose irradiated methanol‒molecular oxygen ice experiment. In addition, the TPD profiles at *m/z* = 31 (CH_3_O^+^), 34 (CD_3_O^+^) and 33 (CH_3_^18^O^+^) in the respective CH_4_‒O_2_, CD_4_‒O_2_ and CH_4_–^18^O_2_ ice support the identification of the methanetriol isomer hydroxyperoxymethanol (CH_2_(OH)O_2_H), whose fragmentation TPD profiles are in line with those reported earlier in the methanol‒molecular oxygen ice^24^. In addition, we scanned its PIE curve which clearly indicates the appearance energy of 10.95 ± 0.05 eV that matches nicely with that of 11.03 eV for CH(OH)_2_^+^ in the CH_3_OH–O_2_ experiment^24^. These findings strongly indicate the generation of CH_2_OH and CH(OH)_2_ radical transients in the irradiated CH_4_‒O_2_ ice as proposed by the molecular reaction network.

The PIE identification also evidently indicates the existence of CH_3_O and CH_3_OO radicals, which can be connected via the association of CH_3_O with O(^3^P). According to the KinBot network, CH_3_OOH can form through reaction of hydroperoxymethyl (CH_2_OOH) radical with atomic hydrogen (R17: −97.0 kcal mol^−1^), of methylperoxy (CH_3_OO) radical with atomic hydrogen (R18: −85.3 kcal mol^−1^), methyl (CH_3_) with hydroperoxyl (HOO) radical (R19: −67.1 kcal mol^−1^), or methoxy (CH_3_O) radical with hydroxyl (OH) radical (R20: −43.3 kcal mol^−1^). The hydroperoxymethyl (CH_2_OOH) radical can be accessed in a multistep reaction commencing with an O(^1^D) addition to the terminal oxygen atom of formaldehyde (H_2_CO) yielding the smallest Criegee intermediate carbonyl oxide (CH_2_OO) (R21: −56.4 kcal mol^−1^), which then adds atomic hydrogen to its terminal oxygen to form hydroperoxymethyl (CH_2_OOH) radical. Its isomeric form, the methylperoxy (CH_3_OO) radical, is proposed to form through the successive O(^3^P) addition to CH_3_ leading to CH_3_O (R22: −88.0 kcal mol^−1^) and CH_3_OO (R23: −60.2 kcal mol^−1^), respectively. The zwitterionic form of CH_3_OOH, *i.e*. CH_3_O(H)O, is suggested to form barrierlessly via the reactions of CH_2_O(H)O with H (R24: −101.7 kcal mol^−1^), CH_3_OH with O(^1^D) (R25: −47.4 kcal mol^−1^) or CH_3_ with OOH (R26: −23.3 kcal mol^−1^), in which CH_2_O(H)O can be linked to the barrierless addition of O(^1^D) to CH_2_OH (R27: −41.2 kcal mol^−1^). However, neither CH_3_O(H)O nor CH_2_O(H)O can initiate reactions leading to our products that are formed in the CH_4_ − O_2_ ice analogs.

**Fig. 5:**
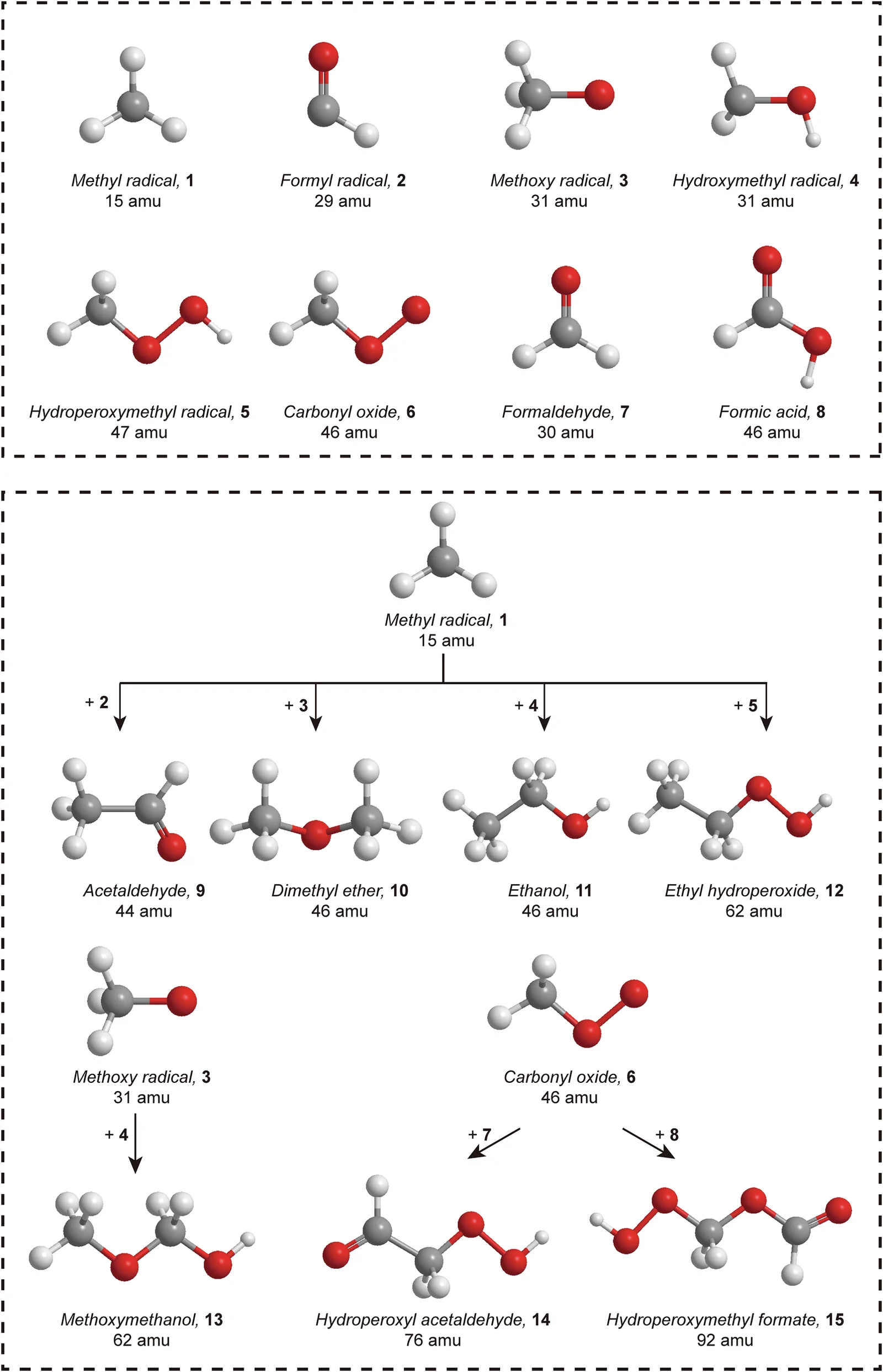
Proposed formation pathways from first-generation C1 to second-generation C2 products in irradiated CH_4_–O_2_ ices. The upper panel summarizes the primary radiolysis products of CH_4_–O_2_ ices, including C1 radicals—methyl (15 amu), formyl (29 amu), methoxy/hydroxymethyl (31 amu), and carbonyl oxide (46 amu), hydroperoxymethyl (47 amu)—as well as stable species such as formaldehyde (30 amu) and formic acid (46 amu). The lower panel presents the subsequent evolution toward C2 oxygen-bearing organics through radical–radical/molecular recombination processes, yielding acetaldehyde (44 amu), dimethyl ether and ethanol (46 amu), ethyl hydroperoxide and methoxymethanol (62 amu), hydroperoxyl acetaldehyde (76 amu), and hydroperoxymethyl formate (92 amu), respectively. Molecular masses (in amu) are indicated for each species, highlighting the stepwise growth of chemical complexity relevant to interstellar organic chemistry.

We can now correlate the identification of all C1 and C2 oxygen-bearing organic molecules with the proposed radical-radical reaction mechanism as inferred from the computational CH_4_–O_2_ reaction network (Fig. 5). Firstly, we provide evidence for a critical pool of highly reactive radicals such as CH_3_, HCO, CH_3_O, CH_2_OH, CH_2_OOH, and CH_2_OO, which is supported by the formation of CH_3_OH, CH_3_CHO, CH_3_OCH_2_OH, and CH_3_OOH. Secondly, closed-shell oxygenated COMs including CH_3_CHO, CH_3_OCH_3_, C_2_H_5_OH, C_2_H_5_OOH, and CH_3_OCH_2_OH are clearly identified, suggesting radical-radical association of CH_3_ with HCO, CH_3_O, CH_2_OH, CH_2_OOH, and CH_3_O with CH_2_OH respectively. In contrast, larger COMs (e.g., propanal and propylene oxide) generally require more complex precursors (C2/C3) or additional reaction steps, which are not accessible under the present experimental conditions. Thirdly, addition of carbonyl oxide (CH_2_OO) to H_2_CO might lead to 1,2,3-trioxolane followed by a diradical intermediate that eventually proceeds via a 1,4-H shift to the lowest-energy isomer of C_2_H_4_O_3_, *i*.*e*. hydroperoxyl acetaldehyde^59^. Similarly, addition of CH_2_OO with HCOOH can lead to the formation of the simplest hydroperoxide ester, hydroperoxymethyl formate (HOOCH_2_OCHO)^60,61^. Here, CH_2_OO might attack the O‒H moiety in HCOOH to form hydroperoxymethyl formate conformers, with its intramolecular hydrogen-bonded structure depicting the least energy form^60^.

While the automated search identifies several theoretically stable species (e.g., dioxirane, CH_3_O(H)O, and HCOH), their absence in the experimental spectra likely reflects rapid isomerization, low steady-state abundances, or detection limits rather than the exclusion of the corresponding pathways. For example, CH_3_O(H)O can readily isomerize to the more stable CH_3_OOH over a low barrier ( ~ 4.9 kcal mol^−1^), while species such as HCOH may undergo efficient conversion (e.g., via tunneling) to H_2_CO. Consequently, the non-detection of these intermediates is more indicative of their high reactivity or minor branching ratios than evidence against the proposed reaction network.

Raw ion intensities in the mass spectra do not directly reflect molecular abundances due to species-dependent ionization cross sections and fragmentation. To enable a more meaningful comparison, signals for species with available photoionization cross sections (PICS) were normalized accordingly. The resulting values (Supplementary Table 3) provide a semi-quantitative estimate of relative product yields and allow distinction between major and minor species within the reaction network.

## Conclusions

Comets contain key classes of molecules such as sugars and polyols of prebiotic relevance that are related to biological processes on Earth, and have hence been proposed as potential indicators for the presence of biological processes in the search for extraterrestrial life^17,22,62,63^. Cometary nuclei are considered to most closely reflect the composition of the building blocks of our solar system. An unexpectedly high abundance of gaseous molecular oxygen was measured in situ in the coma of comet 67P/Churyumov–Gerasimenko (hereafter 67P)^64,65^. The Neutral Mass Spectrometer (NMS) measurements from the Giotto flyby yielded data consistent with O_2_ in the coma of comet 1P/Halley with concentrations similar to those detected in the coma of 67P^66^, implying that cometary O_2_ may be a common attribute of the otherwise dynamically different Jupiter-family (e.g. 67P) and Oort Cloud (e.g. 1P/Halley) comets. The other highly volatile species, CH_4_, has been observed in numerous comets including both 67P and 1P/Halley, while its high abundance indicates that 67P has never experienced temperatures above tens of Kelvins since the comet formation^62,67^.

The Rosetta orbiter and its lander Philae successfully detected a variety of volatile organic compounds on 67P including aldehydes and alcohols^62,64,68^. However, the origin of these COMs in cometary ices still remains debated. At least some of the organics in 67P have been regarded as being of prestellar origin^69^, with subsequent ice processing considered either trivial^63^ or limited to amorphous-to-crystalline ice-phase transitions and re-trapping of volatiles^70^. In the non-equilibrium reactions of CH_4_–O_2_ ices, we identified several oxygen-bearing organic molecules that have not yet been detected in extraterrestrial environments, including the methylperoxy radical (CH_3_OO), methyl hydroperoxide (CH_3_OOH), methanediol (CH_2_(OH)_2_), ethyl hydroperoxide (C_2_H_5_OOH), methanetriol (CH(OH)_3_), hydroperoxyl acetaldehyde (HOOCH_2_CHO), and hydroperoxymethyl formate (HOOCH_2_OCHO). Most of these molecules, as confirmed by astrochemical experiments and reaction network generated by machine learning techniques, are also nascent products involving Criegee intermediates in the atmospheric chemistry^52,60^. They might be viable candidates for astronomical observations with instruments like the Atacama Large Millimeter/submillimeter Array (ALMA), or even the James Webb Space Telescope (JWST), which offers high sensitivity and spectral resolution in the fingerprint region of oxygen-bearing organic molecules, *i*.*e*. 5–10 μm (2000 cm^−1^–1000 cm^−1^).

We have untangled the molecular complexity in the electron-irradiated CH_4_–O_2_ ice analogs, revealing a rich inventory of oxygen-bearing organic molecules including aldehydes (formaldehyde, acetaldehyde), alcohols (methanol, ethanol), and polyols like methanediol and methanetriol, peroxides (hydrogen peroxide, methyl hydroperoxide, ethyl hydroperoxide, and keto-hydroperoxides including hydroperoxyl acetaldehyde and hydroperoxymethyl formate), ethers (dimethyl ether and methoxymethanol), formic acid and even radicals (methoxy radical and methylperoxy radical) under extraterrestrial-like conditions. In contrast to traditional spectroscopic and spectrometric methods, we present a universal approach to discriminate structural isomers based on the tunable synchrotron vacuum ultraviolet photoionization reflectron time-of-flight mass spectrometry (SVUV-PI-ReToF-MS) during temperature-programmed desorption (TPD). The experimental findings are further consolidated by the automated potential-energy- surface search, which exhibits a complicated radical-induced reaction network in the simulated ice analogs. Our results not only demonstrate the explicit formation of COMs that elude astronomical detection so far, but also provide detailed insight into the molecular reaction network that can advance the understanding of astrochemical modeling at the molecular and quantum-chemical levels. Therefore, our investigation of astrophysical ice analogs composed of simple species like methane and molecular oxygen can help interpret the complicated, but concerted molecular inventory and underlying reaction mechanism, further highlighting the potential application to bridging the discrepancy between astronomical observation and astrochemical modeling. We anticipate that the combined approach will contribute to our future understanding of extraterrestrial ice analogs by providing hypotheses for molecular species and elementary reaction pathways in fields as diverse as prebiotic chemistry and astrochemistry.

## Methods

### Experimental

To investigate the formation of prebiotic molecules under astrochemical conditions, we conducted a series of experiments at the Shanghai-Hawaii-Hefei Advanced Research Center (SHHARC) for Astrochemistry (Supplementary Table 4), utilizing the VUV beamline BL03U at the National Synchrotron Radiation Laboratory^71^. The SHHARC features a stainless steel ultra-high vacuum chamber, maintained at pressures around 10^−11^ Torr, and is equipped with contamination-free turbomolecular pumps (Osaka Vacuum, TG1300M, TG420M), backed by a dry scroll pump (Leybold, Ecodry 40 Plus). Within the chamber, a polished silver substrate was mounted on an oxygen-free high thermal conductivity copper cold finger, capable of both vertical and horizontal movement via an ultrahigh-vacuum-compatible bellows (McAllister, BLT106) and a differentially pumped rotary platform (Thermionics Vacuum Products, RNN-600/FA/MCO). The cold finger, interfaced with a two-stage closed-cycle helium refrigerator (Sumitomo Heavy Industries, RDK-415E), achieved temperatures as low as 4.7 ± 0.1 K, monitored by a cryogenic temperature sensor (Lakeshore, DT-470).

For the deposition process, the silver substrate was cooled to 4.7 K and positioned for deposition. Methane (99.999%) and oxygen (99.999%) were thoroughly mixed in a 1:1 ratio in a premixing chamber and uniformly deposited onto the substrate through a capillary array at 2×10^−8^ Torr. The ice thickness, monitored in situ by laser interferometry using a He-Ne laser (632.8 nm), was determined to be 620 ± 50 nm (Supplementary Fig. 4), using refractive indices *n*_O2_ = 1.25 and *n*_CH4_ = 1.34 ± 0.04^72,73^. The substrate was then positioned for electron irradiation at a 70° angle relative to the ice surface, and irradiated with 5 keV electrons (SPECS GmbH, EQ 22/35 electron source) at 50 nA for 30 min. To correlate our experimental conditions with astrophysical environments, we estimated the total irradiation dose delivered to the ice. The electron fluence utilized here corresponds to an evolutionary timescale of approximately 10^7^ years within a dense molecular cloud, assuming a typical cosmic-ray ionization rate. This duration encompasses the characteristic lifetime of such clouds, indicating that the reaction pathways and products identified in this study are representative of the long-term chemical processing of interstellar grain mantles. The ice thickness exceeded the average electron penetration depth of 292 ± 29 nm (Supplementary Fig. 5 and Supplementary Table 5), calculated using Monte Carlo simulations in CASINO 2.42^74^.

After irradiation, the ice mixtures were rotated to the temperature-programmed desorption (TPD) position and annealed from 5 to 300 K at a rate of 1 K min^−1^. Sublimating molecules were monitored using synchrotron vacuum ultraviolet photoionization reflectron time-of-flight mass spectrometry (SVUV-PI-ReToF-MS). The blank experiment, *i.e*., the non-irradiated experiment, followed the same procedure. Ionized species were mass analyzed using a ReToF mass spectrometer (Jordan TOF Products, Inc.), operating with 2.5 μs extraction pulses generated by a pulse delay generator (Quantum Composers, 9528) at a 15 kHz repetition rate. Signals were amplified, discriminated using an F-100T amplifier-discriminator (Advanced Research Instruments Corporation), and recorded using a multi-channel scalar (FAST ComTec, P7889). During the TPD phase, SVUV was scanned in 0.05 eV increments within a selected SVUV energy range, and photon energy and temperature-dependent mass spectra were recorded. PIE curves for all detected molecules were generated by normalizing to the TPD profiles at 11.50 eV and photon fluxes^23^.

### Computational

We employed the KinBot program^28,29^ for automated, efficient, and comprehensive exploration of reaction networks. KinBot excels at initiating searches from single-molecule reactants: starting from a given initial single-molecule species, it generates products of elementary reactions and corresponding initial guesses of transition states via template matching, followed by molecular optimization and reaction path verification. This process is iteratively repeated to obtain a complete reaction network. The automated exploration was restricted to C1 species to maintain computational tractability. Extending the network to C2 chemistry would lead to a rapid increase in the number of possible isomers and transition states due to higher degrees of freedom. In addition, the high-level electronic structure refinements employed here (CCSD(T), scaling as *O*(*N*^7^)) would render a comprehensive C2 exploration computationally prohibitive. We conducted reaction path searches with H_2_O_2_, H_2_CO, HCOOH, CH_2_OH, CH_3_OO, CH_2_OOH, CH_3_OOH, and CH_2_(OH)_2_ as initial reactants, respectively (Supplementary Fig. 3 and Supplementary Table 6). To accelerate the search process, we utilized the UMA neural network potential energy surface model^27^. Benefiting from its extensive data foundation and advanced architectural design, the UMA model performs well in handling chemical reactions, particularly those involving free radicals. Based on the reaction paths generated by KinBot, we supplemented the reaction network with human expertise, accounting for approximately 10% of the total.

Subsequently, all reaction paths were verified using high-precision quantum chemical calculations. For reactants, products, intermediates, and transition states provided by KinBot, geometry optimizations and vibrational frequency analyses were first performed at the ωB97X-D/def2-TZVP level^75^, followed by zero-point vibrational energy (ZPE) corrections. For exceptional cases where convergence could not be achieved at this level, MP2/6-31G(d)^76^ calculations were used instead. All stationary points were verified to ensure that local minima had no imaginary frequencies and transition states had exactly one imaginary frequency. Each transition state was further validated through intrinsic reaction coordinate (IRC) calculations to confirm the connectivity between the corresponding reactants and products. For barrierless processes, relaxed scans were conducted to ensure smooth reaction profiles without hidden transition states.

Optimized structures were used to calculate single-point energies using the coupled cluster method at CCSD(T)/aug-cc-pVnZ (n = D, T) levels^77^ with extrapolation to the complete basis set (CBS) limit^78^. ZPE corrections from DFT calculations were scaled with factors of 0.975 for ωB97X-D/def2-TZVP and 0.967 for MP2/6-31G(d)^79^. T1 diagnostics were employed to check the multi-reference character, ensuring that the energies can be adequately described by the coupled cluster method. All quantum chemical calculations were carried out with the Gaussian 16 computational package^80^.

## Supplementary information

Supplementary Information

## Data Availability

The data supporting the findings of this study are available from the corresponding authors upon reasonable request.
