# Supplementary material for: Experimental astrochemistry and machine learning unravel the formation of oxygen-bearing organic molecules in extraterrestrial ices
Source: Commun Chem. 2026 Jul 14;9:359. doi: 10.1038/s42004-026-02107-2 (PMC13754802; doi:10.1038/s42004-026-02107-2)
Supplement: Supplementary file 1 [file 42004_2026_2107_MOESM1_ESM.pdf]

Supplementary Information for

Experimental astrochemistry and machine learning unravel the formation of oxygen-bearing organic molecules in extraterrestrial ices

Xilin Bai[1†], Sixuan Mi[2†], Qi'ang Gong[1], Jinghui Lu[5], Jiuzhong Yang[5], Yang Pan[5], Xiaohu Li[3], Zhenrong Sun[1], Tong Zhu[2*], Ralf I. Kaiser[6*], Tao Yang[1,3,4*]

[1] State Key Laboratory of Precision Spectroscopy, East China Normal University, Shanghai 200062, China

[2] Shanghai Engineering Research Center of Molecular Therapeutics and New Drug Development, School of Chemistry and Molecular Engineering, East China Normal University, Shanghai 200062, China

[3] State Key Laboratory of Radio Astronomy and Technology, Xinjiang Astronomical Observatory, Chinese Academy of Sciences, 150 Science 1-Street, Urumqi, Xinjiang, 830011, China

[4] Collaborative Innovation Center of Extreme Optics, Shanxi University, Taiyuan, Shanxi 030006, China

[5] National Synchrotron Radiation Laboratory, University of Science and Technology of China, Hefei, Anhui 230029, China

[6] Department of Chemistry, University of Hawaii at Manoa, Honolulu, HI 96822, USA

Correspondence to: tyang@lps.ecnu.edu.cn (T.Y.), tzhu@lps.ecnu.edu.cn (T.Z.), ralfk@hawaii.edu (R.I.K.)

This PDF file includes:

Supplementary Methods
Supplementary Figures 1 to 5
Supplementary Tables 1 to 6
Supplementary References

Supplementary Methods

1. Ice thickness determination

The refractive indices of solid methane (n_{CH4} = 1.34)[1] and solid oxygen (n_{O2} = 1.25)[2] were employed in Equation S1 to determine the ice thickness (d) based on the interference pattern of a laser (λ = 632.8 nm) at an incidence angle of θ = 4°:

$$d = \frac{m\lambda}{2\sqrt{n_{mix}^2 - sin^2\theta}} \quad \text{(S1)}$$

Here, the interference pattern fringes (m) were determined using plots such as Supplementary Fig. 4, illustrating the interference pattern during ice deposition. Having observed 2.5 interference fringes, the thickness of CH_4 and O_2 ice mixture was calculated to be 620 ± 50 nm.

2. Calibration Experiments

The calibration experiments were designed to determine the molecular ratio of methane (CH_4) to oxygen (O_2) in the mixed ice. Initially, laser interferometry was used to precisely measure the thicknesses of pure CH_4 and O_2 ices deposited on a silver substrate, yielding 236 ± 20 nm and 254 ± 20 nm for single fringe CH_4 and O_2 ice, respectively. These samples were subsequently subjected to temperature-programmed desorption (TPD), with a linear heating rate of 1 K min^{-1}. The desorbed species were monitored using a quadrupole mass spectrometer (QMS), which detected both parent and fragment ions. The QMS was operated under standard conditions: an electron current of 2 mA, electron energy of 70 eV, and a photomultiplier voltage of 1500 V. Methane was monitored via m/z = 16 (CH_4^+) and m/z = 15 (CH_3^+), while oxygen was tracked through m/z = 32 (O_2^+) and m/z = 16 (O^+). Since the integrated ion signal is proportional to the number of desorbed molecules[3], the CH_4:O_2 molecular ratio for a given thickness could be accurately established. Subsequent blank experiments confirmed the CH_4:O_2 ratio in the mixed ice. Methane and oxygen were pre-mixed in a 1:1 pressure ratio in a premixing chamber and co-deposited onto the substrate through a capillary array at a base pressure of 2×10^{-8} Torr. The resulting mixed ice had a thickness of 620 ± 50 nm, as determined via interferometry. TPD-QMS measurements of CH_4 (CH_4^+ and CH_3^+) and O_2 (O_2^+ and O^+) were compared to those of the pure ices. Based on this comparison, the methane-to-oxygen ratio in the mixed ice was determined to be 1:1.1 ± 0.1.

Supplementary Figures

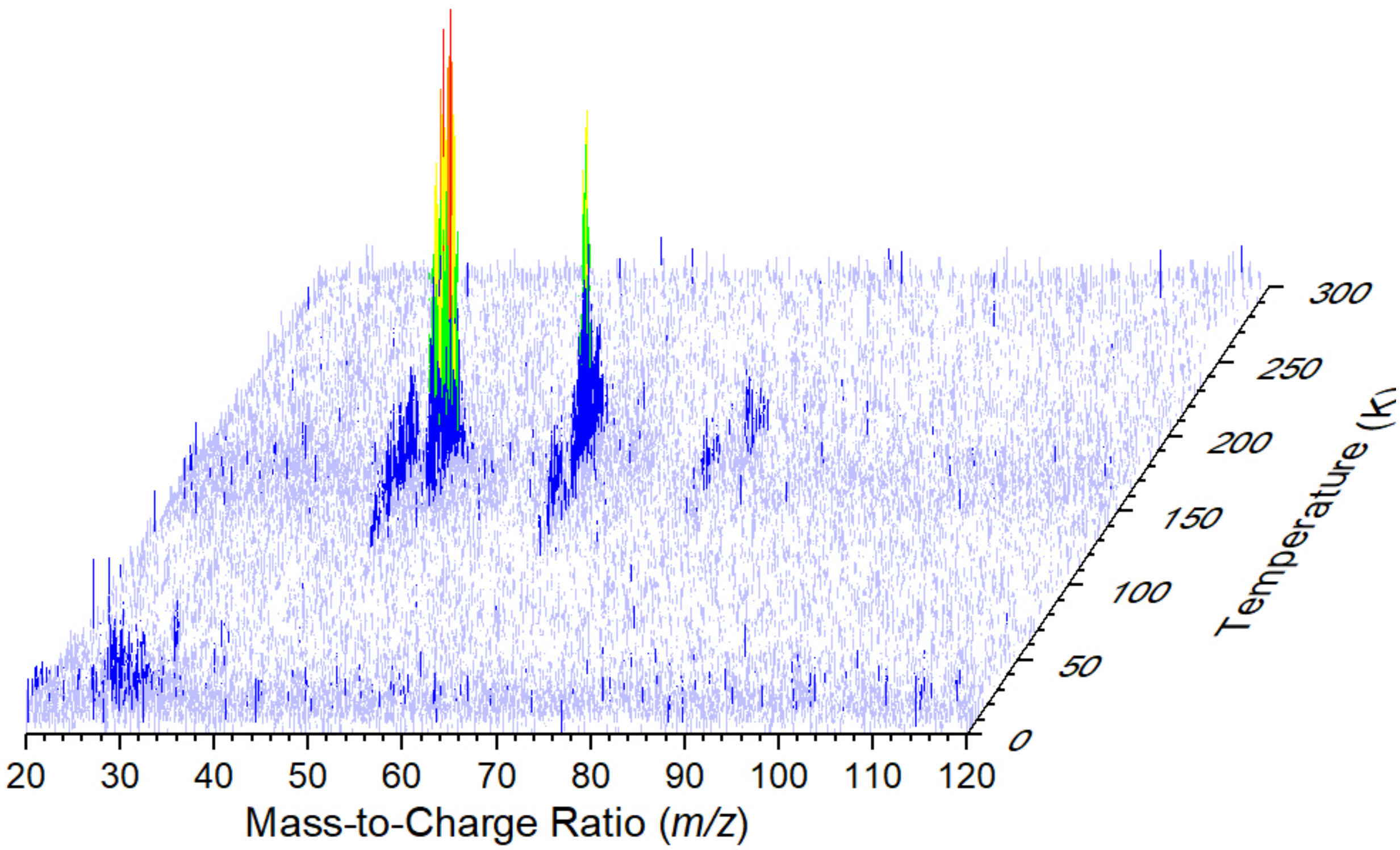

Supplementary Figure 1. SVUV-PI-ReToF-MS data during the TPD phase of irradiated CH_4–O_2 ice. Data were recorded at the photon energy of 10.49 eV. The irradiated ices were prepared under the exposure of 5 keV electrons of 50 nA for 30 minutes.

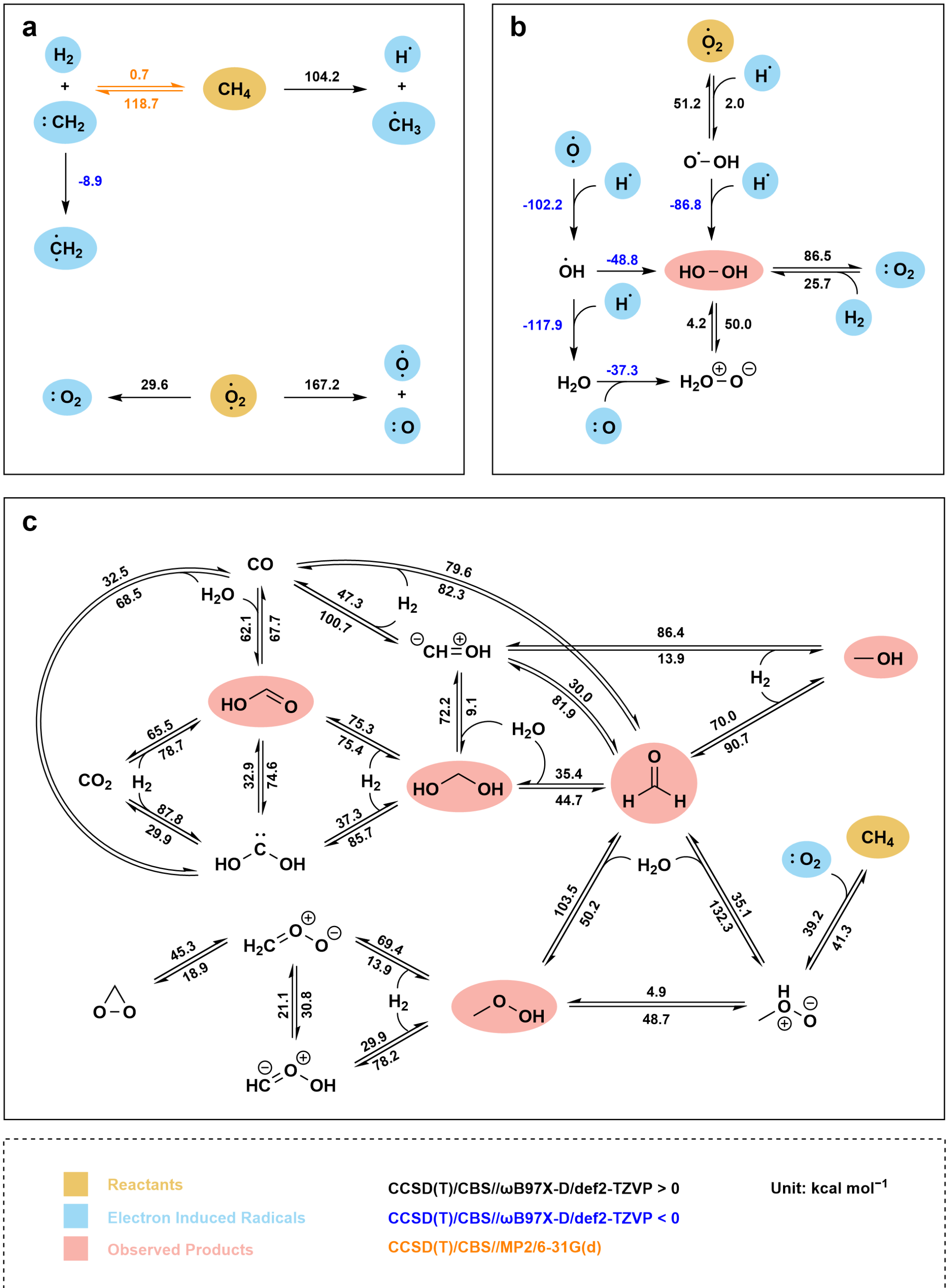

Supplementary Figure 2. Calculated reaction network. (**a**) Electron-induced activation of reactants CH_4 and O_2 leading to the generation of primary species. (**b**) The formation of H_2O_2 with electron induced radical species. (**c**) The formation of C1 compounds without electron induced radical species.

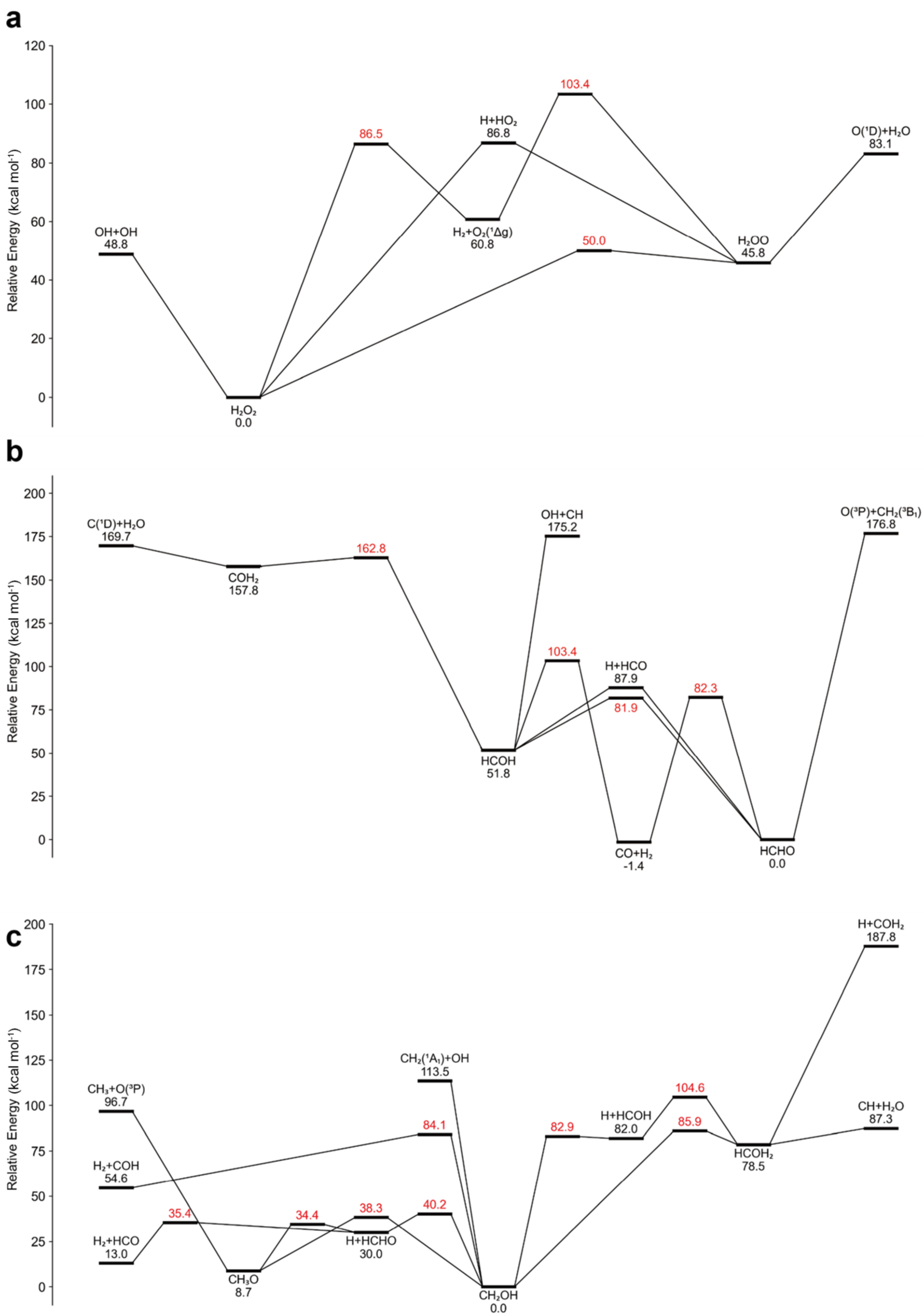
a
Relative Energy (kcal mol^{-1})
OH+OH
48.8
H_2O_2
0.0
86.5
H+HO_2
86.8
H_2+O_2($^1\Delta$g)
60.8
103.4
50.0
H_2OO
45.8
O(^{1}D)+H_2O
83.1
b
Relative Energy (kcal mol^{-1})
C(^{1}D)+H_2O
169.7
COH_2
157.8
162.8
HCOH
51.8
OH+CH
175.2
103.4
H+HCO
87.9
81.9
CO+H_2
-1.4
82.3
HCHO
0.0
O(^{3}P)+CH_2($^3B_1$)
176.8
c
Relative Energy (kcal mol^{-1})
CH_3+O(^{3}P)
96.7
H_2+COH
54.6
H_2+HCO
13.0
35.4
CH_3O
8.7
34.4
38.3
H+HCHO
30.0
40.2
84.1
CH_2($^1A_1$)+OH
113.5
CH_2OH
0.0
82.9
H+HCOH
82.0
104.6
85.9
$HCOH_2$
78.5
H+COH_2
187.8
CH+H_2O
87.3

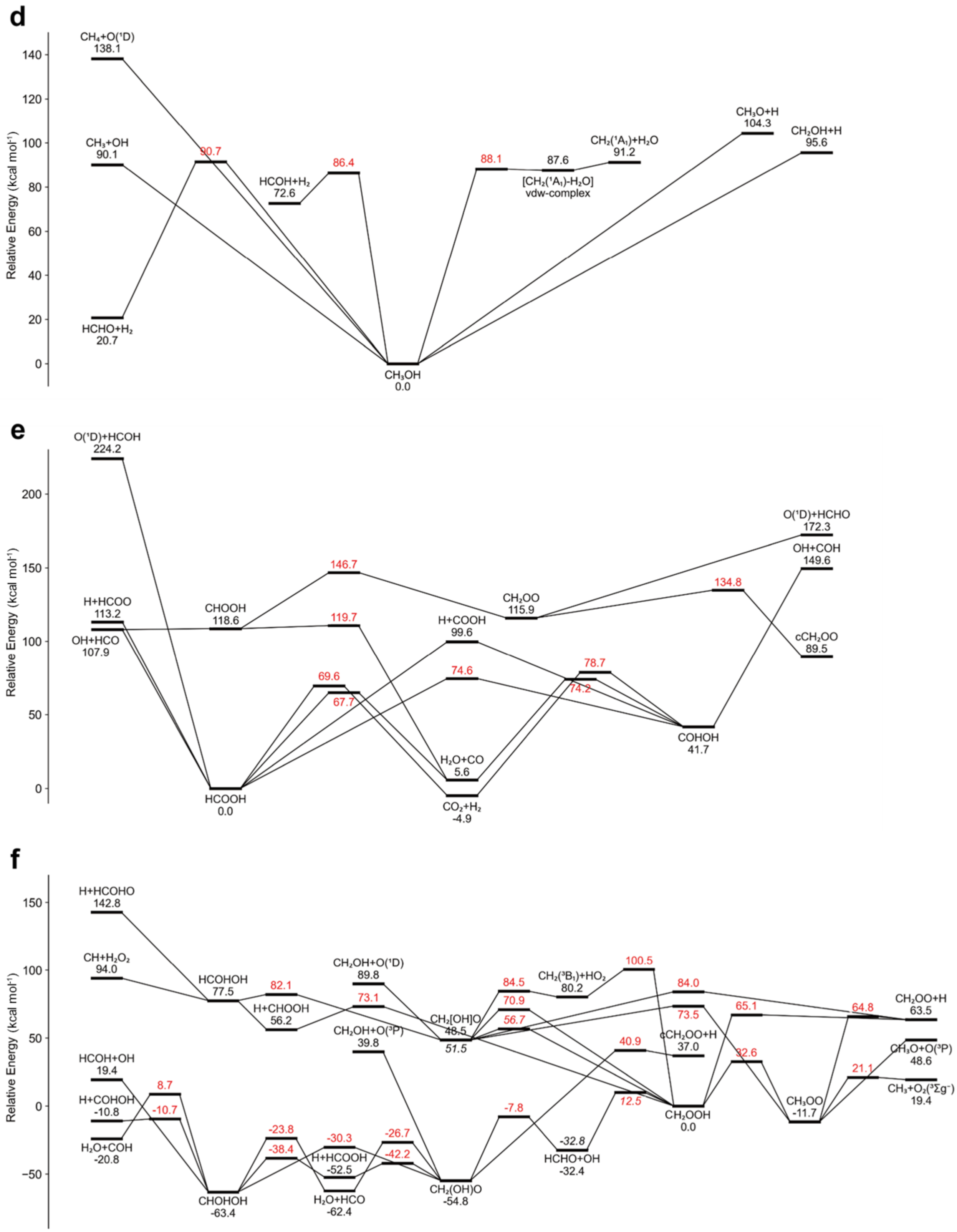

d
Relative Energy (kcal mol-1)
CH4+O(1D)
138.1
CH3+OH
90.1
HCHO+H2
20.7
90.7
HCOH+H2
72.6
86.4
CH3OH
0.0
88.1
87.6
[CH2(1A1)-H2O]
vdw-complex
CH2(1A1)+H2O
91.2
CH3O+H
104.3
CH2OH+H
95.6
e
Relative Energy (kcal mol-1)
O(1D)+HCOH
224.2
H+HCOO
113.2
OH+HCO
107.9
HCOOH
0.0
CHOOH
118.6
146.7
119.7
69.6
67.7
H+COOH
99.6
74.6
H2O+CO
5.6
CO2+H2
-4.9
CH2OO
115.9
78.7
74.2
COHOH
41.7
134.8
O(1D)+HCHO
172.3
OH+COH
149.6
cCH2OO
89.5
f
Relative Energy (kcal mol-1)
H+HCOHO
142.8
CH+H2O2
94.0
HCOH+OH
19.4
H+COHOH
-10.8
H2O+COH
-20.8
8.7
-10.7
HCOHOH
77.5
CHOHOH
-63.4
82.1
H+CHOOH
56.2
-23.8
-38.4
CH2OH+O(1D)
89.8
73.1
CH2OH+O(3P)
39.8
-30.3
H+HCOOH
-52.5
H2O+HCO
-62.4
-26.7
-42.2
CH2[OH]O
48.5
51.5
CH2(OH)O
-54.8
84.5
70.9
56.7
-7.8
CH2(3B1)+HO2
80.2
-32.8
HCHO+OH
-32.4
100.5
40.9
12.5
84.0
73.5
cCH2OO+H
37.0
CH2OOH
0.0
65.1
32.6
CH3OO
-11.7
64.8
21.1
CH2OO+H
63.5
CH3O+O(3P)
48.6
CH3+O2(3Σg-)
19.4

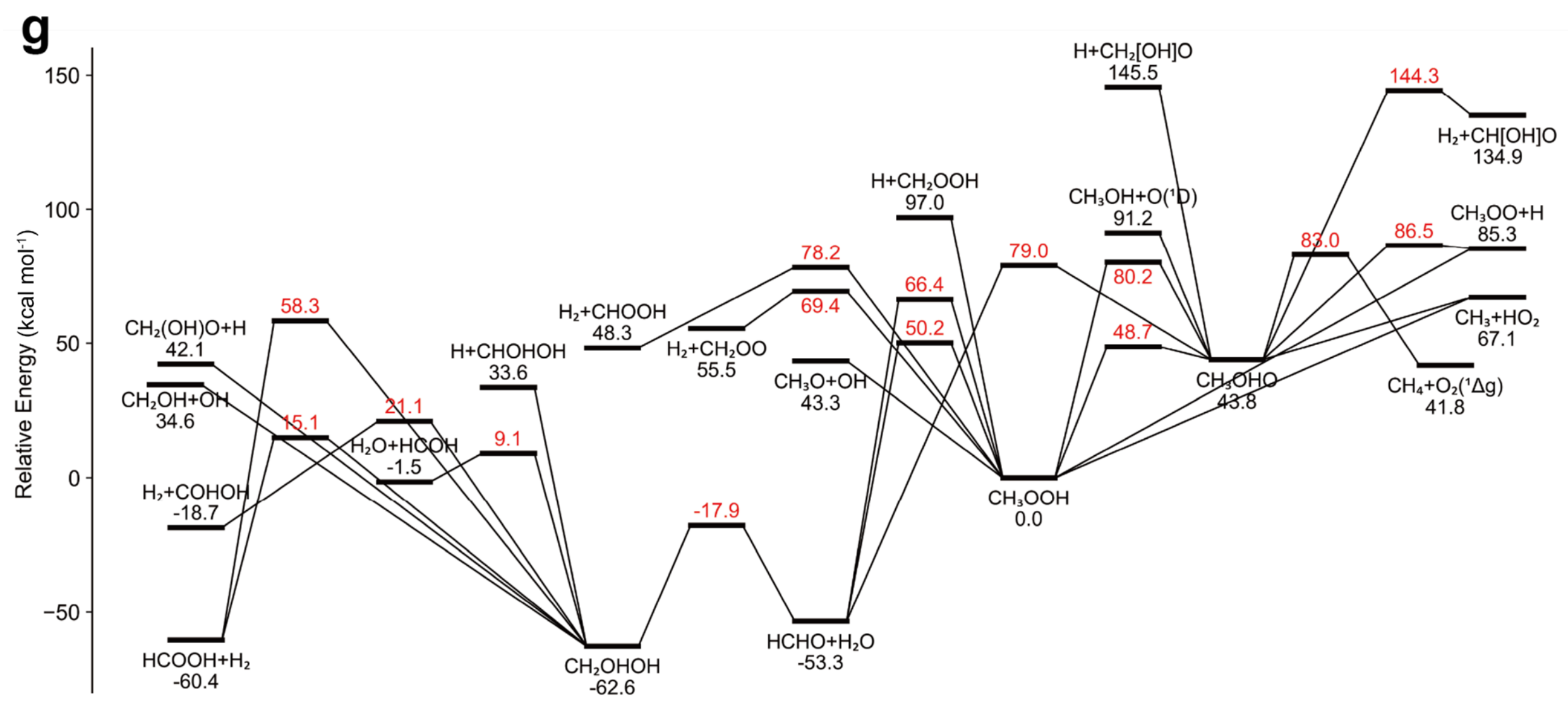

Supplementary Figure 3. Calculated potential energy surfaces for systems of (a) H_2O_2, (b) CH_2O, (c) CH_3O, (d) CH_4O, (e) CH_2O_2, (f) CH_3O_2, and (g) CH_4O_2 using Kinbot, respectively. Relative energies including zero-point vibrational correction are labeled numerically, which are refined by single-point calculations at the CCSD(T)/CBS level. Numbers in black correspond to intermediates, while numbers in red correspond to transition states. Numbers in roman fonts denote those calculated at the level of ωB97X-D/def2-TZVP, while numbers in italic fonts denote those at the level of MP2/6-31G(d). All the energies, geometries, coordinates and frequencies of relevant species calculated in the reaction network exploration are complied in Supplementary Table 6.

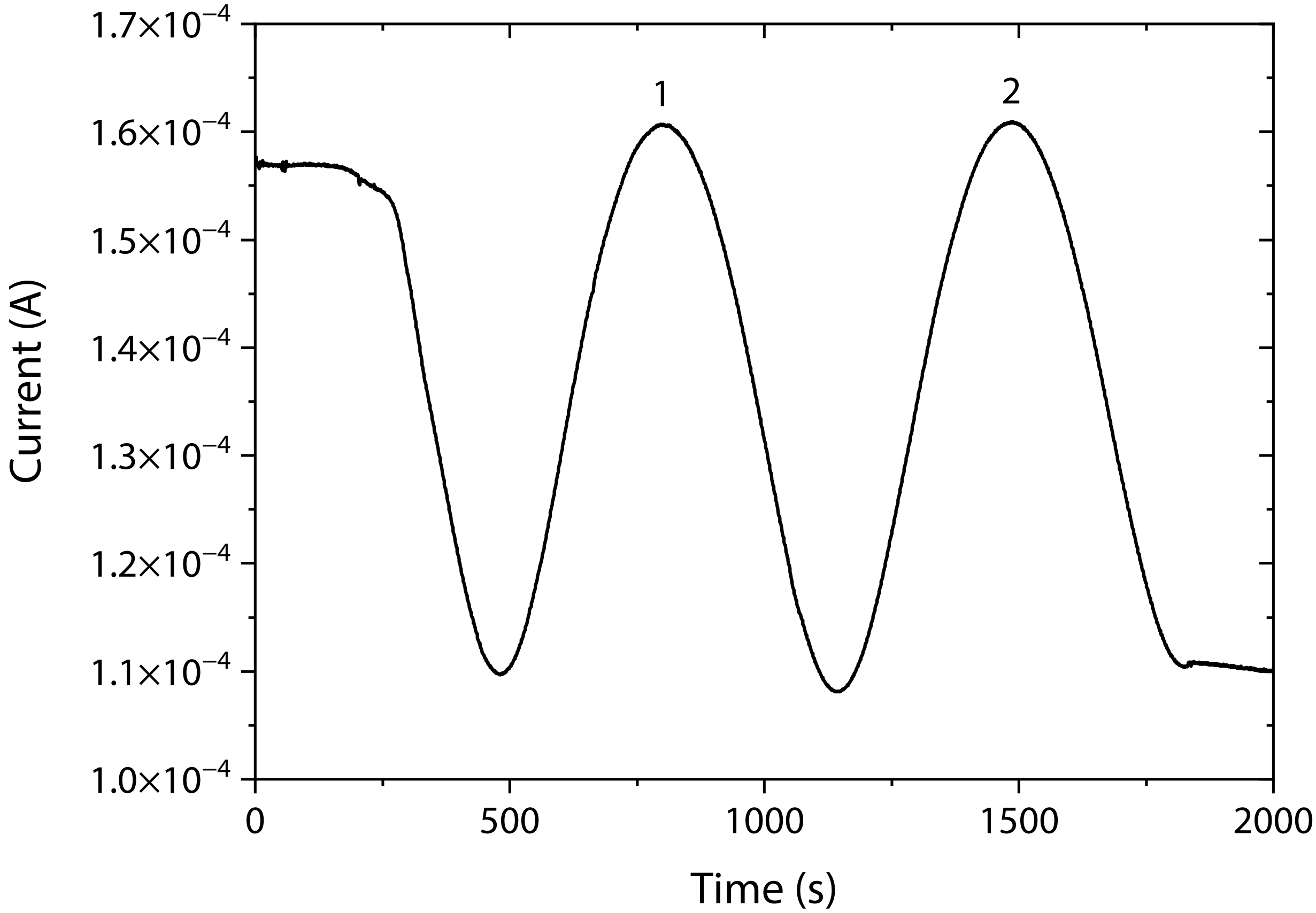

Supplementary Figure 4. Real-time laser-interference monitoring ice growth. Interference pattern observed for a 632.8 nm laser during CH_4–O_2 ice mixture deposition at an incidence angle of 4°. Integer numbers of fringes are indicated above signal maxima, and at the end of the deposition process, 2.5 fringes had accumulated.

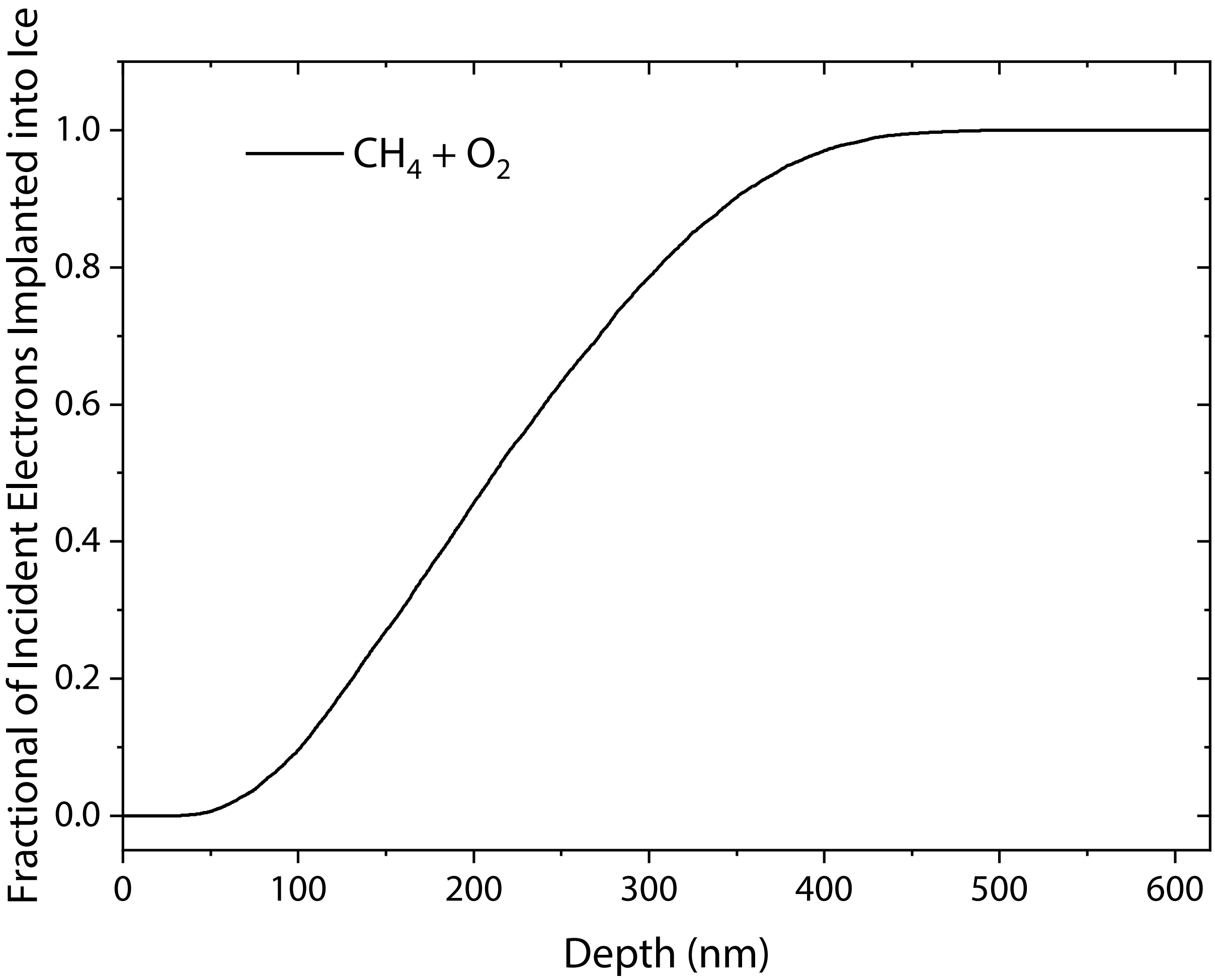

Supplementary Figure 5. Penetration depths simulated with CASINO[4]. The maximum penetration depths of 5 keV electrons in the CH_4–O_2 ice.

Supplementary Tables

Supplementary Table 1. Molecular formula, mass-to-charge ratios (*m/z*), and ionization energy (IE) of the products identified in the irradiated methane–oxygen isotopologue ices (CH_4–O_2, CD_4–O_2, and CH_4–$^{18}O_2$) during the TPD studies using ReToF mass spectroscopy.

CH_4–O_2		CD_4–O_2		CH_4–$^{18}O_2$		Product	Name	IE (eV)	IE Ref.	PIE Ref.
Formula	*m/z*	Formula	*m/z*	Formula	*m/z*					
CH_2O	30	CD_2O	32	$CH_2{}^{18}O$	32	H_2CO	formaldehyde	10.88 ± 0.01	5	5
CH_3O	31	CD_3O	34	$CH_3{}^{18}O$	33	CH_3O	methoxy radical	10.701 ± 0.005	6	6
CH_4O	32	CD_4O	36	$CH_4{}^{18}O$	34	CH_3OH	methanol	10.84 ± 0.01	7	7
H_2O_2	34	D_2O_2	36	$H_2{}^{18}O_2$	38	HOOH	hydrogen peroxide	10.631 ± 0.007	5	5
C_2H_4O	44	C_2D_4O	48	$C_2H_4{}^{18}O$	46	CH_3CHO	acetaldehyde	10.229 ± 0.0007	8	8
C_2H_6O	46	C_2D_6O	52	$C_2H_6{}^{18}O$	48	CH_3CH_2OH	ethanol	10.48 ± 0.07	7	7
C_2H_6O	46	C_2D_6O	52	$C_2H_6{}^{18}O$	48	CH_3OCH_3	dimethyl ether	10.025 ± 0.025	7	7
CH_2O_2	46	CD_2O_2	48	$CH_2{}^{18}O_2$	50	HCOOH	formic acid	11.33 ± 0.01	7	7
CH_3O_2	47	CD_3O_2	50	$CH_3{}^{18}O_2$	51	CH_3OO	methylperoxy radical	10.33 ± 0.05	9	9
CH_4O_2	48	CD_4O_2	52	$CH_4{}^{18}O_2$	52	CH_3OOH	methyl peroxide	9.77 ± 0.04	10	10
CH_4O_2	48	CD_4O_2	52	$CH_4{}^{18}O_2$	52	$CH_2(OH)_2$	methanediol	10.73 ± 0.01	11	11
$C_2H_6O_2$	62	$C_2D_6O_2$	68	$C_2H_6{}^{18}O_2$	66	CH_3OCH_2OH	methoxymethanol	10.25 ± 0.05	12	12
$C_2H_6O_2$	62	$C_2D_6O_2$	68	$C_2H_6{}^{18}O_2$	66	C_2H_5OOH	ethyl hydroperoxide	9.70 ± 0.05	13	13
CH_4O_3	64	CD_4O_3	68	$CH_4{}^{18}O_3$	70	$CH(OH)_3$	methanetriol	10.97 ± 0.04	14	N.A.
$C_2H_4O_3$	76	$C_2D_4O_3$	80	$C_2H_4{}^{18}O_3$	82	$HOOCH_2CHO$	hydroperoxyl acetaldehyde	9.85 ± 0.05	15	15
$C_2H_4O_4$	92	$C_2D_4O_4$	96	$C_2H_4{}^{18}O_4$	100	$HOOCH_2OCHO$	hydroperoxymethyl formate	10.65 ± 0.05	16	16

Supplementary Table 2. Energies, geometries, coordinates and frequencies of species $CH_3CH(OH)_2$ calculated in adiabatic ionization potential.

No.	Name	Energies (a.u.)	Atom	X (Å)	Y (Å)	Z (Å)	*v* (cm^{-1})	T1 diag.
**0**	$CH_3CH(OH)_2$_neutral	ZPE(ωB97X-D/def2-TZVP)=0.0856885<br>E(ωB97X-D/def2-TZVP)=−230.2971464<br>E(UCCSD(T)/aug-cc-pVDZ)=−229.7517734<br>E(UCCSD(T)/aug-cc-pVTZ)=−229.9584214	C<br>H<br>H<br>H<br>C<br>H<br>O<br>H<br>O<br>H	1.399480<br>1.452466<br>1.926890<br>1.884825<br>−0.046072<br>−0.118705<br>−0.673074<br>−1.589424<br>−0.708367<br>−0.624975	−0.036220<br>−0.027595<br>0.831919<br>−0.942322<br>−0.002837<br>−0.003452<br>−1.141398<br>−1.134496<br>1.172753<br>1.259444	−0.082747<br>−1.173059<br>0.307245<br>0.275123<br>0.359385<br>1.450522<br>−0.175553<br>0.106410<br>−0.029883<br>−0.982577	239.9, 323.8, 376.9<br>403.2, 496.8, 585.9<br>876.6, 960.6, 1054.4<br>1101.8, 1157.1, 1286.5<br>1313.5, 1401.8, 1424.8<br>1473.3, 1489.1, 1500.4<br>3050.9, 3056.9, 3136.1<br>3156.7, 3860.6, 3885.4	0.0123
**1**	$CH_3CH(OH)_2$_cation	ZPE(ωB97X-D/def2-TZVP)=0.0846417<br>E(ωB97X-D/def2-TZVP)=−229.91516<br>E(UCCSD(T)/aug-cc-pVDZ)=−229.3679923<br>E(UCCSD(T)/aug-cc-pVTZ)=−229.5691634	C<br>H<br>H<br>H<br>C<br>H<br>O<br>H<br>O<br>H	1.453326<br>1.454281<br>2.008748<br>1.946631<br>0.067954<br>−0.022185<br>−0.777758<br>−1.576267<br>−0.768419<br>−0.569468	−0.048952<br>−0.020061<br>0.800046<br>−0.962593<br>−0.023409<br>−0.071403<br>−0.969066<br>−1.158408<br>1.041994<br>1.263162	−0.115067<br>−1.205055<br>0.281772<br>0.214658<br>0.425070<br>1.506497<br>−0.231225<br>0.295443<br>−0.019275<br>−0.949338	234.5, 374.2, 433.7<br>515.2, 571.3, 598.6<br>717.9, 885.2, 1033.7<br>1056.2, 1150.8, 1201.1<br>1288.5, 1343.7, 1421.6<br>1461.4, 1476.7, 1493.0<br>3062.2, 3149.1, 3156.5<br>3172.1, 3662.4, 3693.9	0.0167

E(CCSD(T)/CBS) = [E(UCCSD(T)/aug-cc-pVTZ) − $(2.5^3/3.5^3)$ E(UCCSD(T)/aug-cc-pVDZ)] / [1 − $(2.5^3/3.5^3)$] + 0.975 ZPE(ωB97X-D/def2-TZVP)

AIP[$CH_3CH(OH)_2$] = E(CCSD(T)/CBS)[$CH_3CH(OH)_2$_cation] – E(CCSD(T)/CBS)[$CH_3CH(OH)_2$_neutral] = 10.65 eV

Supplementary Table 3. Photoionization cross section and relative intensity for species analyzed in this work.

Species	Isomer	Cross sections/Mb (10.49 eV)	Cross sections/Mb (11.50 eV)	Total counts	Relative intensities
H_2CO	Formaldehyde	4.94	10.13[17]	20071	1.00
CH_3CHO	Acetaldehyde	7.38	9.28[18]	2257	0.12
CH_3OH	Methanol	0	9.40[19]	10079	0.54
C_2H_5OH	Ethanol	0.52	5.24[20]	2232	0.21
HOOH	Hydrogen peroxide	0	1.84[21]	2806	0.77
CH_3OOH	Methyl peroxide	2.84	9.35[22]	10593	0.57
C_2H_5OOH	Ethyl hydroperoxide	6.04	8.94[22]	608	0.03
CH_3OCH_3	Dimethyl ether	8.16	10.04[20]	1415	0.07
HCOOH	Formic acid	0	5.04[20]	2527	0.25

Supplementary Table 4. List of experiments.

#	Precursors	Electron dose	Photoionization energy (eV)
1	$CH_4 + O_2$	0 nA, blank	11.50
2	$CH_4 + O_2$	50 nA, 30 min	11.50
3	$CH_4 + O_2$	50 nA, 30 min	10.49
4	$CD_4 + O_2$	50 nA, 30 min	11.50
5	$CH_4 + {}^{18}O_2$	50 nA, 30 min	11.50
6	$CH_4 + O_2$	50 nA, 30 min	9.20 to 11.00
7	$CH_4 + O_2$	50 nA, 30 min	9.20 to 11.50
8	$CH_4 + O_2$	50 nA, 30 min	9.80 to 11.00
9	$CH_4 + O_2$	50 nA, 30 min	9.80 to 11.50

Supplementary Table 5. Data applied to calculate the average dose per molecule.

Initial kinetic energy of the electrons, E_{init}	5 keV
Irradiation current, I	50 ± 2 nA
Irradiation time, t	1800 s
Total number of electrons	$(5.6 \pm 0.6) \times 10^{14}$
Average kinetic energy of backscattered electrons, E_{bs}*	3.3 ± 0.3 keV
Fraction of backscattered electrons, f_{bs}*	0.35 ± 0.03
Average kinetic energy of transmitted electrons, E_{trans}*	0.9 ± 0.09 keV
Fraction of transmitted electrons, f_{trans}*	0.003 ± 0.0003
Average penetration depth, l*	292 ± 29 nm
Density of the ice, ρ	0.975 g cm^{-3}
Dose per molecule	2.23 eV/per CH_4 molecule
	4.45 eV/per O_2 molecule

Note:

* Parameters obtained using the CASINO software v2.42[4].

Supplementary Table 6. Energies, geometries, coordinates and frequencies of all the species calculated in the reaction network exploration.

No.	Name	Energies (a.u.)	Atom	X (Å)	Y (Å)	Z (Å)	ν (cm^{-1})	T1 diag.
**0**	cCH₂OO<br>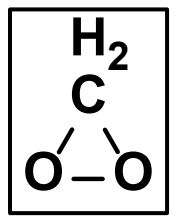	ZPE(ωB97X-D/def2-TZVP)=0.0330237<br>E(ωB97X-D/def2-TZVP)=−189.6368892<br>E(UCCSD(T)/aug-cc-pVDZ)=−189.2080745<br>E(UCCSD(T)/aug-cc-pVTZ)=−189.3725889	C<br>H<br>H<br>O<br>O	−0.141545<br>−0.243503<br>−0.104551<br>0.659268<br>−0.802440	0.721302<br>1.293811<br>1.295500<br>−0.396842<br>−0.483830	−0.007301<br>−0.928775<br>0.918014<br>−0.066449<br>0.043498	873.4,937.3,1040.7<br>1188.8,1275.4,1363.6<br>1562.6,3079.3,3174.6	0.0153
**1**	CH<br>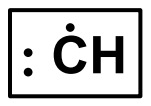	ZPE(ωB97X-D/def2-TZVP)=0.0065093<br>E(ωB97X-D/def2-TZVP)=−38.4768290<br>E(UCCSD(T)/aug-cc-pVDZ)=−38.3871579<br>E(UCCSD(T)/aug-cc-pVTZ)=−38.4128164	C<br>H	0.000000<br>0.000000	0.272895<br>1.379849	−0.692609<br>−0.506115	2857.2	0.0123
**2**	CH₂(OH)O<br>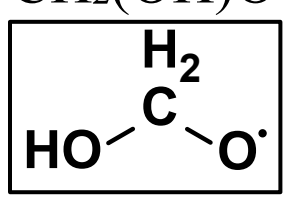	ZPE(ωB97X-D/def2-TZVP)=0.0421383<br>E(ωB97X-D/def2-TZVP)=−190.3002699<br>E(UCCSD(T)/aug-cc-pVDZ)=−189.8652768<br>E(UCCSD(T)/aug-cc-pVTZ)=−190.0286178	C<br>H<br>H<br>O<br>O<br>H	0.014418<br>−0.123952<br>−0.016101<br>1.275477<br>−1.039072<br>1.228962	0.496443<br>0.996284<br>1.316263<br>−0.090487<br>−0.294053<br>−0.961475	0.092136<br>1.082074<br>−0.647330<br>−0.000175<br>−0.037213<br>0.402181	206.1,557.0,740.7<br>1019.2,1134.9,1176.9<br>1290.5,1335.4,1405.7<br>2805.7,2951.8,3872.7	0.0235
**3**	CH₂OH<br>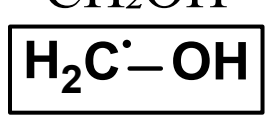	ZPE(ωB97X-D/def2-TZVP)=0.0375784<br>E(ωB97X-D/def2-TZVP)=−115.0713245<br>E(UCCSD(T)/aug-cc-pVDZ)=−114.7963050<br>E(UCCSD(T)/aug-cc-pVTZ)=−114.8987353	C<br>H<br>H<br>O<br>H	−0.657066<br>−0.284611<br>−0.951451<br>−1.302789<br>−1.059032	0.646799<br>0.914365<br>1.402409<br>−0.536234<br>−1.136186	0.026704<br>1.008093<br>−0.685505<br>−0.131447<br>0.574722	421.5,563.1,1069.4<br>1239.2,1368.8,1496.8<br>3145.5,3288.2,3902.5	0.0172
**4**	CH₂OHOH_a<br>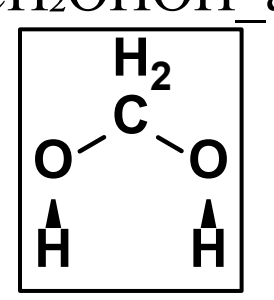	ZPE(ωB97X-D/def2-TZVP)=0.0574787<br>E(ωB97X-D/def2-TZVP)=−190.9704532<br>E(UCCSD(T)/aug-cc-pVDZ)=−190.5338556<br>E(UCCSD(T)/aug-cc-pVTZ)=−190.7033347	C<br>H<br>H<br>O<br>O<br>H<br>H	0.415346<br>0.992036<br>0.902418<br>0.399876<br>−0.855724<br>0.185891<br>−1.249122	−0.215246<br>−0.476078<br>−0.631849<br>1.170180<br>−0.796025<br>1.605359<br>−0.641110	0.118231<br>−0.779743<br>0.997539<br>0.304893<br>0.069575<br>−0.521640<br>−0.790445	191.1,396.9,552.3<br>1014.1,1091.7,1098.7<br>1179.1,1382.9,1419.1<br>1454.3,1524.4,2999.2<br>3144.3,3890.3,3891.9	0.0132

**5**	CH₂OHOH_b<br>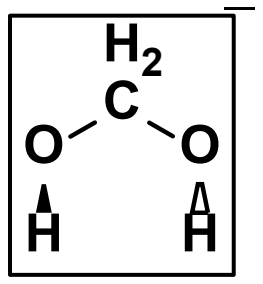	ZPE(ωB97X-D/def2-TZVP)=0.0579328<br>E(ωB97X-D/def2-TZVP)=−190.9749898<br>E(UCCSD(T)/aug-cc-pVDZ)=−190.5381671<br>E(UCCSD(T)/aug-cc-pVTZ)=−190.7075647	C<br>H<br>H<br>O<br>O<br>H<br>H	0.013448<br>−0.053748<br>−0.022932<br>1.231939<br>−1.077997<br>−1.124310<br>1.364420	0.588107<br>0.919560<br>1.455404<br>−0.089958<br>−0.204198<br>−0.948386<br>−0.343034	0.114249<br>1.154138<br>−0.550746<br>0.020781<br>−0.252123<br>0.352416<br>−0.895641	378.1,387.0,574.0<br>1026.3,1065.8,1104.6<br>1222.9,1381.0,1401.4<br>1460.0,1537.7,3047.0<br>3097.0,3873.1,3873.5	0.0132
**6**	CH₂OO<br>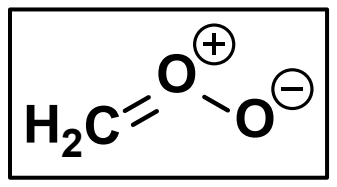	ZPE(ωB97X-D/def2-TZVP)=0.0316572<br>E(ωB97X-D/def2-TZVP)=−189.5943428<br>E(UCCSD(T)/aug-cc-pVDZ)=−189.1689756<br>E(UCCSD(T)/aug-cc-pVTZ)=−189.3307314	C<br>H<br>H<br>O<br>O	−0.938878<br>−1.620693<br>−1.081596<br>0.084721<br>0.973652	−0.717422<br>−1.146415<br>−0.715151<br>−0.190600<br>0.359490	0.034464<br>0.756287<br>−1.040815<br>0.511474<br>−0.308079	547.2,692.7,969.3<br>977.0,1260.3,1423.6<br>1589.5,3140.8,3295.5	0.0440
**7**	CH₂OOH<br>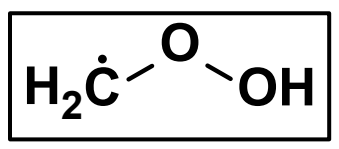	ZPE(ωB97X-D/def2-TZVP)=0.0402217<br>E(ωB97X-D/def2-TZVP)=−190.2064805<br>E(UCCSD(T)/aug-cc-pVDZ)=−189.7730729<br>E(UCCSD(T)/aug-cc-pVTZ)=−189.9382806	C<br>H<br>H<br>O<br>O<br>H	1.223979<br>1.562284<br>1.698189<br>−0.338164<br>−0.077587<br>−1.213581	−0.003543<br>0.257115<br>−0.737527<br>0.273938<br>0.261014<br>−0.129323	−0.009295<br>0.981525<br>−0.646755<br>−1.609449<br>−0.194185<br>−1.619419	69.4,197.0,450.6<br>515.2,877.7,1171.1<br>1186.2,1419.1,1464.8<br>3157.4,3310.7,3836.2	0.0215
**8**	CH₂[OH]O<br>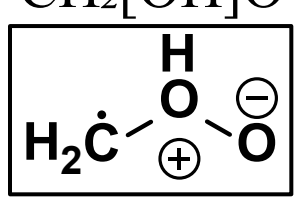	ZPE(ωB97X-D/def2-TZVP)=0.0409386<br>E(ωB97X-D/def2-TZVP)=−190.1265800<br>E(UCCSD(T)/aug-cc-pVDZ)=−189.7006825<br>E(UCCSD(T)/aug-cc-pVTZ)=−189.8631849	C<br>H<br>H<br>O<br>O<br>H	1.106672<br>1.410260<br>1.301274<br>−0.060750<br>−1.133665<br>−0.262536	−0.147619<br>−0.604262<br>−0.583285<br>0.561926<br>−0.553787<br>1.052129	0.036279<br>−0.890671<br>1.005945<br>−0.091915<br>−0.015690<br>0.713465	301.0,351.7,375.2<br>710.2,878.1,1078.9<br>1129.2,1330.2,1478.1<br>3175.4,3333.7,3828.2	0.0389
**9**	CH₂(¹A₁)<br>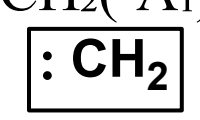	ZPE(ωB97X-D/def2-TZVP)=0.0167131<br>E(ωB97X-D/def2-TZVP)=−39.1311549<br>E(UCCSD(T)/aug-cc-pVDZ)=−39.0312486<br>E(UCCSD(T)/aug-cc-pVTZ)=−39.0645895	C<br>H<br>H	−4.467175<br>−4.165278<br>−5.574777	1.476831<br>0.408781<br>1.405473	−0.195508<br>−0.195508<br>−0.195508	1401.9,2930.3,3004.0	0.0099

**10**	$CH_2(^3B_1)$<br>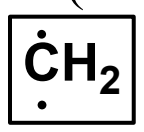	ZPE(ωB97X-D/def2-TZVP)=0.0172662<br>E(ωB97X-D/def2-TZVP)=−39.1508184<br>E(UCCSD(T)/aug-cc-pVDZ)=−39.0480635<br>E(UCCSD(T)/aug-cc-pVTZ)=−39.0800920	C<br>H<br>H	−4.574702<br>−4.003476<br>−5.629052	1.324770<br>0.408415<br>1.557901	−0.195508<br>−0.195508<br>−0.195508	1071.8,3138.2,3369.0	0.0115
**11**	CH_3<br>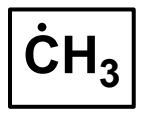	ZPE(ωB97X-D/def2-TZVP)=0.0298099<br>E(ωB97X-D/def2-TZVP)=−39.8386404<br>E(UCCSD(T)/aug-cc-pVDZ)=−39.7243287<br>E(UCCSD(T)/aug-cc-pVTZ)=−39.7636609	C<br>H<br>H<br>H	1.348745<br>1.909156<br>1.090844<br>1.046056	−0.361247<br>0.561281<br>−0.828027<br>−0.816807	0.026979<br>0.034777<br>−0.911519<br>0.957561	522.8,1410.2,1410.4<br>3125.9,3307.6,3308.1	0.0087
**12**	CH_3O<br>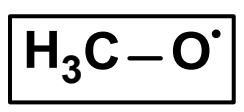	ZPE(ωB97X-D/def2-TZVP)=0.0366227<br>E(ωB97X-D/def2-TZVP)=−115.0604406<br>E(UCCSD(T)/aug-cc-pVDZ)=−114.7841384<br>E(UCCSD(T)/aug-cc-pVTZ)=−114.8848478	C<br>H<br>H<br>H<br>O	−0.933643<br>−1.927109<br>−0.890110<br>−0.890110<br>−0.036133	−0.467428<br>0.019553<br>−1.084598<br>−1.084598<br>0.550015	0.000000<br>0.000000<br>0.907898<br>−0.907898<br>0.000000	726.2,970.8,1141.6<br>1371.5,1376.6,1520.5<br>2927.9,2999.3,3041.1	0.0193
**13**	CH_3OH<br>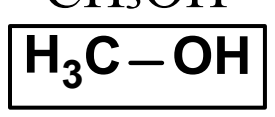	ZPE(ωB97X-D/def2-TZVP)=0.0516799<br>E(ωB97X-D/def2-TZVP)=−115.7349461<br>E(UCCSD(T)/aug-cc-pVDZ)=−115.4551188<br>E(UCCSD(T)/aug-cc-pVTZ)=−115.5622313	C<br>H<br>H<br>H<br>O<br>H	−0.960392<br>−1.939306<br>−0.889281<br>−0.889281<br>0.002142<br>0.874043	−0.462386<br>0.015299<br>−1.096738<br>−1.096738<br>0.568303<br>0.173664	0.000000<br>0.000000<br>0.890941<br>−0.890941<br>0.000000<br>0.000000	310.3,1074.1,1110.1<br>1184.6,1380.5,1490.8<br>1503.5,1515.8,3008.7<br>3062.0,3136.7,3907.8	0.0109
**14**	CH_3OHO<br>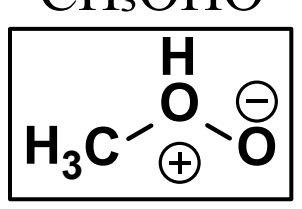	ZPE(ωB97X-D/def2-TZVP)=0.0560233<br>E(ωB97X-D/def2-TZVP)=−190.8010216<br>E(UCCSD(T)/aug-cc-pVDZ)=−190.3674969<br>E(UCCSD(T)/aug-cc-pVTZ)=−190.5363664	C<br>H<br>H<br>H<br>O<br>O<br>H	−1.187802<br>−1.401775<br>−1.436459<br>−1.683199<br>0.929945<br>0.230458<br>0.534099	−0.267821<br>0.343471<br>0.270181<br>−1.238958<br>0.799270<br>−0.497472<br>−0.940642	−0.012383<br>−0.886705<br>0.895752<br>−0.047956<br>0.000489<br>0.061343<br>−0.739707	232.0,380.3,734.4<br>892.2,1007.6,1118.2<br>1192.1,1346.2,1460.2<br>1467.2,1505.1,3071.0<br>3170.2,3211.5,3803.1	0.0188

**15**	CH_3OO<br>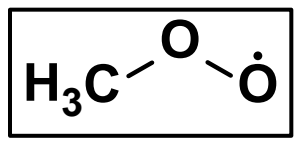	ZPE(ωB97X-D/def2-TZVP)=0.0434364<br>E(ωB97X-D/def2-TZVP)=−190.2336435<br>E(UCCSD(T)/aug-cc-pVDZ)=−189.7963035<br>E(UCCSD(T)/aug-cc-pVTZ)=−189.9605672	C<br>H<br>H<br>H<br>O<br>O	1.134593<br>1.950426<br>1.192552<br>1.131122<br>−0.069076<br>−1.122266	−0.231186<br>0.488803<br>−0.866682<br>−0.834709<br>0.546397<br>−0.214036	0.013532<br>0.028670<br>−0.869409<br>0.920476<br>−0.041669<br>−0.064231	141.7,509.6,960.8<br>1141.7,1196.7,1282.0<br>1457.2,1479.6,1490.3<br>3066.3,3161.7,3178.8	0.0289
**16**	CH_3OOH<br>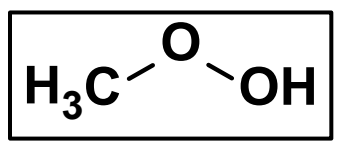	ZPE(ωB97X-D/def2-TZVP)=0.0553619<br>E(ωB97X-D/def2-TZVP)=−190.8733545<br>E(UCCSD(T)/aug-cc-pVDZ)=−190.4353604<br>E(UCCSD(T)/aug-cc-pVTZ)=−190.6050957	C<br>H<br>H<br>H<br>O<br>O<br>H	1.109145<br>1.959097<br>1.177797<br>1.103174<br>−0.022618<br>−1.160234<br>−1.588883	−0.210433<br>0.473040<br>−0.887515<br>−0.788356<br>0.619227<br>−0.234790<br>−0.041842	0.029011<br>0.025972<br>−0.826050<br>0.958559<br>−0.070616<br>−0.134172<br>0.706633	201.0,253.4,465.9<br>937.0,1094.1,1182.5<br>1225.6,1403.4,1460.2<br>1471.7,1515.9,3025.1<br>3098.4,3140.7,3826.2	0.0135
**17**	CH_4<br>**CH_4**	ZPE(ωB97X-D/def2-TZVP)=0.0448640<br>E(ωB97X-D/def2-TZVP)=−40.5192789<br>E(UCCSD(T)/aug-cc-pVDZ)=−40.3953368<br>E(UCCSD(T)/aug-cc-pVTZ)=−40.4409274	C<br>H<br>H<br>H<br>H	0.831431<br>1.194374<br>1.194393<br>1.194392<br>−0.257433	2.176925<br>1.150329<br>2.690216<br>2.690217<br>2.176938	0.000000<br>−0.000001<br>0.889054<br>−0.889054<br>0.000001	1342.5,1342.5,1342.5<br>1560.9,1560.9,3045.9<br>3165.9,3165.9,3165.9	0.0068
**18**	CHOHOH_E<br>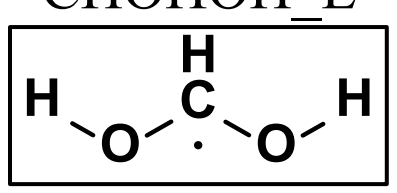	ZPE(ωB97X-D/def2-TZVP)=0.0435992<br>E(ωB97X-D/def2-TZVP)=−190.3077857<br>E(UCCSD(T)/aug-cc-pVDZ)=−189.8740407<br>E(UCCSD(T)/aug-cc-pVTZ)=−190.0391615	C<br>H<br>O<br>O<br>H<br>H	0.000497<br>−0.001792<br>1.104918<br>−1.104022<br>1.880506<br>−1.879756	0.116793<br>0.561897<br>−0.598067<br>−0.594999<br>−0.074401<br>−0.075712	0.342016<br>1.346187<br>0.005434<br>−0.000460<br>0.210639<br>0.215068	157.6,345.8,544.4<br>971.8,1101.6,1135.8<br>1252.3,1360.7,1445.3<br>2989.9,3915.5,3917.1	0.0171
**19**	CHOHOH_Z<br>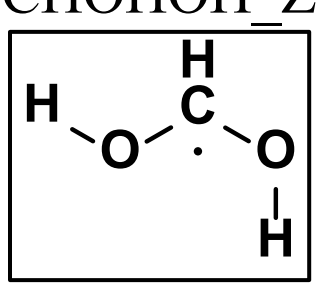	ZPE(ωB97X-D/def2-TZVP)=0.0438930<br>E(ωB97X-D/def2-TZVP)=−190.3116549<br>E(UCCSD(T)/aug-cc-pVDZ)=−189.8779079<br>E(UCCSD(T)/aug-cc-pVTZ)=−190.0430100	C<br>H<br>O<br>O<br>H<br>H	0.042100<br>−0.114457<br>1.191935<br>−1.013074<br>1.218405<br>−1.744838	0.327480<br>1.381577<br>−0.139863<br>−0.523882<br>−1.092434<br>−0.257444	0.262295<br>0.031677<br>−0.270764<br>0.044216<br>−0.142661<br>0.603262	282.4,356.9,546.0<br>947.6,1088.2,1161.1<br>1216.5,1375.6,1427.6<br>3102.2,3860.4,3902.3	0.0173

**20**	CHOOH_E<br>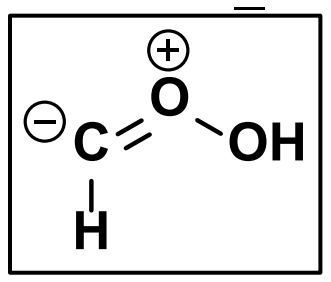	ZPE(ωB97X-D/def2-TZVP)=0.0283947<br>E(ωB97X-D/def2-TZVP)=−189.5895682<br>E(UCCSD(T)/aug-cc-pVDZ)=−189.1850605<br>E(UCCSD(T)/aug-cc-pVTZ)=−189.3419103	C<br>H<br>O<br>O<br>H	0.772589<br>1.732112<br>−0.124088<br>0.250619<br>−0.574637	−0.649196<br>−0.605914<br>0.066814<br>1.206990<br>1.702016	0.277074<br>0.812573<br>0.513151<br>1.815880<br>1.874865	180.8,368.1,375.7<br>851.5,951.2,1035.3<br>1828.6,3027.7,3845.0	0.0338
**21**	CHOOH_Z<br>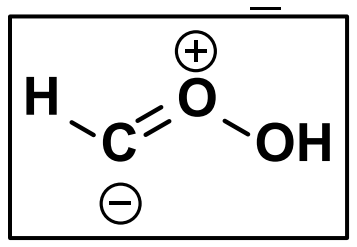	ZPE(ωB97X-D/def2-TZVP)=0.0295618<br>E(ωB97X-D/def2-TZVP)=−189.5753506<br>E(UCCSD(T)/aug-cc-pVDZ)=−189.1549391<br>E(UCCSD(T)/aug-cc-pVTZ)=−189.3144780	C<br>H<br>O<br>O<br>H	0.747352<br>1.570733<br>−0.132410<br>−1.385227<br>−1.925603	−0.660382<br>−0.385728<br>0.145334<br>−0.034574<br>0.659919	0.252981<br>0.943703<br>0.585842<br>−0.233404<br>0.166770	215.4,492.6,589.9<br>869.3,1239.5,1351.0<br>1466.9,2946.1,3805.5	0.0293
**22**	CH[OH]O_E<br>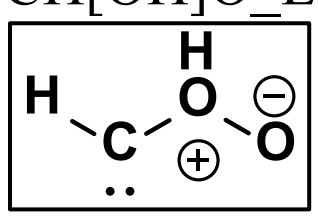	ZPE(ωB97X-D/def2-TZVP)=0.0300694<br>E(ωB97X-D/def2-TZVP)=−189.4647251<br>E(UCCSD(T)/aug-cc-pVDZ)=−189.0426587<br>E(UCCSD(T)/aug-cc-pVTZ)=−189.2033375	C<br>H<br>O<br>O<br>H	−0.736910<br>−1.765175<br>−0.478659<br>0.706219<br>−1.097804	−1.223948<br>−1.110062<br>−0.018154<br>0.435638<br>0.732647	−0.614414<br>−1.017184<br>−0.186622<br>0.433663<br>−0.248058	512.0,526.7,769.5<br>906.5,1228.9,1260.5<br>1459.2,2928.7,3607.1	0.0346
**23**	CH[OH]O_Z<br>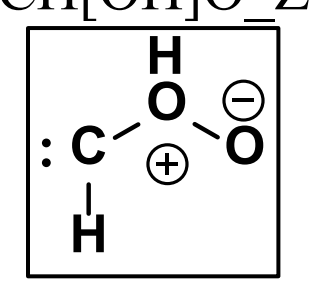	ZPE(ωB97X-D/def2-TZVP)=0.0306711<br>E(ωB97X-D/def2-TZVP)=−189.4790225<br>E(UCCSD(T)/aug-cc-pVDZ)=−189.0568915<br>E(UCCSD(T)/aug-cc-pVTZ)=−189.2174815	C<br>H<br>O<br>O<br>H	1.208495<br>1.078727<br>−1.192358<br>0.016705<br>−0.121363	0.437429<br>0.037625<br>−0.195715<br>0.314149<br>0.607521	0.176953<br>1.197736<br>0.238622<br>−0.323818<br>−1.238690	476.1,528.7,760.5<br>997.8,1222.2,1291.7<br>1446.4,3014.2,3725.5	0.0358
**24**	CO<br>**CO**	ZPE(ωB97X-D/def2-TZVP)=0.0051192<br>E(ωB97X-D/def2-TZVP)=−113.3216803<br>E(UCCSD(T)/aug-cc-pVDZ)=−113.0727648<br>E(UCCSD(T)/aug-cc-pVTZ)=−113.1618211	C<br>O	0.000000<br>0.000000	0.437546<br>0.344531	0.249460<br>1.368540	2247.1	0.0187

**25**	CO_2<br>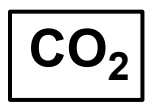	ZPE(ωB97X-D/def2-TZVP)=0.0119241<br>E(ωB97X-D/def2-TZVP)=−188.6055738<br>E(UCCSD(T)/aug-cc-pVDZ)=−188.1842014<br>E(UCCSD(T)/aug-cc-pVTZ)=−188.3400963	C<br>O<br>O	−0.000024<br>−1.155804<br>1.155755	0.150019<br>0.149964<br>0.150074	−0.000011<br>−0.000011<br>−0.000011	689.7,689.7,1400.0<br>2454.7	0.0182
**26**	COH<br>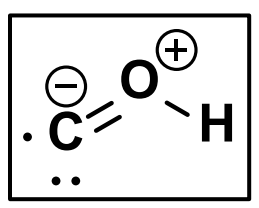	ZPE(ωB97X-D/def2-TZVP)=0.0137697<br>E(ωB97X-D/def2-TZVP)=−113.7936082<br>E(UCCSD(T)/aug-cc-pVDZ)=−113.5358717<br>E(UCCSD(T)/aug-cc-pVTZ)=−113.6274238	C<br>H<br>O	0.737701<br>−1.149431<br>−0.329397	0.678985<br>0.532856<br>−0.002763	0.000000<br>0.000000<br>0.000000	1152.0,1433.9,3458.3	0.0212
**27**	COH_2<br>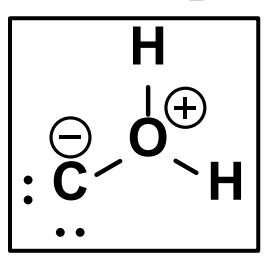	ZPE(ωB97X-D/def2-TZVP)=0.0238181<br>E(ωB97X-D/def2-TZVP)=−114.2516322<br>E(UCCSD(T)/aug-cc-pVDZ)=−113.9930564<br>E(UCCSD(T)/aug-cc-pVTZ)=−114.0894161	C<br>H<br>H<br>O	0.087820<br>0.097017<br>−0.046787<br>0.042294	0.002309<br>−0.798451<br>0.820450<br>0.008600	−0.909198<br>1.087248<br>1.075392<br>0.543529	481.9,553.7,904.9<br>1620.4,3407.2,3486.9	0.0161
**28**	COHOH_E<br>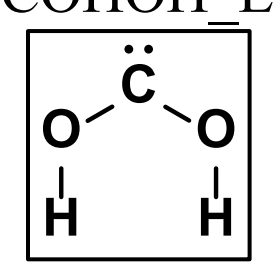	ZPE(ωB97X-D/def2-TZVP)=0.0321289<br>E(ωB97X-D/def2-TZVP)=−189.7016988<br>E(UCCSD(T)/aug-cc-pVDZ)=−189.2770510<br>E(UCCSD(T)/aug-cc-pVTZ)=−189.4386701	C<br>O<br>O<br>H<br>H	−0.000002<br>1.095747<br>−1.095739<br>0.954900<br>−0.954877	0.400133<br>−0.185261<br>−0.185291<br>−0.976390<br>−0.976417	−0.377908<br>0.037267<br>0.037255<br>0.598116<br>0.598104	584.0,674.5,755.3<br>1163.3,1171.6,1352.9<br>1375.1,3493.2,3533.1	0.0200
**29**	COHOH_Z<br>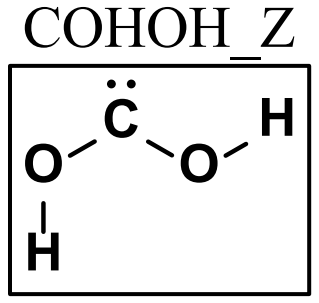	ZPE(ωB97X-D/def2-TZVP)=0.0335235<br>E(ωB97X-D/def2-TZVP)=−189.7148680<br>E(UCCSD(T)/aug-cc-pVDZ)=−189.2894924<br>E(UCCSD(T)/aug-cc-pVTZ)=−189.4509756	C<br>O<br>O<br>H<br>H	0.014504<br>1.015705<br>−1.100225<br>−0.936242<br>1.837000	0.316938<br>−0.355362<br>−0.195419<br>−0.931625<br>0.021415	−0.432421<br>0.127324<br>0.008668<br>0.628315<br>−0.198118	641.9,667.4,788.2<br>1148.7,1182.2,1359.4<br>1436.2,3607.9,3883.2	0.0185

**30**	COOH<br>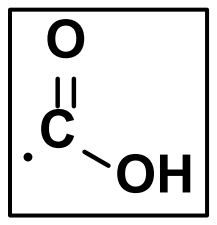	ZPE(ωB97X-D/def2-TZVP)=0.0210007<br>E(ωB97X-D/def2-TZVP)=−189.1144202<br>E(UCCSD(T)/aug-cc-pVDZ)=−188.6909429<br>E(UCCSD(T)/aug-cc-pVTZ)=−188.8485579	C<br>O<br>O<br>H	0.138549<br>−1.101867<br>1.162040<br>−1.095440	0.360485<br>−0.087240<br>−0.223841<br>−1.059238	0.000013<br>0.000032<br>0.000009<br>0.000046	597.4,612.7,1110.5<br>1313.7,1904.8,3679.1	0.0234
**31**	C(^{1}D)<br>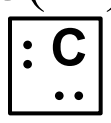	ZPE(ωB97X-D/def2-TZVP)=0.0000000<br>E(ωB97X-D/def2-TZVP)=−37.7759872<br>E(UCCSD(T)/aug-cc-pVDZ)=−37.7075603<br>E(UCCSD(T)/aug-cc-pVTZ)=−37.7294570	C	0.000000	0.000000	0.000000		0.0056
**32**	H<br>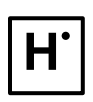	ZPE(ωB97X-D/def2-TZVP)=0.0000000<br>E(ωB97X-D/def2-TZVP)=−0.5026664<br>E(UCCSD(T)/aug-cc-pVDZ)=−0.4993343<br>E(UCCSD(T)/aug-cc-pVTZ)=−0.4998212	H	0.000000	0.000000	0.000000		0.0000
**33**	H_2<br>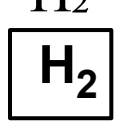	ZPE(ωB97X-D/def2-TZVP)=0.0101110<br>E(ωB97X-D/def2-TZVP)=−1.1761668<br>E(UCCSD(T)/aug-cc-pVDZ)=−1.1646945<br>E(UCCSD(T)/aug-cc-pVTZ)=−1.1726347	H<br>H	−0.072078<br>0.672078	0.000000<br>0.000000	0.000000<br>0.000000	4438.2	0.0048
**34**	H_2O<br>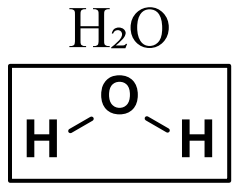	ZPE(ωB97X-D/def2-TZVP)=0.0216233<br>E(ωB97X-D/def2-TZVP)=−76.4376926<br>E(UCCSD(T)/aug-cc-pVDZ)=−76.2737155<br>E(UCCSD(T)/aug-cc-pVTZ)=−76.3422632	H<br>O<br>H	0.493943<br>1.047167<br>1.947673	−0.822688<br>−0.041338<br>−0.366359	−0.000619<br>0.001621<br>−0.002795	1623.8,3878.9,3988.9	0.0123

**35**	H_2O_2<br>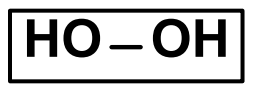	ZPE(ωB97X-D/def2-TZVP)=0.0271257<br>E(ωB97X-D/def2-TZVP)=−151.5669478<br>E(UCCSD(T)/aug-cc-pVDZ)=−151.2418787<br>E(UCCSD(T)/aug-cc-pVTZ)=−151.3736397	O<br>H<br>O<br>H	0.000000<br>−0.792909<br>0.000000<br>0.792909	−0.712403<br>−0.899499<br>0.712403<br>0.899499	−0.056971<br>0.455766<br>−0.056971<br>0.455766	389.3,1019.6,1353.7<br>1475.8,3833.9,3834.5	0.0141
**36**	H_2OO<br>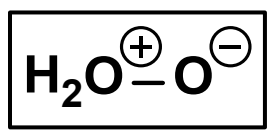	ZPE(ωB97X-D/def2-TZVP)=0.0267854<br>E(ωB97X-D/def2-TZVP)=−151.4904591<br>E(UCCSD(T)/aug-cc-pVDZ)=−151.1703003<br>E(UCCSD(T)/aug-cc-pVTZ)=−151.3009159	O<br>H<br>O<br>H	0.236813<br>−0.362376<br>−0.281832<br>1.092905	−0.645656<br>−1.183930<br>0.755390<br>−0.631079	−0.132913<br>0.399019<br>0.085906<br>0.312408	709.7,903.3,911.4<br>1602.8,3764.9,3865.3	0.0208
**37**	HCHO<br>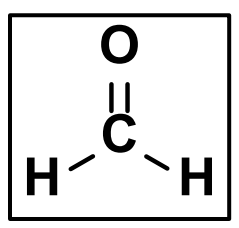	ZPE(ωB97X-D/def2-TZVP)=0.0267899<br>E(ωB97X-D/def2-TZVP)=−114.5116957<br>E(UCCSD(T)/aug-cc-pVDZ)=−114.2439394<br>E(UCCSD(T)/aug-cc-pVTZ)=−114.3425224	C<br>H<br>H<br>O	−1.103388<br>−1.463832<br>−1.464012<br>−0.368350	−0.238307<br>−0.700605<br>−0.700250<br>0.703843	−0.000061<br>0.939084<br>−0.939310<br>0.000047	1212.7,1275.2,1540.1<br>1857.7,2906.1,2967.5	0.0162
**38**	HCO<br>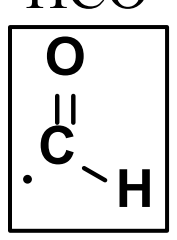	ZPE(ωB97X-D/def2-TZVP)=0.0131906<br>E(ωB97X-D/def2-TZVP)=−113.8601771<br>E(UCCSD(T)/aug-cc-pVDZ)=−113.5988711<br>E(UCCSD(T)/aug-cc-pVTZ)=−113.6921739	C<br>H<br>O	0.000000<br>0.000000<br>0.000000	0.007192<br>0.942269<br>−0.010264	−0.503575<br>−1.125305<br>0.665074	1117.0,1976.3,2696.7	0.0223
**39**	$HCOH_2$<br>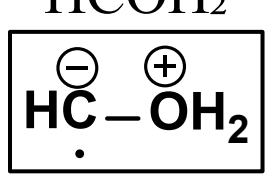	ZPE(ωB97X-D/def2-TZVP)=0.0346867<br>E(ωB97X-D/def2-TZVP)=−114.9395804<br>E(UCCSD(T)/aug-cc-pVDZ)=−114.6774366<br>E(UCCSD(T)/aug-cc-pVTZ)=−114.7741630	C<br>H<br>H<br>O<br>H	0.995722<br>0.801555<br>−0.527820<br>−0.654522<br>−1.236067	0.276661<br>1.362631<br>−1.127203<br>−0.174256<br>0.016699	−0.432199<br>−0.272830<br>0.139752<br>0.127984<br>−0.615606	164.8,365.3,612.3<br>652.7,1166.3,1627.1<br>2892.1,3821.3,3923.7	0.0191

**40**	HCOHOH<br>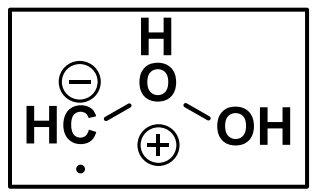	ZPE(ωB97X-D/def2-TZVP)=0.0397868<br>E(ωB97X-D/def2-TZVP)=−190.0781536<br>E(UCCSD(T)/aug-cc-pVDZ)=−189.6546760<br>E(UCCSD(T)/aug-cc-pVTZ)=−189.8163861	C<br>H<br>H<br>O<br>O<br>H	−0.857570<br>−0.741218<br>−0.564325<br>0.428250<br>0.141050<br>1.167831	0.957526<br>−1.032135<br>2.005864<br>0.266792<br>−1.129718<br>0.355543	−0.441937<br>−0.246680<br>−0.228158<br>0.247219<br>0.185206<br>−0.374032	210.6,388.5,436.8<br>540.9,764.8,945.0<br>1219.6,1304.5,1513.4<br>2947.4,3455.1,3737.8	0.0180
**41**	HCOH_E<br>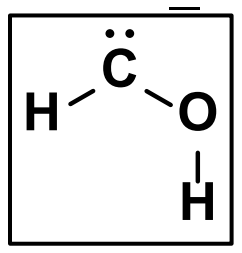	ZPE(ωB97X-D/def2-TZVP)=0.0263161<br>E(ωB97X-D/def2-TZVP)=−114.4194372<br>E(UCCSD(T)/aug-cc-pVDZ)=−114.1560252<br>E(UCCSD(T)/aug-cc-pVTZ)=−114.2533036	C<br>H<br>O<br>H	−0.636074<br>−0.659028<br>−1.455854<br>−1.910206	0.719742<br>1.280892<br>−0.282371<br>−0.357897	0.154130<br>−0.816014<br>0.039120<br>−0.815092	1025.2,1246.0,1372.5<br>1480.4,2777.8,3649.4	0.0196
**42**	HCOH_Z<br>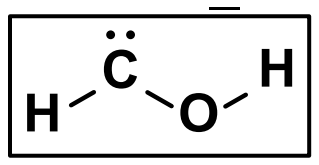	ZPE(ωB97X-D/def2-TZVP)=0.0270391<br>E(ωB97X-D/def2-TZVP)=−114.4277848<br>E(UCCSD(T)/aug-cc-pVDZ)=−114.1636855<br>E(UCCSD(T)/aug-cc-pVTZ)=−114.2609166	C<br>H<br>O<br>H	−0.756803<br>−1.606223<br>−1.360782<br>−0.696065	0.859559<br>1.570902<br>−0.269871<br>−0.959976	0.135803<br>0.247725<br>−0.097346<br>−0.212248	1102.5,1226.2,1372.5<br>1512.8,2874.7,3780.1	0.0190
**43**	HCOO<br>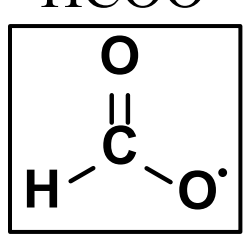	ZPE(ωB97X-D/def2-TZVP)=0.0174057<br>E(ωB97X-D/def2-TZVP)=−189.0953130<br>E(UCCSD(T)/aug-cc-pVDZ)=−188.6675190<br>E(UCCSD(T)/aug-cc-pVTZ)=−188.8240586	C<br>H<br>O<br>O	0.112153<br>0.061946<br>−1.108193<br>1.190669	0.286626<br>1.444552<br>0.033059<br>−0.225852	−0.000017<br>−0.000067<br>−0.000013<br>0.000012	506.4,878.0,886.2<br>1162.8,1778.9,2427.8	0.0513
**44**	HCOOH<br>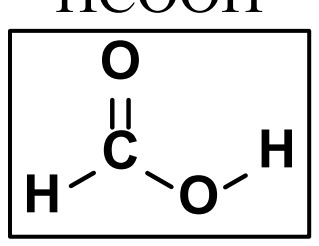	ZPE(ωB97X-D/def2-TZVP)=0.0342164<br>E(ωB97X-D/def2-TZVP)=−189.7842848<br>E(UCCSD(T)/aug-cc-pVDZ)=−189.3547525<br>E(UCCSD(T)/aug-cc-pVTZ)=−189.5174210	C<br>H<br>O<br>O<br>H	−0.069987<br>−0.019952<br>1.160549<br>−1.075207<br>1.079045	0.261676<br>1.212413<br>−0.138782<br>−0.326769<br>−0.975225	−0.297198<br>−0.843160<br>0.032180<br>−0.036267<br>0.509599	641.4,689.0,1070.0<br>1157.3,1323.2,1413.0<br>1856.6,3071.5,3797.3	0.0175

**45**	HCOOH_E<br>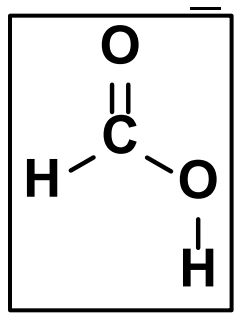	ZPE(ωB97X-D/def2-TZVP)=0.0338765<br>E(ωB97X-D/def2-TZVP)=−189.7772203<br>E(UCCSD(T)/aug-cc-pVDZ)=−189.3480061<br>E(UCCSD(T)/aug-cc-pVTZ)=−189.5106773	C<br>H<br>O<br>O<br>H	0.215520<br>0.283358<br>−1.044271<br>1.160579<br>−1.665327	0.415487<br>1.518643<br>−0.046249<br>−0.302556<br>0.687306	0.000000<br>0.000000<br>0.000000<br>0.000000<br>0.000000	532.8,674.1,1051.7<br>1143.3,1292.8,1433.7<br>1901.1,2971.9,3868.7	0.0174
**46**	HO_2<br>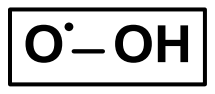	ZPE(ωB97X-D/def2-TZVP)=0.0145261<br>E(ωB97X-D/def2-TZVP)=−150.9243989<br>E(UCCSD(T)/aug-cc-pVDZ)=−150.5998479<br>E(UCCSD(T)/aug-cc-pVTZ)=−150.7260981	O<br>O<br>H	−0.065633<br>−1.106749<br>−1.573230	0.564564<br>−0.226941<br>−0.124049	−0.025624<br>−0.093740<br>0.752872	1243.5,1464.0,3668.8	0.0302
**47**	$O_2(^1\Delta g)$<br>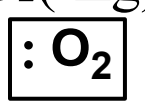	ZPE(ωB97X-D/def2-TZVP)=0.0038893<br>E(ωB97X-D/def2-TZVP)=−150.2784895<br>E(UCCSD(T)/aug-cc-pVDZ)=−149.9701883<br>E(UCCSD(T)/aug-cc-pVTZ)=−150.0923200	O<br>O	−3.582306<br>−4.776113	1.520875<br>1.520875	0.000000<br>0.000000	1707.2	0.0157
**48**	$O_2(^3\Sigma g^-)$<br>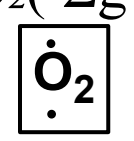	ZPE(ωB97X-D/def2-TZVP)=0.0039090<br>E(ωB97X-D/def2-TZVP)=−150.3400642<br>E(UCCSD(T)/aug-cc-pVDZ)=−150.0201322<br>E(UCCSD(T)/aug-cc-pVTZ)=−150.1405331	O<br>O	−3.581930<br>−4.776489	1.520875<br>1.520875	0.000000<br>0.000000	1715.9	0.0174
**49**	OH<br>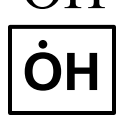	ZPE(ωB97X-D/def2-TZVP)=0.0085904<br>E(ωB97X-D/def2-TZVP)=−75.7410339<br>E(UCCSD(T)/aug-cc-pVDZ)=−75.5840131<br>E(UCCSD(T)/aug-cc-pVTZ)=−75.6455812	H<br>O	0.000000<br>0.000000	−0.767241<br>0.005406	−0.467959<br>0.120075	3770.8	0.0121

**50**	O(¹D)<br>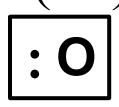	ZPE(ωB97X-D/def2-TZVP)=0.0000000<br>E(ωB97X-D/def2-TZVP)=−74.9688626<br>E(UCCSD(T)/aug-cc-pVDZ)=−74.8424152<br>E(UCCSD(T)/aug-cc-pVTZ)=−74.8979500	O	0.000000	0.000000	0.000000		0.0073
**51**	O(³P)<br>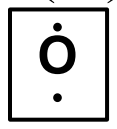	ZPE(ωB97X-D/def2-TZVP)=0.0000000<br>E(ωB97X-D/def2-TZVP)=−75.0708844<br>E(UCCSD(T)/aug-cc-pVDZ)=−74.9256536<br>E(UCCSD(T)/aug-cc-pVTZ)=−74.9789523	O	0.000000	0.000000	0.000000		0.0095
**52**	TS_cCH₂OO→CH₂<br>OO	ZPE(ωB97X-D/def2-TZVP)=0.0300783<br>E(ωB97X-D/def2-TZVP)=−189.5593383<br>E(UCCSD(T)/aug-cc-pVDZ)=−189.1387694<br>E(UCCSD(T)/aug-cc-pVTZ)=−189.2996200	C<br>O<br>O<br>H<br>H	−0.651942<br>−0.216161<br>−1.319127<br>0.066077<br>−1.694723	0.688702<br>−0.443969<br>−0.634912<br>1.506736<br>0.813636	−0.088041<br>0.253083<br>1.177466<br>−0.184068<br>−0.359566	−754.8,785.1,810.8<br>1058.8,1218.0,1435.1<br>1595.2,3073.3,3226.6	0.0532
**53**	TS_CH₂(OH)O→<br>HCOOH+H	ZPE(ωB97X-D/def2-TZVP)=0.0358874<br>E(ωB97X-D/def2-TZVP)=−190.2703109<br>E(UCCSD(T)/aug-cc-pVDZ)=−189.8386044<br>E(UCCSD(T)/aug-cc-pVTZ)=−190.0021719	C<br>H<br>H<br>O<br>O<br>H	−0.125970<br>−0.261958<br>−0.109505<br>1.131151<br>−1.123750<br>1.068067	0.345716<br>1.278346<br>1.301571<br>−0.125264<br>−0.329232<br>−1.018247	−0.145657<br>1.230026<br>−0.690176<br>−0.013833<br>0.012165<br>0.347433	−1138.2,489.6,526.0<br>635.6,655.8,1109.3<br>1123.5,1303.6,1375.1<br>1700.8,3020.9,3812.4	0.0261
**54**	TS_CH₂OH→CH₃<br>O	ZPE(ωB97X-D/def2-TZVP)=0.0325474<br>E(ωB97X-D/def2-TZVP)=−115.0066199<br>E(UCCSD(T)/aug-cc-pVDZ)=−114.7301695<br>E(UCCSD(T)/aug-cc-pVTZ)=−114.8327648	C<br>H<br>H<br>O<br>H	0.437592<br>0.970147<br>1.018449<br>−0.835906<br>−0.630167	0.266744<br>0.866761<br>−0.342963<br>−0.143010<br>0.763228	−0.074440<br>−0.812123<br>0.617422<br>−0.378158<br>0.381777	−2029.9,748.2,983.0<br>1149.3,1200.5,1487.6<br>2484.1,3061.4,3172.6	0.0202

**55**	TS_CH_2OH→HCHO+H	ZPE(ωB97X-D/def2-TZVP)=0.0279547<br>E(ωB97X-D/def2-TZVP)=−114.9976134<br>E(UCCSD(T)/aug-cc-pVDZ)=−114.7269086<br>E(UCCSD(T)/aug-cc-pVTZ)=−114.8267831	C<br>H<br>H<br>O<br>H	−0.630849<br>−1.186534<br>−1.186133<br>0.566543<br>1.626166	0.003381<br>0.946793<br>−0.932036<br>−0.004064<br>0.031296	−0.049032<br>−0.152341<br>−0.210634<br>0.199060<br>−0.932966	−1393.8,128.1,575.0<br>1105.8,1250.4,1505.6<br>1680.8,2970.1,3054.9	0.0381
**56**	TS_CH_2OH→$HCOH_2$	ZPE(ωB97X-D/def2-TZVP)=0.0306676<br>E(ωB97X-D/def2-TZVP)=−114.9244116<br>E(UCCSD(T)/aug-cc-pVDZ)=−114.6586213<br>E(UCCSD(T)/aug-cc-pVTZ)=−114.7572283	C<br>H<br>H<br>O<br>H	0.720206<br>0.966154<br>−0.036595<br>−0.884426<br>−1.386473	0.186098<br>1.146256<br>−0.732356<br>0.002394<br>−0.247862	−0.519975<br>−0.048990<br>0.078481<br>0.111323<br>−0.673742	−1387.2,365.0,529.8<br>826.1,1124.5,1386.3<br>2327.7,3069.8,3832.3	0.0218
**57**	TS_CH_2OH→HCOH_Z+H	ZPE(ωB97X-D/def2-TZVP)=0.0274307<br>E(ωB97X-D/def2-TZVP)=−114.9293130<br>E(UCCSD(T)/aug-cc-pVDZ)=−114.6634824<br>E(UCCSD(T)/aug-cc-pVTZ)=−114.7612712	C<br>H<br>H<br>O<br>H	−0.438335<br>−2.786082<br>−0.611042<br>0.653244<br>0.885293	−0.401209<br>1.251811<br>−1.167512<br>0.184761<br>0.876917	−0.252851<br>0.248697<br>0.535490<br>0.147667<br>−0.483351	−268.4,73.1,115.3<br>1096.5,1223.4,1369.9<br>1500.7,2880.1,3781.8	0.0254
**58**	TS_CH_2OHOH_a<br>→<br>H_2+COHOH_E	ZPE(ωB97X-D/def2-TZVP)=0.0473457<br>E(ωB97X-D/def2-TZVP)=−190.8256996<br>E(UCCSD(T)/aug-cc-pVDZ)=−190.3891824<br>E(UCCSD(T)/aug-cc-pVTZ)=−190.5575120	C<br>H<br>H<br>O<br>O<br>H<br>H	−0.000105<br>−0.000039<br>0.000061<br>1.140755<br>−1.140792<br>1.067705<br>−1.067415	0.504465<br>1.657944<br>1.664305<br>−0.150989<br>−0.151120<br>−0.850472<br>−0.850661	−0.287664<br>1.017365<br>−0.008914<br>−0.073041<br>−0.072748<br>0.590509<br>0.590683	−1404.9,377.2,525.9<br>609.6,716.3,748.2<br>1097.4,1174.6,1271.1<br>1369.5,1372.2,1622.2<br>2470.4,3699.0,3728.9	0.0180
**59**	TS_CH_2OHOH_a<br>→H_2+<br>HCOOH_HighBarrier	ZPE(ωB97X-D/def2-TZVP)=0.0435448<br>E(ωB97X-D/def2-TZVP)=−190.7671123<br>E(UCCSD(T)/aug-cc-pVDZ)=−190.3348567<br>E(UCCSD(T)/aug-cc-pVTZ)=−190.5020504	C<br>H<br>H<br>O<br>O<br>H<br>H	1.330593<br>2.168830<br>1.597149<br>1.931565<br>0.140418<br>−0.032404<br>0.670496	−0.120130<br>−0.382301<br>−0.609665<br>1.052260<br>−0.218776<br>1.269874<br>1.777791	0.014304<br>−0.783298<br>0.967034<br>−0.226700<br>−0.416961<br>0.158245<br>0.240767	−1718.3,417.3,545.2<br>712.1,793.7,942.3<br>1068.3,1224.4,1246.8<br>1256.4,1364.7,1498.9<br>2457.0,2624.2,2962.6	0.0255

**60**	TS_CH₂OHOH_a →H₂+ HCOOH_LowBarrier	ZPE(ωB97X-D/def2-TZVP)=0.0482580<br>E(ωB97X-D/def2-TZVP)=−190.8462201<br>E(UCCSD(T)/aug-cc-pVDZ)=−190.4049229<br>E(UCCSD(T)/aug-cc-pVTZ)=−190.5743600	C<br>H<br>H<br>O<br>O<br>H<br>H	−0.005402<br>−0.326135<br>0.008930<br>1.225755<br>−1.022503<br>1.137949<br>−1.018370	0.296014<br>0.751973<br>1.308737<br>−0.204105<br>−0.508511<br>−1.154043<br>0.158065	−0.067274<br>1.337108<br>−0.481247<br>−0.046829<br>−0.242573<br>0.106695<br>0.882624	−2337.6,521.9,573.9<br>671.8,815.2,1124.5<br>1156.4,1269.6,1345.3<br>1403.9,1502.6,1848.0<br>2069.4,3079.2,3801.0	0.0185
**61**	TS_CH₂OHOH_a → H₂O+HCOH_E	ZPE(ωB97X-D/def2-TZVP)=0.0499246<br>E(ωB97X-D/def2-TZVP)=−190.8480961<br>E(UCCSD(T)/aug-cc-pVDZ)=−190.4162178<br>E(UCCSD(T)/aug-cc-pVTZ)=−190.5826795	C<br>H<br>H<br>O<br>O<br>H<br>H	0.529429<br>0.723804<br>−0.629647<br>1.116587<br>−1.461794<br>1.498840<br>−1.776996	0.425930<br>0.728212<br>0.654906<br>−0.690336<br>0.039670<br>−1.162554<br>0.652301	−0.034452<br>1.001431<br>−0.475397<br>−0.371375<br>0.159147<br>0.378163<br>0.830987	−1320.3,249.7,397.8<br>474.4,681.6,751.1<br>1051.1,1222.9,1332.2<br>1446.7,1537.3,2091.8<br>3053.2,3766.2,3858.4	0.0222
**62**	TS_CH₂OHOH_b → H₂+COHOH_Z	ZPE(ωB97X-D/def2-TZVP)=0.0476002<br>E(ωB97X-D/def2-TZVP)=−190.8330116<br>E(UCCSD(T)/aug-cc-pVDZ)=−190.3966590<br>E(UCCSD(T)/aug-cc-pVTZ)=−190.5648276	C<br>H<br>H<br>O<br>O<br>H<br>H	0.422828<br>−0.545625<br>0.496609<br>0.183989<br>−0.119453<br>−0.816180<br>0.695867	0.195625<br>−0.268642<br>−0.003560<br>−0.809617<br>1.328569<br>1.158702<br>−1.579358	0.243242<br>1.677073<br>1.386078<br>−0.609946<br>−0.159917<br>−0.807856<br>−0.349302	−1437.7,307.2,500.4<br>587.8,686.0,707.1<br>1098.3,1205.2,1263.6<br>1335.2,1452.1,1507.5<br>2613.7,3755.8,3874.2	0.0195
**63**	TS_CH₂OHOH_b → H₂O+HCHO	ZPE(ωB97X-D/def2-TZVP)=0.0508336<br>E(ωB97X-D/def2-TZVP)=−190.8954240<br>E(UCCSD(T)/aug-cc-pVDZ)=−190.4602587<br>E(UCCSD(T)/aug-cc-pVTZ)=−190.6294826	C<br>H<br>H<br>O<br>O<br>H<br>H	−0.011510<br>−0.862342<br>0.903922<br>−0.230074<br>0.398264<br>−0.314342<br>0.116082	−0.277646<br>0.249170<br>−0.215960<br>−1.329037<br>0.681993<br>1.295115<br>−0.403637	0.704333<br>1.165664<br>1.306646<br>−0.022839<br>−0.608627<br>−0.822947<br>−0.913431	−1716.3,428.2,568.7<br>742.5,805.6,1041.7<br>1216.1,1309.1,1350.5<br>1394.9,1582.8,2029.6<br>2965.8,3040.4,3837.5	0.0177
**64**	TS_CH₂OHOH_b → H₂O+HCOH_Z	ZPE(ωB97X-D/def2-TZVP)=0.0501071<br>E(ωB97X-D/def2-TZVP)=−190.8534352<br>E(UCCSD(T)/aug-cc-pVDZ)=−190.4213798<br>E(UCCSD(T)/aug-cc-pVTZ)=−190.5875157	C<br>H<br>H<br>O<br>O<br>H<br>H	−0.041127<br>−0.430530<br>0.981205<br>0.155205<br>−0.337383<br>−1.240422<br>0.913051	0.577053<br>−0.589784<br>0.819731<br>−1.409635<br>1.266660<br>1.074120<br>−1.738145	0.472607<br>0.656051<br>0.772770<br>−0.061280<br>−0.594188<br>−0.868184<br>0.431023	−1320.2,256.8,344.3<br>378.2,725.8,778.0<br>1057.6,1204.2,1341.4<br>1458.6,1547.9,2097.5<br>3111.1,3823.9,3869.3	0.0231

**65**	TS_ CH_2OO+H→ CH_3OO	ZPE(ωB97X-D/def2-TZVP)=0.0316770<br>E(ωB97X-D/def2-TZVP)=−190.0956629<br>E(UCCSD(T)/aug-cc-pVDZ)=−189.6691258<br>E(UCCSD(T)/aug-cc-pVTZ)=−189.8313224	C<br>H<br>H<br>H<br>O<br>O	0.761241<br>2.508240<br>1.386728<br>0.952340<br>−0.283180<br>−1.108019	−0.699272<br>1.294155<br>−0.969214<br>−0.954808<br>−0.068505<br>0.286233	−0.190577<br>0.349427<br>−1.029813<br>0.845259<br>−0.480533<br>0.493606	−307.6,96.5,154.3<br>548.5,686.5,918.7<br>995.7,1256.2,1300.5<br>1499.4,3144.6,3303.9	0.0442
**66**	TS_ CH_2OO→CHOOH_Z	ZPE(ωB97X-D/def2-TZVP)=0.0267039<br>E(ωB97X-D/def2-TZVP)=−189.5391499<br>E(UCCSD(T)/aug-cc-pVDZ)=−189.1167837<br>E(UCCSD(T)/aug-cc-pVTZ)=−189.2774094	C<br>H<br>H<br>O<br>O	−1.029345<br>−1.671817<br>−0.502013<br>−0.000399<br>0.620780	−0.781970<br>−1.198122<br>−0.404436<br>−0.229321<br>0.203751	0.138728<br>0.924138<br>−1.040693<br>0.573615<br>−0.642458	−1781.2,740.8,748.9<br>937.6,1185.3,1284.6<br>1586.1,2160.0,3078.4	0.0315
**67**	TS_ CH_2OOH→ CH_2OO+H	ZPE(ωB97X-D/def2-TZVP)=0.0322743<br>E(ωB97X-D/def2-TZVP)=−190.0949336<br>E(UCCSD(T)/aug-cc-pVDZ)=−189.6693032<br>E(UCCSD(T)/aug-cc-pVTZ)=−189.8318724	C<br>H<br>H<br>O<br>O<br>H	−0.925437<br>−1.912838<br>−0.634397<br>1.162798<br>−0.078213<br>1.580064	0.182019<br>−0.257305<br>1.011577<br>0.151331<br>−0.328107<br>−0.206886	0.092570<br>0.048486<br>0.727052<br>−0.656541<br>−0.670459<br>1.551143	−317.1,178.0,203.1<br>550.6,683.5,954.7<br>980.3,1255.9,1383.5<br>1536.1,3142.7,3298.4	0.0433
**68**	TS_ CH_2OOH→ CH_2[OH]O	ZPE(ωB97X-D/def2-TZVP)=0.0399349<br>E(ωB97X-D/def2-TZVP)=−190.0896336<br>E(UCCSD(T)/aug-cc-pVDZ)=−189.6629965<br>E(UCCSD(T)/aug-cc-pVTZ)=−189.8261850	C<br>H<br>H<br>O<br>O<br>H	−0.798029<br>−1.335795<br>−1.239737<br>0.781889<br>−0.041295<br>1.126568	0.545677<br>−0.178346<br>1.193866<br>0.290885<br>−0.649133<br>−0.235509	−0.515348<br>−1.108598<br>0.220739<br>−0.195765<br>0.568285<br>−0.931554	−1002.4,432.5,662.0<br>829.0,834.0,936.0<br>1058.3,1198.4,1317.7<br>3150.8,3351.9,3758.8	0.0311
**69**	TS_ CH_2OOH→ $CH_2(^3B_1)$+ HO_2	ZPE(ωB97X-D/def2-TZVP)=0.0329826<br>E(ωB97X-D/def2-TZVP)=−190.0767178<br>E(UCCSD(T)/aug-cc-pVDZ)=−189.6121678<br>E(UCCSD(T)/aug-cc-pVTZ)=−189.7733327	C<br>H<br>H<br>O<br>O<br>H	−2.186120<br>−2.550456<br>−1.462302<br>1.571441<br>1.028291<br>2.037613	0.056442<br>0.271115<br>0.518087<br>−0.229073<br>0.810455<br>−0.711584	−0.036975<br>−1.030788<br>0.619023<br>0.786373<br>0.204521<br>0.082984	−53.8,37.9,49.9<br>107.9,116.3,206.1<br>1076.6,1243.9,1466.2<br>3133.9,3365.5,3673.4	0.0260

**70**	TS_CH_2OOH→ $CHOOH$_Z+H	ZPE(ωB97X-D/def2-TZVP)=0.0300073<br>E(ωB97X-D/def2-TZVP)=−190.0765568<br>E(UCCSD(T)/aug-cc-pVDZ)=−189.6545467<br>E(UCCSD(T)/aug-cc-pVTZ)=−189.8147032	C<br>H<br>H<br>O<br>O<br>H	−1.094923<br>−1.896236<br>−1.712121<br>1.150397<br>−0.094161<br>1.789067	−0.039317<br>−0.665694<br>2.426145<br>0.270662<br>−0.449415<br>−0.167001	−0.333501<br>0.107551<br>0.589214<br>−0.167847<br>0.273419<br>0.410940	−280.7,73.7,109.5<br>218.5,499.7,592.8<br>867.7,1239.9,1351.0<br>1460.6,2951.7,3806.6	0.0410
**71**	TS_$CH_2[OH]O$→ CH_2OO+H	ZPE(ωB97X-D/def2-TZVP)=0.0325095<br>E(ωB97X-D/def2-TZVP)=−190.0674833<br>E(UCCSD(T)/aug-cc-pVDZ)=−189.6360453<br>E(UCCSD(T)/aug-cc-pVTZ)=−189.7984894	C<br>H<br>H<br>O<br>O<br>H	−0.801083<br>−0.899215<br>−1.588093<br>0.383749<br>0.522592<br>0.875655	0.554551<br>0.770846<br>0.542016<br>0.158876<br>−0.136247<br>−0.922607	−0.538246<br>−1.591104<br>0.200198<br>−0.164556<br>1.140107<br>−1.008646	−1914.3,457.7,516.4<br>603.1,699.1,724.4<br>934.2,1157.4,1212.7<br>1457.4,3171.5,3336.2	0.0426
**72**	TS_$CH_2[OH]O$→ $CH_2(^3B_1)$+HO_2	ZPE(ωB97X-D/def2-TZVP)=0.0355735<br>E(ωB97X-D/def2-TZVP)=−190.0670171<br>E(UCCSD(T)/aug-cc-pVDZ)=−189.6435655<br>E(UCCSD(T)/aug-cc-pVTZ)=−189.8026676	C<br>H<br>H<br>O<br>O<br>H	−0.919706<br>−1.547081<br>−1.117496<br>0.441238<br>0.474258<br>1.162389	0.642178<br>−0.215745<br>1.704445<br>−0.023411<br>−1.337746<br>0.197720	−0.888711<br>−1.076909<br>−0.870635<br>0.447200<br>0.595459<br>−0.168643	−493.1,114.9,223.4<br>313.8,564.8,649.3<br>1092.3,1133.3,1386.1<br>3134.7,3363.3,3639.0	0.0646
**73**	TS_$CH_2[OH]O$→ HCOHOH	ZPE(ωB97X-D/def2-TZVP)=0.0360425<br>E(ωB97X-D/def2-TZVP)=−190.0680720<br>E(UCCSD(T)/aug-cc-pVDZ)=−189.6434090<br>E(UCCSD(T)/aug-cc-pVTZ)=−189.8053229	C<br>H<br>H<br>O<br>O<br>H	−0.928358<br>−0.956341<br>−1.308479<br>0.226470<br>−0.130473<br>1.055819	0.431096<br>−0.915073<br>1.440754<br>0.239505<br>−1.142335<br>0.164890	−1.026705<br>−0.555804<br>−0.823397<br>0.003897<br>0.270323<br>−0.491409	−1395.9,173.6,608.6<br>811.5,872.9,920.3<br>1026.8,1211.3,1247.7<br>2131.3,3062.1,3754.6	0.0211
**74**	TS_CH_3+$O_2(^3\Sigma g^-)$ → CH_3OO	ZPE(ωB97X-D/def2-TZVP)=0.0366131<br>E(ωB97X-D/def2-TZVP)=−190.1775857<br>E(UCCSD(T)/aug-cc-pVDZ)=−189.7441058<br>E(UCCSD(T)/aug-cc-pVTZ)=−189.9041557	C<br>H<br>H<br>H<br>O<br>O	−1.107882<br>−2.090808<br>−0.699249<br>−0.699249<br>0.084778<br>1.283587	−1.161403<br>−0.714394<br>−1.526576<br>−1.526576<br>0.795139<br>0.656041	0.000000<br>0.000000<br>0.930304<br>−0.930304<br>0.000000<br>0.000000	−199.7,52.8,223.3<br>429.0,463.0,757.4<br>1415.9,1416.4,1579.6<br>3124.6,3304.4,3304.9	0.0944

**75**	TS_ CH_3O→HCHO +H	ZPE(ωB97X-D/def2-TZVP)=0.0284018<br>E(ωB97X-D/def2-TZVP)=−115.0064856<br>E(UCCSD(T)/aug-cc-pVDZ)=−114.7365737<br>E(UCCSD(T)/aug-cc-pVTZ)=−114.8363961	C<br>H<br>H<br>H<br>O	0.150350<br>1.083688<br>−0.702763<br>−0.483693<br>−0.081208	1.647284<br>2.149440<br>2.641817<br>2.206232<br>0.496066	−0.242788<br>0.073899<br>1.109959<br>−0.956327<br>0.046042	−851.4,417.4,479.3<br>1179.2,1259.0,1519.2<br>1738.9,2907.7,2966.2	0.0350
**76**	TS_ CH_3OH→ $CH_2(^1A_1)$+ H_2O	ZPE(ωB97X-D/def2-TZVP)=0.0416743<br>E(ωB97X-D/def2-TZVP)=−115.5785615<br>E(UCCSD(T)/aug-cc-pVDZ)=−115.3128864<br>E(UCCSD(T)/aug-cc-pVTZ)=−115.4149419	C<br>H<br>H<br>O<br>H<br>H	−0.307609<br>−0.062612<br>−0.797718<br>1.279957<br>1.783034<br>0.607047	0.434705<br>1.151105<br>−0.401754<br>−0.400493<br>0.332013<br>0.004291	0.491698<br>−0.314203<br>−0.039775<br>2.846594<br>3.202503<br>2.274267	−129.8,127.1,158.2<br>250.3,387.2,730.4<br>1401.6,1649.4,2970.6<br>3050.0,3630.2,3938.0	0.0125
**77**	TS_ CH_3OH→HCHO+ H_2	ZPE(ωB97X-D/def2-TZVP)=0.0426495<br>E(ωB97X-D/def2-TZVP)=−115.5839292<br>E(UCCSD(T)/aug-cc-pVDZ)=−115.3025848<br>E(UCCSD(T)/aug-cc-pVTZ)=−115.4092216	H<br>H<br>H<br>H<br>C<br>O	1.039867<br>1.039867<br>1.105816<br>0.133459<br>0.523606<br>−0.787233	−0.494294<br>−0.494294<br>1.135347<br>1.048934<br>−0.158353<br>−0.029767	−0.914901<br>0.914901<br>0.000000<br>0.000000<br>−0.000000<br>−0.000000	−2170.7,866.1,916.9<br>1161.3,1190.1,1288.6<br>1481.0,1509.0,2044.0<br>2331.9,2944.8,2987.2	0.0179
**78**	TS_ CH_3OH→ HCOH_Z+ H_2	ZPE(ωB97X-D/def2-TZVP)=0.0422619<br>E(ωB97X-D/def2-TZVP)=−115.5901294<br>E(UCCSD(T)/aug-cc-pVDZ)=−115.3116876<br>E(UCCSD(T)/aug-cc-pVTZ)=−115.4165975	O<br>H<br>C<br>H<br>H<br>H	−1.353266<br>−1.926942<br>−0.087502<br>0.547346<br>0.415122<br>0.334265	0.196422<br>−0.319434<br>−0.097838<br>0.599771<br>−1.189592<br>−1.319945	0.299622<br>−0.272986<br>0.001756<br>0.572476<br>1.318318<br>0.481226	−875.2,601.7,688.5<br>976.7,1046.5,1214.1<br>1290.2,1450.4,1526.2<br>2900.6,3010.9,3845.0	0.0185
**79**	TS_ CH_3OHO→ CH_4+ $O_2(^1\Delta g)$	ZPE(ωB97X-D/def2-TZVP)=0.0475726<br>E(ωB97X-D/def2-TZVP)=−190.7245256<br>E(UCCSD(T)/aug-cc-pVDZ)=−190.3004763<br>E(UCCSD(T)/aug-cc-pVTZ)=−190.4670257	C<br>H<br>H<br>H<br>O<br>O<br>H	−1.142013<br>−1.354990<br>−0.784962<br>−1.858083<br>0.788864<br>0.323838<br>0.273705	−0.376989<br>−0.373501<br>−1.312064<br>0.148192<br>0.613250<br>0.952401<br>0.131988	0.413695<br>1.479725<br>−0.012225<br>−0.199719<br>−1.334779<br>−0.180898<br>0.489568	−1352.5,84.9,228.1<br>454.5,759.0,805.1<br>1110.0,1228.4,1302.2<br>1401.9,1458.9,2466.3<br>3078.2,3224.8,3279.7	0.0581

**80**	TS_CH_3OHO→H+ CH_3OO	ZPE(ωB97X-D/def2-TZVP)=0.0459949<br>E(ωB97X-D/def2-TZVP)=−190.7258594<br>E(UCCSD(T)/aug-cc-pVDZ)=−190.2952969<br>E(UCCSD(T)/aug-cc-pVTZ)=−190.4606598	C<br>H<br>H<br>H<br>O<br>O<br>H	−0.023142<br>−0.286298<br>−0.273065<br>−0.481698<br>1.418687<br>1.828974<br>1.581008	0.071127<br>−0.929922<br>0.220545<br>0.835121<br>0.163430<br>1.361214<br>−0.317209	−0.033281<br>0.301711<br>−1.081194<br>0.589714<br>0.079427<br>−0.227417<br>1.665091	−1041.1,216.3,496.2<br>507.8,581.8,931.3<br>1158.6,1173.8,1252.3<br>1450.3,1472.0,1492.1<br>3079.5,3186.8,3190.5	0.0453
**81**	TS_CH_3OHO→ H_2+CH[OH]O_E	ZPE(ωB97X-D/def2-TZVP)=0.0443635<br>E(ωB97X-D/def2-TZVP)=−190.6328103<br>E(UCCSD(T)/aug-cc-pVDZ)=−190.1977290<br>E(UCCSD(T)/aug-cc-pVTZ)=−190.3655681	C<br>H<br>H<br>H<br>O<br>O<br>H	1.253816<br>1.119703<br>0.163329<br>1.688373<br>1.387930<br>2.637678<br>0.665698	0.002134<br>0.016788<br>−0.177854<br>−0.255113<br>1.320388<br>2.004419<br>1.948053	−0.180249<br>1.709708<br>−0.172182<br>1.196036<br>−0.376820<br>−0.196647<br>−0.229600	−686.7,219.2,459.2<br>578.6,620.3,762.8<br>809.6,950.7,1162.4<br>1207.1,1259.0,1402.9<br>2992.7,3335.0,3713.8	0.0269
**82**	TS_CH_3OHO→ H_2O+HCHO	ZPE(ωB97X-D/def2-TZVP)=0.0498830<br>E(ωB97X-D/def2-TZVP)=−190.7392511<br>E(UCCSD(T)/aug-cc-pVDZ)=−190.3061964<br>E(UCCSD(T)/aug-cc-pVTZ)=−190.4746506	C<br>H<br>H<br>H<br>O<br>O<br>H	−1.235799<br>−0.131088<br>−1.472093<br>−1.952024<br>0.865349<br>0.107087<br>0.068244	−0.253166<br>−1.143483<br>−0.174855<br>0.323048<br>−0.607567<br>0.503046<br>1.132149	−0.159329<br>0.414095<br>−1.220064<br>0.431670<br>0.527701<br>−0.034840<br>0.698298	−1186.9,281.4,581.4<br>843.0,875.6,913.6<br>1141.3,1161.4,1198.1<br>1245.0,1459.1,2174.8<br>3070.3,3167.6,3783.5	0.0183
**83**	TS_CH_3OO→CH_2 OOH	ZPE(ωB97X-D/def2-TZVP)=0.0366048<br>E(ωB97X-D/def2-TZVP)=−190.1513187<br>E(UCCSD(T)/aug-cc-pVDZ)=−189.7194053<br>E(UCCSD(T)/aug-cc-pVTZ)=−189.8834467	C<br>H<br>H<br>O<br>O<br>H	−0.816705<br>−1.560642<br>−0.874112<br>0.867057<br>−0.426766<br>0.387164	0.311910<br>0.897018<br>0.266214<br>−0.227606<br>−0.852627<br>0.773097	−0.207012<br>0.331890<br>−1.293686<br>0.713454<br>0.409945<br>0.121657	−1924.4,220.6,775.0<br>867.5,1015.8,1100.0<br>1146.3,1175.1,1474.2<br>2027.7,3073.7,3191.8	0.0283
**84**	TS_CH_3OO→CH_2[OH]O	ZPE(ωB97X-D/def2-TZVP)=0.0364174<br>E(ωB97X-D/def2-TZVP)=−190.0876874<br>E(UCCSD(T)/aug-cc-pVDZ)=−189.6528678<br>E(UCCSD(T)/aug-cc-pVTZ)=−189.8176519	C<br>H<br>H<br>O<br>O<br>H	1.312958<br>1.526444<br>1.799241<br>−0.891417<br>−0.109682<br>0.434537	0.075810<br>−0.620111<br>0.050749<br>−0.343578<br>0.321134<br>1.197161	−0.025668<br>0.774166<br>−0.992701<br>0.738044<br>−0.157650<br>0.237723	−2079.5,342.9,460.1<br>784.6,864.5,996.9<br>1104.1,1163.1,1387.2<br>2435.9,3141.9,3304.1	0.0399

**85**	TS_CH_3OOH→ CH_3OHO_HighBarrier	ZPE(ωB97X-D/def2-TZVP)=0.0528861<br>E(ωB97X-D/def2-TZVP)=−190.7386029<br>E(UCCSD(T)/aug-cc-pVDZ)=−190.3096590<br>E(UCCSD(T)/aug-cc-pVTZ)=−190.4765535	C<br>H<br>H<br>H<br>O<br>O<br>H	0.799890<br>1.456664<br>1.073641<br>0.673504<br>−0.247753<br>−0.952025<br>−1.230004	−0.494256<br>0.191871<br>−0.670148<br>−1.429981<br>0.997797<br>−0.239343<br>0.020942	0.028511<br>0.547886<br>−1.000492<br>0.580899<br>−0.449918<br>0.028890<br>0.915676	−935.3,155.1,586.0<br>716.2,894.9,1000.4<br>1084.6,1153.8,1374.0<br>1462.8,1499.7,3028.0<br>3178.7,3278.1,3802.0	0.0288
**86**	TS_CH_3OOH→ CH_3OHO_LowBarrier	ZPE(ωB97X-D/def2-TZVP)=0.0515350<br>E(ωB97X-D/def2-TZVP)=−190.7868377<br>E(UCCSD(T)/aug-cc-pVDZ)=−190.3552817<br>E(UCCSD(T)/aug-cc-pVTZ)=−190.5242168	C<br>H<br>H<br>H<br>O<br>O<br>H	1.100122<br>1.899607<br>1.212912<br>1.063526<br>−0.117571<br>−1.284615<br>−0.736792	−0.211590<br>0.522682<br>−0.786519<br>−0.879796<br>0.522969<br>−0.507100<br>0.431428	0.050487<br>0.150915<br>−0.865906<br>0.909204<br>−0.060341<br>−0.038925<br>0.766673	−1268.3,197.1,375.5<br>732.6,971.8,1037.4<br>1196.9,1249.6,1463.0<br>1474.0,1504.6,3005.2<br>3070.7,3165.3,3177.5	0.0250
**87**	TS_CH_3OOH→ H_2+CH_2OO	ZPE(ωB97X-D/def2-TZVP)=0.0475086<br>E(ωB97X-D/def2-TZVP)=−190.7562677<br>E(UCCSD(T)/aug-cc-pVDZ)=−190.3192237<br>E(UCCSD(T)/aug-cc-pVTZ)=−190.4876046	C<br>H<br>H<br>H<br>O<br>O<br>H	0.118914<br>−0.082012<br>−0.637444<br>0.108888<br>1.217886<br>1.988898<br>0.768578	−0.130321<br>1.523753<br>−0.484187<br>−0.419261<br>0.217788<br>0.886816<br>1.661767	0.273387<br>−0.323858<br>0.965573<br>−0.769875<br>0.825456<br>−0.058721<br>−0.423081	−1079.1,559.9,694.2<br>798.6,888.5,1017.4<br>1122.9,1183.0,1232.2<br>1378.4,1411.0,1546.2<br>2602.8,3143.7,3275.0	0.0369
**88**	TS_CH_3OOH→ H_2+CHOOH_E	ZPE(ωB97X-D/def2-TZVP)=0.0449805<br>E(ωB97X-D/def2-TZVP)=−190.7395169<br>E(UCCSD(T)/aug-cc-pVDZ)=−190.3049908<br>E(UCCSD(T)/aug-cc-pVTZ)=−190.4719124	C<br>H<br>H<br>H<br>O<br>O<br>H	1.196850<br>1.970213<br>1.636955<br>1.287319<br>0.032747<br>−0.990501<br>−1.806046	0.092207<br>0.373693<br>−0.232188<br>−0.984396<br>0.576970<br>−0.471384<br>0.027466	0.635240<br>−0.436577<br>−0.935541<br>0.796844<br>0.524899<br>0.054561<br>0.185904	−1019.8,195.2,350.9<br>421.3,566.8,789.2<br>1012.6,1020.8,1232.1<br>1275.2,1406.7,1575.2<br>2956.7,3113.8,3827.7	0.0254
**89**	TS_CH_3OOH→ H_2O+HCHO_High Barrier	ZPE(ωB97X-D/def2-TZVP)=0.0478432<br>E(ωB97X-D/def2-TZVP)=−190.7458251<br>E(UCCSD(T)/aug-cc-pVDZ)=−190.3277568<br>E(UCCSD(T)/aug-cc-pVTZ)=−190.4940059	C<br>H<br>H<br>H<br>O<br>O<br>H	1.058924<br>1.581589<br>1.161110<br>0.956438<br>0.550164<br>−1.222060<br>−1.507862	−0.760791<br>0.226621<br>−1.619773<br>−0.923240<br>0.357272<br>0.206071<br>1.101532	0.481965<br>−0.404181<br>−0.178354<br>1.551364<br>−0.030957<br>−0.213109<br>0.000028	−1548.7,221.1,282.5<br>425.0,524.4,871.2<br>953.6,998.5,1194.2<br>1315.6,1524.8,2530.0<br>3095.6,3220.6,3843.6	0.0299

**90**	TS_CH_3OOH→ H_2O+HCHO_Low Barrier	ZPE(ωB97X-D/def2-TZVP)=0.0476912<br>E(ωB97X-D/def2-TZVP)=−190.7795514<br>E(UCCSD(T)/aug-cc-pVDZ)=−190.3561546<br>E(UCCSD(T)/aug-cc-pVTZ)=−190.5206633	C<br>H<br>H<br>H<br>O<br>O<br>H	0.962588<br>1.895905<br>0.173856<br>0.966339<br>0.225504<br>−0.981640<br>−1.829031	−0.152059<br>0.195667<br>−0.273434<br>−1.144818<br>0.750654<br>0.601349<br>0.978653	0.148426<br>−0.311417<br>−0.818392<br>0.615214<br>0.715049<br>−0.713214<br>−1.013333	−1695.3,249.6,476.9<br>532.2,633.6,1067.3<br>1091.1,1239.3,1252.1<br>1322.4,1521.4,1794.7<br>2987.6,3085.2,3680.8	0.0249
**91**	TS_CHOHOH_Z<br>→<br>CH_2(OH)O	ZPE(ωB97X-D/def2-TZVP)=0.0390011<br>E(ωB97X-D/def2-TZVP)=−190.2555364<br>E(UCCSD(T)/aug-cc-pVDZ)=−189.8197097<br>E(UCCSD(T)/aug-cc-pVTZ)=−189.9851689	C<br>H<br>H<br>O<br>O<br>H	0.826400<br>0.537715<br>−0.032068<br>1.962009<br>0.892402<br>2.704722	1.100660<br>−0.070509<br>1.760045<br>1.797933<br>−0.059897<br>1.195757	0.133449<br>0.586272<br>−0.001892<br>0.343248<br>−0.569162<br>0.226350	−1919.3,434.9,558.2<br>749.4,1029.7,1103.9<br>1241.5,1297.1,1418.8<br>2336.9,3109.8,3839.3	0.0215
**92**	TS_CHOHOH_Z<br>→<br>COHOH_Z+H	ZPE(ωB97X-D/def2-TZVP)=0.0339686<br>E(ωB97X-D/def2-TZVP)=−190.2155330<br>E(UCCSD(T)/aug-cc-pVDZ)=−189.7887616<br>E(UCCSD(T)/aug-cc-pVTZ)=−189.9509275	C<br>H<br>H<br>O<br>O<br>H	−0.203265<br>−1.067006<br>−1.966769<br>0.999281<br>−0.177731<br>1.619626	0.191834<br>−1.349831<br>1.661526<br>0.690889<br>−0.987460<br>0.095037	−0.630336<br>−0.047680<br>0.601816<br>−0.589399<br>−0.020357<br>−0.128343	−333.5,81.6,117.6<br>639.6,665.4,781.4<br>1148.7,1185.5,1362.9<br>1435.1,3611.0,3881.7	0.0226
**93**	TS_CHOHOH_Z<br>→<br>H+HCOOH	ZPE(ωB97X-D/def2-TZVP)=0.0356215<br>E(ωB97X-D/def2-TZVP)=−190.2641780<br>E(UCCSD(T)/aug-cc-pVDZ)=−189.8312758<br>E(UCCSD(T)/aug-cc-pVTZ)=−189.9955527	C<br>H<br>O<br>O<br>H<br>H	−0.017761<br>0.067991<br>1.162759<br>−1.085140<br>1.018400<br>−1.604921	0.461028<br>1.513100<br>−0.159261<br>−0.127820<br>−1.086188<br>−0.881733	0.010174<br>0.281601<br>0.028281<br>−0.160238<br>−0.199003<br>0.913363	−1617.9,257.1,577.8<br>609.2,725.9,961.3<br>1156.9,1328.2,1380.7<br>1681.2,3142.1,3815.6	0.0255
**94**	TS_CHOHOH_Z<br>→<br>H_2O+COH	ZPE(ωB97X-D/def2-TZVP)=0.0365437<br>E(ωB97X-D/def2-TZVP)=−190.1892272<br>E(UCCSD(T)/aug-cc-pVDZ)=−189.7617338<br>E(UCCSD(T)/aug-cc-pVTZ)=−189.9230250	C<br>H<br>H<br>O<br>O<br>H	−0.914175<br>−0.775925<br>−0.440176<br>0.719942<br>−0.669176<br>1.283642	0.149628<br>−1.724699<br>1.185730<br>1.058806<br>−0.973444<br>0.605978	−0.381059<br>−0.367066<br>0.050538<br>−0.493247<br>0.229928<br>0.146604	−1487.8,281.7,303.7<br>499.0,604.6,785.0<br>1142.0,1248.2,1322.9<br>2259.3,3776.8,3817.6	0.0335

**95**	TS_CHOHOH_Z<br>→<br>H_2O+HCO	ZPE(ωB97X-D/def2-TZVP)=0.0384950<br>E(ωB97X-D/def2-TZVP)=−190.2415878<br>E(UCCSD(T)/aug-cc-pVDZ)=−189.8096634<br>E(UCCSD(T)/aug-cc-pVTZ)=−189.9746654	C<br>H<br>H<br>O<br>O<br>H	1.603874<br>0.079890<br>2.078864<br>0.300188<br>1.044563<br>0.399948	1.043897<br>0.662947<br>1.659180<br>1.794864<br>−0.081914<br>1.996164	−0.013219<br>0.297669<br>−0.785977<br>0.495574<br>−0.245820<br>1.432966	−1703.0,552.9,645.1<br>771.1,895.6,1078.4<br>1161.8,1347.8,1505.5<br>2066.5,3034.0,3838.9	0.0208
**96**	TS_CHOOH_E→<br>H_2O+CO	ZPE(ωB97X-D/def2-TZVP)=0.0273074<br>E(ωB97X-D/def2-TZVP)=−189.5868541<br>E(UCCSD(T)/aug-cc-pVDZ)=−189.1849904<br>E(UCCSD(T)/aug-cc-pVTZ)=−189.3413474	C<br>H<br>H<br>O<br>O	2.231690<br>−0.507708<br>1.618379<br>0.116605<br>1.936030	0.760043<br>0.138552<br>1.600810<br>0.571265<br>−0.050069	0.722011<br>−0.954294<br>1.096401<br>−0.360234<br>−0.030615	−329.1,254.8,415.8<br>810.5,818.7,871.5<br>2009.6,2971.5,3834.1	0.0330
**97**	TS_COHOH_E→C<br>O_2+H_2	ZPE(ωB97X-D/def2-TZVP)=0.0239311<br>E(ωB97X-D/def2-TZVP)=−189.6458142<br>E(UCCSD(T)/aug-cc-pVDZ)=−189.2217443<br>E(UCCSD(T)/aug-cc-pVTZ)=−189.3828427	C<br>O<br>O<br>H<br>H	0.001717<br>−1.067063<br>1.068523<br>−0.540093<br>0.536915	−0.610722<br>−0.028844<br>−0.024570<br>1.189300<br>1.189711	0.000040<br>−0.000002<br>−0.000002<br>−0.000091<br>−0.000091	−1943.9,794.8,818.4<br>997.2,1205.7,1419.0<br>1486.3,1881.4,1901.7	0.0209
**98**	TS_COHOH_Z→<br>H_2O+CO	ZPE(ωB97X-D/def2-TZVP)=0.0264376<br>E(ωB97X-D/def2-TZVP)=−189.6527581<br>E(UCCSD(T)/aug-cc-pVDZ)=−189.2331159<br>E(UCCSD(T)/aug-cc-pVTZ)=−189.3931549	C<br>H<br>O<br>O<br>H	1.285036<br>0.562407<br>−0.239793<br>1.742037<br>−0.796426	0.366157<br>1.302817<br>0.730838<br>1.135327<br>−0.029457	−0.492809<br>0.638283<br>−0.142103<br>0.352542<br>0.062911	−1832.4,496.7,596.2<br>813.0,957.5,1262.0<br>1633.2,2011.8,3834.4	0.0199
**99**	TS_H+cCH_2OO→<br>CH_2(OH)O	ZPE(ωB97X-D/def2-TZVP)=0.0336917<br>E(ωB97X-D/def2-TZVP)=−190.1297662<br>E(UCCSD(T)/aug-cc-pVDZ)=−189.7030482<br>E(UCCSD(T)/aug-cc-pVTZ)=−189.8672964	C<br>H<br>H<br>O<br>O<br>H	−0.683401<br>−1.292778<br>−0.025114<br>−0.154235<br>−1.274897<br>1.348711	0.496690<br>0.576072<br>1.343122<br>−0.752586<br>−0.090069<br>−1.032819	0.006548<br>−0.895017<br>0.209203<br>0.268020<br>1.093425<br>−0.542853	−1044.5,110.0,329.9<br>741.3,954.9,1054.3<br>1191.9,1296.8,1334.5<br>1562.7,3061.5,3151.2	0.0468

**100**	TS_H_2+COH→CH_2OH	ZPE(ωB97X-D/def2-TZVP)=0.0281175<br>E(ωB97X-D/def2-TZVP)=−114.9298352<br>E(UCCSD(T)/aug-cc-pVDZ)=−114.6576226<br>E(UCCSD(T)/aug-cc-pVTZ)=−114.7571909	C<br>H<br>H<br>O<br>H	0.508193<br>1.486556<br>1.548616<br>−0.522407<br>−1.307452	0.220150<br>0.905671<br>0.052114<br>0.108335<br>0.429028	0.528588<br>−0.437157<br>−0.300723<br>−0.321776<br>0.132468	−950.0,478.5,729.3<br>843.0,1098.2,1223.5<br>1310.6,2811.9,3847.1	0.0247
**101**	TS_H_2O+CO→HCOOH	ZPE(ωB97X-D/def2-TZVP)=0.0263039<br>E(ωB97X-D/def2-TZVP)=−189.6657372<br>E(UCCSD(T)/aug-cc-pVDZ)=−189.2449109<br>E(UCCSD(T)/aug-cc-pVTZ)=−189.4039425	C<br>H<br>H<br>O<br>O	−0.931034<br>0.885183<br>0.084380<br>−1.847223<br>0.776899	−0.317485<br>−1.614418<br>−0.334916<br>−0.525782<br>−0.689967	0.020659<br>0.856880<br>−0.546814<br>0.664226<br>0.605800	−1702.7,318.9,382.5<br>573.0,771.0,1065.5<br>1982.0,2616.1,3837.0	0.0220
**102**	TS_H_2O_2→H_2+O_2($^1\Delta$g)	ZPE(ωB97X-D/def2-TZVP)=0.0148975<br>E(ωB97X-D/def2-TZVP)=−151.3406544<br>E(UCCSD(T)/aug-cc-pVDZ)=−151.0986190<br>E(UCCSD(T)/aug-cc-pVTZ)=−151.2262621	O<br>H<br>O<br>H	−0.523162<br>0.331704<br>−0.845317<br>1.036771	−0.703163<br>−0.475226<br>0.462296<br>0.716089	0.041709<br>0.597515<br>−0.358314<br>0.516680	−1646.6,729.0,977.3<br>1162.3,1354.6,2316.0	0.0896
**103**	TS_H_2O_2→H_2OO	ZPE(ωB97X-D/def2-TZVP)=0.0226713<br>E(ωB97X-D/def2-TZVP)=−151.4783790<br>E(UCCSD(T)/aug-cc-pVDZ)=−151.1598913<br>E(UCCSD(T)/aug-cc-pVTZ)=−151.2903557	O<br>H<br>O<br>H	−0.146835<br>−0.595764<br>1.282703<br>0.145406	−0.549247<br>−1.089208<br>−0.399143<br>0.332322	−0.280239<br>0.381894<br>0.388884<br>0.173882	−1180.1,713.2,995.4<br>1391.8,3024.4,3826.7	0.0289
**104**	TS_H_2OO→H_2+O_2($^1\Delta$g)	ZPE(ωB97X-D/def2-TZVP)=0.0146625<br>E(ωB97X-D/def2-TZVP)=−151.3896304<br>E(UCCSD(T)/aug-cc-pVDZ)=−151.0683429<br>E(UCCSD(T)/aug-cc-pVTZ)=−151.1978991	O<br>H<br>O<br>H	0.262145<br>−0.179569<br>−0.113603<br>0.716538	−0.313002<br>−1.334179<br>0.867337<br>−0.925434	−0.352188<br>0.516284<br>−0.054767<br>0.555089	−1849.9,502.2,989.3<br>1200.2,1284.4,2460.1	0.0439

**105**	TS_HCHO+H→HCO+H_2	ZPE(ωB97X-D/def2-TZVP)=0.0246489<br>E(ωB97X-D/def2-TZVP)=−115.0054895<br>E(UCCSD(T)/aug-cc-pVDZ)=−114.7325299<br>E(UCCSD(T)/aug-cc-pVTZ)=−114.8316523	C<br>H<br>O<br>H<br>H	−0.282747<br>−0.620206<br>−1.014537<br>0.928905<br>2.071635	0.290807<br>1.348358<br>−0.637025<br>0.152648<br>0.018620	0.000000<br>0.000000<br>0.000000<br>0.000000<br>0.000000	−1100.8,322.5,408.5<br>1208.2,1219.7,1403.3<br>1455.2,1904.5,2897.7	0.0238
**106**	TS_HCHO→CO+H_2	ZPE(ωB97X-D/def2-TZVP)=0.0188107<br>E(ωB97X-D/def2-TZVP)=−114.3719378<br>E(UCCSD(T)/aug-cc-pVDZ)=−114.1069622<br>E(UCCSD(T)/aug-cc-pVTZ)=−114.2043065	C<br>H<br>H<br>O	−0.000000<br>0.000000<br>0.000000<br>0.000000	0.379997<br>−0.798291<br>0.469293<br>−0.051000	−0.225980<br>−1.372011<br>−1.322994<br>0.846841	−1970.8,815.0,939.2<br>1400.4,1956.8,3145.6	0.0212
**107**	TS_HCHO→HCOH_Z	ZPE(ωB97X-D/def2-TZVP)=0.0208704<br>E(ωB97X-D/def2-TZVP)=−114.3727751<br>E(UCCSD(T)/aug-cc-pVDZ)=−114.1070406<br>E(UCCSD(T)/aug-cc-pVTZ)=−114.2060492	C<br>H<br>O<br>H	−0.742477<br>−0.093695<br>−1.504867<br>−1.545630	0.014221<br>−0.202075<br>−0.961843<br>0.043134	0.031521<br>0.907232<br>−0.329312<br>−0.922823	−2166.3,746.9,1341.3<br>1483.2,2672.3,2917.3	0.0244
**108**	TS_HCO+H_2O→$CH_2(OH)O$	ZPE(ωB97X-D/def2-TZVP)=0.0383290<br>E(ωB97X-D/def2-TZVP)=−190.2520375<br>E(UCCSD(T)/aug-cc-pVDZ)=−189.8203069<br>E(UCCSD(T)/aug-cc-pVTZ)=−189.9813338	C<br>H<br>H<br>O<br>O<br>H	−0.015961<br>−0.888677<br>−1.007749<br>1.031140<br>−1.224472<br>−0.141595	0.478220<br>−1.310459<br>0.622753<br>0.492128<br>−1.000672<br>0.527427	−0.581473<br>0.605702<br>−0.015564<br>−0.004060<br>−0.248606<br>−1.672027	−344.4,328.5,435.5<br>558.6,839.6,1009.0<br>1159.6,1398.3,1764.5<br>2513.7,3026.3,3790.8	0.0426
**109**	TS_$HCOH_2$→HCOH_Z+H	ZPE(ωB97X-D/def2-TZVP)=0.0295833<br>E(ωB97X-D/def2-TZVP)=−114.8959309<br>E(UCCSD(T)/aug-cc-pVDZ)=−114.6274261<br>E(UCCSD(T)/aug-cc-pVTZ)=−114.7263091	C<br>H<br>H<br>O<br>H	0.521251<br>0.931738<br>−0.449468<br>−0.531692<br>−0.940714	0.529799<br>1.048799<br>−1.113278<br>−0.174172<br>−0.678669	−0.729526<br>0.156033<br>0.726952<br>−0.205267<br>−0.920509	−2130.2,679.9,781.9<br>958.2,1154.2,1250.0<br>1419.0,2959.9,3782.5	0.0666

**110**	TS_HCOH_E→CO +H_2	ZPE(ωB97X-D/def2-TZVP)=0.0176975<br>E(ωB97X-D/def2-TZVP)=−114.3349295<br>E(UCCSD(T)/aug-cc-pVDZ)=−114.0723880<br>E(UCCSD(T)/aug-cc-pVTZ)=−114.1695965	C<br>H<br>H<br>O	1.016450<br>0.026859<br>−0.305369<br>1.147703	1.171348<br>0.251779<br>1.241211<br>−0.060586	0.073006<br>0.158562<br>0.415869<br>−0.176796	−2504.4,975.2,1035.5<br>1575.8,1908.9,2272.9	0.0210
**111**	TS_HCOH_Z→CO H_2	ZPE(ωB97X-D/def2-TZVP)=0.0209597<br>E(ωB97X-D/def2-TZVP)=−114.2376187<br>E(UCCSD(T)/aug-cc-pVDZ)=−113.9859903<br>E(UCCSD(T)/aug-cc-pVTZ)=−114.0799722	C<br>H<br>H<br>O	−0.130503<br>0.016240<br>−0.115869<br>0.410476	−1.124534<br>−0.248145<br>1.063301<br>0.342285	1.004037<br>−0.338866<br>0.748737<br>0.383061	−1081.5,324.6,728.4<br>1302.0,3044.1,3801.2	0.0222
**112**	TS_HCOOH→CO HOH_Z	ZPE(ωB97X-D/def2-TZVP)=0.0277247<br>E(ωB97X-D/def2-TZVP)=−189.6572768<br>E(UCCSD(T)/aug-cc-pVDZ)=−189.2290084<br>E(UCCSD(T)/aug-cc-pVTZ)=−189.3920601	C<br>H<br>O<br>O<br>H	0.045867<br>1.219710<br>−1.151646<br>1.047605<br>−1.062264	0.489262<br>0.891152<br>−0.022204<br>−0.313033<br>−0.996238	−0.000158<br>−0.000030<br>−0.000325<br>−0.000409<br>−0.000633	−1968.7,486.2,629.6<br>731.2,1203.5,1330.1<br>1547.0,2645.1,3597.0	0.0215
**113**	TS_HCOOH_E→C O_2+H_2	ZPE(ωB97X-D/def2-TZVP)=0.0245627<br>E(ωB97X-D/def2-TZVP)=−189.6662527<br>E(UCCSD(T)/aug-cc-pVDZ)=−189.2339408<br>E(UCCSD(T)/aug-cc-pVTZ)=−189.3969663	C<br>H<br>O<br>O<br>H	0.176531<br>−0.267843<br>−0.907198<br>1.319243<br>−1.042259	−0.174066<br>1.142865<br>−0.679261<br>−0.327779<br>0.556749	0.027742<br>−0.323386<br>0.391729<br>−0.130699<br>0.010621	−2248.9,626.6,743.0<br>915.8,1058.0,1372.4<br>1797.2,2113.8,2154.9	0.0217
**114**	TS_HO_2→O_2($^3\Sigma g^-$) +H	ZPE(ωB97X-D/def2-TZVP)=0.0047559<br>E(ωB97X-D/def2-TZVP)=−150.8375090<br>E(UCCSD(T)/aug-cc-pVDZ)=−150.5162594<br>E(UCCSD(T)/aug-cc-pVTZ)=−150.6376990	O<br>O<br>H	0.172145<br>0.171133<br>−1.530877	0.746552<br>−0.451699<br>−1.312392	0.000000<br>0.000000<br>0.000000	−631.3,427.3,1660.3	0.0273

**115**	TS_OH+HCHO→<br>$CH_2(OH)O$	ZPE(ωB97X-D/def2-TZVP)=0.0396262<br>E(ωB97X-D/def2-TZVP)=−190.2514023<br>E(UCCSD(T)/aug-cc-pVDZ)=−189.7819248<br>E(UCCSD(T)/aug-cc-pVTZ)=−189.9427770	C<br>H<br>H<br>O<br>O<br>H	−0.763200<br>−0.988226<br>−0.707123<br>1.149376<br>−0.838683<br>1.410165	0.089747<br>−0.814889<br>1.031131<br>−0.286612<br>0.087728<br>0.241247	−0.283541<br>−0.866400<br>−0.848448<br>−0.239100<br>0.943970<br>0.532478	−426.9,244.9,328.2<br>665.3,797.4,1165.4<br>1246.9,1493.7,1648.9<br>2974.9,3053.7,3774.5	0.0440
**116**	MP2_CH_2OOH<br>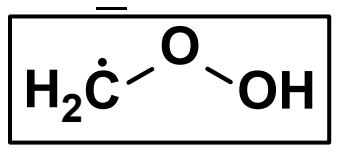	ZPE(MP2/6-31G(d))=0.0417104<br>E(MP2/6-31G(d))=−189.1619221<br>E(UCCSD(T)/aug-cc-pVDZ)=−189.7751437<br>E(UCCSD(T)/aug-cc-pVTZ)=−189.9390499	C<br>H<br>H<br>O<br>O<br>H	−1.955311<br>−1.400713<br>−1.863532<br>−2.648635<br>−1.973230<br>−3.518791	−0.833092<br>−0.313235<br>−0.555448<br>−2.838813<br>−2.179358<br>−2.997663	−0.025267<br>−0.797112<br>1.017990<br>0.818847<br>−0.298897<br>0.401303	200.3,299.8,488.1<br>830.6,919.3,1187.9<br>1226.7,1391.8,1490.3<br>3210.4,3356.8,3706.9	0.0199
**117**	MP2_$CH_2[OH]O$<br>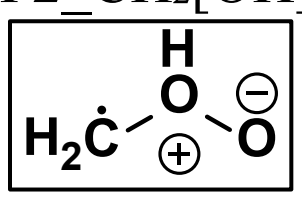	ZPE(MP2/6-31G(d))=0.0419771<br>E(MP2/6-31G(d))=−189.0933136<br>E(UCCSD(T)/aug-cc-pVDZ)=−189.6945453<br>E(UCCSD(T)/aug-cc-pVTZ)=−189.8577282	C<br>H<br>H<br>O<br>O<br>H	−2.104052<br>−2.200304<br>−1.339193<br>−2.213774<br>−2.292567<br>−3.210321	−0.902696<br>−0.378950<br>−0.702413<br>−2.940578<br>−2.296212<br>−2.496760	0.105628<br>−0.837597<br>0.836518<br>1.325747<br>−0.016081<br>−0.297351	255.7,376.9,736.2<br>830.7,890.8,1098.7<br>1134.3,1314.3,1463.1<br>3238.2,3425.8,3661.1	0.0218
**118**	MP2_$CH_2(^1A_1)$<br>: CH_2	ZPE(MP2/6-31G(d))=0.0172757<br>E(MP2/6-31G(d))=−38.8721820<br>E(UCCSD(T)/aug-cc-pVDZ)=−39.0312248<br>E(UCCSD(T)/aug-cc-pVTZ)=−39.0645886	C<br>H<br>H	0.280909<br>0.917566<br>0.962821	0.364352<br>1.077955<br>−0.500722	−0.744989<br>−0.182964<br>−0.875665	1498.5,3000.5,3084.2	0.0098
**119**	MP2_CH_4<br>CH_4	ZPE(MP2/6-31G(d))=0.0463537<br>E(MP2/6-31G(d))=−40.1950596<br>E(UCCSD(T)/aug-cc-pVDZ)=−40.3954170<br>E(UCCSD(T)/aug-cc-pVTZ)=−40.4409300	C<br>H<br>H<br>H<br>H	0.831431<br>1.194767<br>1.194787<br>1.194785<br>−0.258614	2.176925<br>1.149216<br>2.690772<br>2.690773<br>2.176938	0.000000<br>−0.000001<br>0.890018<br>−0.890018<br>0.000001	1413.6,1413.6,1413.6<br>1624.7,1624.7,3111.3<br>3248.5,3248.5,3248.5	0.0068

**120**	MP2_H₂<br>H_2	ZPE(MP2/6-31G(d))=0.0103279<br>E(MP2/6-31G(d))=−1.1267864<br>E(UCCSD(T)/aug-cc-pVDZ)=−1.1645047<br>E(UCCSD(T)/aug-cc-pVTZ)=−1.1726158	H<br>H	−1.857357<br>−2.119952	−0.402217<br>−0.685564	0.861515<br>1.489775	4533.4	0.0046
**121**	MP2_HCHO<br>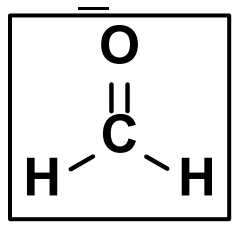	ZPE(MP2/6-31G(d))=0.0272824<br>E(MP2/6-31G(d))=−113.8635619<br>E(UCCSD(T)/aug-cc-pVDZ)=−114.2450234<br>E(UCCSD(T)/aug-cc-pVTZ)=−114.3427538	C<br>H<br>H<br>O	−1.091010<br>−2.099168<br>−1.050021<br>−0.086955	0.176099<br>−0.242109<br>1.263535<br>−0.518653	−0.058406<br>0.108854<br>−0.245643<br>−0.037667	1212.3,1295.8,1583.2<br>1787.6,3012.4,3084.2	0.0172
**122**	MP2_OH<br>$\dot{O}H$	ZPE(MP2/6-31G(d))=0.0085179<br>E(MP2/6-31G(d))=−75.3818600<br>E(UCCSD(T)/aug-cc-pVDZ)=−75.5840785<br>E(UCCSD(T)/aug-cc-pVTZ)=−75.6455564	O<br>H	0.785317<br>0.458490	1.352575<br>2.275505	0.826989<br>0.826989	3738.9	0.0122
**123**	MP2_TS_CH₂OOH<br>→<br>CH₂[OH]O	ZPE(MP2/6-31G(d))=0.0387602<br>E(MP2/6-31G(d))=−189.0673493<br>E(UCCSD(T)/aug-cc-pVDZ)=−189.6826305<br>E(UCCSD(T)/aug-cc-pVTZ)=−189.8461069	C<br>H<br>H<br>O<br>O<br>H	1.209784<br>2.090091<br>1.108302<br>−1.153227<br>0.014255<br>−0.467139	0.185230<br>−0.438438<br>1.189393<br>0.425329<br>−0.555592<br>−0.626384	−0.009764<br>0.038175<br>0.367977<br>0.114561<br>−0.051544<br>0.857335	−1364.9,74.7,412.4<br>520.5,972.8,1056.8<br>1175.7,1457.3,1557.9<br>3100.3,3254.0,3431.3	0.0270
**124**	MP2_TS_CH₂OOH<br>→<br>HCHO+OH	ZPE(MP2/6-31G(d))=0.0397953<br>E(MP2/6-31G(d))=−189.1704114<br>E(UCCSD(T)/aug-cc-pVDZ)=−189.7769364<br>E(UCCSD(T)/aug-cc-pVTZ)=−189.9400030	C<br>H<br>H<br>O<br>O<br>H	0.248116<br>−0.335708<br>1.139945<br>−1.529264<br>−0.183075<br>−2.047143	0.774315<br>1.664149<br>0.856498<br>−0.686595<br>−0.380317<br>−0.944034	0.014094<br>−0.216269<br>0.633296<br>0.434322<br>−0.318201<br>−0.351524	−1338.5,202.4,349.3<br>420.5,910.9,1221.0<br>1250.0,1328.0,1587.7<br>3157.7,3283.5,3757.1	0.0432

**125**	MP2_TS_CH_4→ $CH_2(^1A_1)$+H_2	ZPE(MP2/6-31G(d))=0.0296008<br>E(MP2/6-31G(d))=−39.9994373<br>E(UCCSD(T)/aug-cc-pVDZ)=−40.1967714<br>E(UCCSD(T)/aug-cc-pVTZ)=−40.2380565	C<br>H<br>H<br>H<br>H	0.281894<br>−1.857110<br>0.917051<br>−2.120199<br>0.962351	0.364239<br>−0.401950<br>1.078784<br>−0.685831<br>−0.501438	−0.744666<br>0.860924<br>−0.182786<br>1.490366<br>−0.876165	−104.4,65.4,195.9<br>300.8,337.4,1496.8<br>3003.4,3088.0,4505.6	0.0089

Supplementary References

1 Bouilloud, M. *et al.* Bibliographic review and new measurements of the infrared band strengths of pure molecules at 25 K: H_2O, CO_2, CO, CH_4, NH_3, CH_3OH, HCOOH and H_2CO. *MNRAS* **451**, 2145-2160 (2015).

2 Elsila, J., Allamandola, L. J. & Sandford, S. A. The 2140 cm^{-1} (4.673 microns) solid CO band: The case for interstellar O_2 and N_2 and the photochemistry of nonpolar interstellar ice analogs. *Astrophys. J.* **479**, 818-838 (1997).

3 Bergantini, A., Maksyutenko, P. & Kaiser, R. I. On the formation of the C_2H_6O isomers ethanol (C_2H_5OH) and dimethyl ether (CH_3OCH_3) in star-forming regions. *Astrophys. J.* **841**, 96 (2017).

4 Drouin, D. *et al.* CASINO V2.42: a fast and easy-to-use modeling tool for scanning electron microscopy and microanalysis users. *Scanning* **29**, 92-101 (2007).

5 Dodson, L. G. *et al.* VUV photoionization cross sections of HO_2, H_2O_2, and H_2CO. *J. Phys. Chem. A* **119**, 1279-1291 (2015).

6 Tang, X. *et al.* Threshold photoelectron spectroscopy of the HO_2 radical. *J. Chem. Phys.* **153** (2020).

7 Cool, T. A., Wang, J., Nakajima, K., Taatjes, C. A. & Mcllroy, A. Photoionization cross sections for reaction intermediates in hydrocarbon combustion. *Int. J. Mass. Spectrom.* **247**, 18-27 (2005).

8 Zhu, C. *et al.* Exploitation of synchrotron radiation photoionization mass spectrometry in the analysis of complex organics in interstellar model ices. *J. Phys. Chem. Lett.* **13**, 6875-6882 (2022).

9 Meloni, G. *et al.* Energy-resolved photoionization of alkylperoxy radicals and the stability of their cations. *J. Am. Chem. Soc.* **128**, 13559-13567 (2006).

10 Bierkandt, T. *et al.* Observation of low-temperature chemistry products in laminar premixed low-pressure flames by molecular-beam mass spectrometry. *Int. J. Chem. Kinet.* **53**, 1063-1081 (2021).

11 Zhu, C. *et al.* Synthesis of methanediol [$CH_2(OH)_2$]: The simplest geminal diol. *Proc. Natl. Acad. Sci.* **119**, e2111938119 (2022).

12 Hansen, N. A. *et al.* The photoionization of methoxymethanol: Fingerprinting a reactive C_2 oxygenate in a complex reactive mixture. *J. Chem. Phys.* **160** (2024).

13 Hu, Z. *et al.* Elucidating the photodissociation fingerprint and quantifying the determination of organic hydroperoxides in gas-phase autoxidation. *Proc. Natl. Acad. Sci.* **120**, e2220131120 (2023).

14 Marks, J. H. *et al.* Methanetriol— Formation of an Impossible Molecule. *J. Am. Chem. Soc.* **146**, 12174-12184 (2024).

15 Rousso, A. C., Hansen, N., Jasper, A. W. & Ju, Y. Low-Temperature Oxidation of Ethylene by Ozone in a Jet-Stirred Reactor. *J. Phys. Chem. A* **122**, 8674-8685 (2018).

16 Moshammer, K. *et al.* Detection and Identification of the Keto-Hydroperoxide ($HOOCH_2OCHO$) and Other Intermediates during Low-Temperature Oxidation of Dimethyl Ether. *J. Phys. Chem. A* **119**, 7361-7374 (2015).

17 Cooper, G., Anderson, J. E. & Brion, C. E. Absolute photoabsorption and photoionization of formaldehyde in the VUV and soft X-ray regions (3–200 eV). *Chem. Phys.* **209**, 61-77 (1996).

18 Cool, T. A. *et al.* Selective detection of isomers with photoionization mass spectrometry for studies of hydrocarbon flame chemistry. *J. Chem. Phys.* **119**, 8356-8365 (2003).

19 Zhou, Z. Y., Xie, M. F., Wang, Z. D. & Qi, F. Determination of absolute photoionization cross-sections of aromatics and aromatic derivatives. *Rapid Commun. Mass Spectrom.* **23**, 3994-4002 (2009).

20 Cool, T. A., Wang, J., Nakajima, K., Taatjes, C. A. & McLlroy, A. Photoionization cross sections for reaction intermediates in hydrocarbon combustion. *Int. J. Mass. Spectrom.* **247**, 18-27 (2005).

21 Dong, B. *et al.* Improving quantification of hydrogen peroxide by synchrotron vacuum ultraviolet photoionization mass spectrometry. *Combust. Flame* **242**, 112214 (2022).

22 Hu, Z. H. *et al.* Elucidating the photodissociation fingerprint and quantifying the determination of organic hydroperoxides in gas-phase autoxidation. *Proc. Natl. Acad. Sci.* **120** (2023).
